# Post-diagnostic smoking cessation and heart-disease mortality among adults smoking at cardiopulmonary disease diagnosis: An NHANES linked-mortality analysis

**DOI:** 10.18332/tid/226287

**Published:** 2026-07-31

**Authors:** Dongdong Xu, Yang Liu, Huifeng Zhang, Quan Zhou, Jiangtao Huang, Rendong Li, Xuezhi Zhang

**Affiliations:** 1 Department of Thoracic Surgery, Nanchang University Affiliated Rehabilitation Hospital, Nanchang, China; 2 Department of Cardiac Surgery, Wuhan Asia Heart Hospital, Wuhan, China; 3 Department of Orthopedics, Tianyou Hospital Affiliated to Wuhan University of Science and Technology, Wuhan, China; 4 Department of Dermatology, The First Affiliated Hospital, Jiangxi Medical College, Nanchang University, Nanchang, China; 5 Department of General Surgery, Nanchang University Affiliated Rehabilitation Hospital, Nanchang, China; 6 Respiratory Diseases Key Laboratory of Jiangxi Province, Nanchang, China; 7 Department of Neurosurgery, Nanchang University Affiliated Rehabilitation Hospital, Nanchang, China

**Keywords:** post-diagnostic smoking cessation, smoking at diagnosis, heart-disease mortality, secondary prevention, NHANES

## Abstract

**INTRODUCTION:**

Population-based analyses usually classify smoking status at survey enrollment, which combines people who quit before disease recognition with those who continued smoking through diagnosis and quit afterward. This study evaluated whether smoking cessation after a self-reported cardiopulmonary disease diagnosis was associated with subsequent all-cause and cause-specific mortality among adults reconstructed as smoking at diagnosis.

**METHODS:**

This secondary analysis pooled ten cross-sectional National Health and Nutrition Examination Survey (NHANES) cycles from 1999–2000 through 2017–2018 and linked survey records to mortality follow-up through 2019. Adults aged ≥40 years with self-reported cardiovascular or chronic lung disease were included when self-reported smoking initiation, diagnosis, cessation, and current-smoking information permitted classification as smoking at diagnosis. Survey-weighted Cox models compared post-diagnostic quitters with persistent smokers. Models were unadjusted, demographic-adjusted, and fully adjusted for prespecified demographic, disease-history, smoking-history, and survey-cycle covariates.

**RESULTS:**

Among 2319 participants, 975 were post-diagnostic quitters, and 1344 were persistent smokers. During a median follow-up of 6.33 years, 999 all-cause, 272 heart-disease, and 244 cancer deaths occurred. In the fully adjusted model, post-diagnostic quitting was associated with lower heart-disease mortality (adjusted hazard ratio, AHR=0.59; 95% CI: 0.42–0.82; p=0.001). Associations with all-cause mortality (AHR=0.90; 95% CI: 0.74–1.09; p=0.276) and cancer mortality (AHR=0.72; 95% CI: 0.49–1.06; p=0.100) were not statistically significant. Findings for heart-disease mortality were consistent across landmark, overlap-weighted, smoking-burden-adjusted, disease-restricted, quit-age reconstruction, and leave-one-covariate-out analyses.

**CONCLUSIONS:**

Among adults retrospectively classified as smoking when cardiopulmonary disease was diagnosed, post-diagnostic cessation was associated with lower subsequent heart-disease mortality. The findings are observational and remain susceptible to survivor selection, reverse causation, recall and social-desirability bias, exposure changes after enrollment, mortality misclassification, and residual confounding.

## INTRODUCTION

Tobacco smoking remains a major preventable cause of premature morbidity and mortality worldwide^1-3^. Although cigarette smoking has declined in the United States, millions of adults continue to smoke, and smoking remains concentrated among people with chronic diseases attributable to tobacco exposure^4-6^. For patients with established cardiovascular or chronic pulmonary disease, continued smoking compounds endothelial injury, thrombosis, autonomic activation, inflammation, and progressive respiratory impairment^7-9^.

Across diverse populations, smoking cessation is associated with lower subsequent mortality, with risk differences emerging within the first years after quitting and widening over time^10-14^. Cardiovascular risk declines with longer time since cessation, although it may remain above that of never smokers for many years^15^. Among patients with coronary disease, cessation has been associated with lower mortality and recurrent cardiovascular events^16,17^. Similar associations have been reported among patients with cardiovascular disease and chronic obstructive pulmonary disease (COPD)^18,19^. These findings support smoking cessation as a central component of secondary prevention.

A limitation of many population-based analyses is that smoking status is defined at study enrollment. A participant classified as a former smoker may have quit before the diagnosis that motivates secondary prevention, whereas another may have continued smoking through diagnosis and quit afterward. Diagnosis itself is an important trigger for behavior change^20,21^. Recent NHANES studies have described smoking status and mortality in general populations or among people with self-reported COPD^14,22,23^, but did not reconstruct whether smoking continued through the time of cardiopulmonary diagnosis. Randomized and longitudinal evidence in COPD also indicates slower lung-function decline and more favorable long-term outcomes after cessation^24-26^.

The present analysis used self-reported ages at regular smoking initiation, first cardiopulmonary diagnosis, and smoking cessation to reconstruct smoking status at diagnosis. The objective was to estimate associations of post-diagnostic quitting, compared with persistent smoking, with subsequent all-cause, heart-disease, and cancer mortality. It was hypothesized that post-diagnostic quitting would be associated with lower subsequent mortality, with the clearest association for heart-disease mortality. The stability of the heart-disease estimate was evaluated across landmark, weighting, covariate, disease-restricted, quit-age reconstruction, and collinearity sensitivity analyses.

## METHODS

### Study design and data source

This secondary analysis pooled ten cross-sectional NHANES cycles from 1999–2000 through 2017–2018 and linked the survey records to prospective mortality follow-up through 31 December 2019 using the National Center for Health Statistics 2019 public-use Linked Mortality Files. NHANES uses a stratified, multistage probability design to represent the US civilian, non-institutionalized population^27-30^. Reporting followed the Strengthening the Reporting of Observational Studies in Epidemiology (STROBE) statement^31^.

### Study population

**Figure 1:**
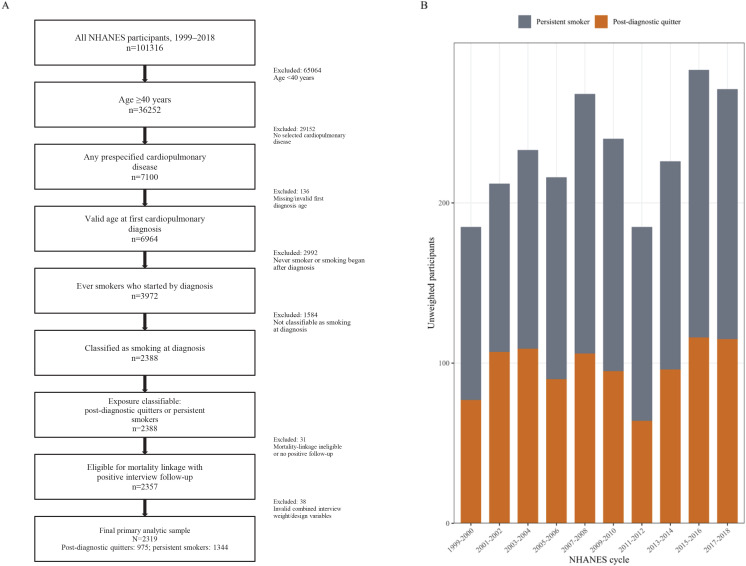
Construction and survey-cycle composition of the diagnosis-anchored analytic sample in a secondary analysis of pooled NHANES 1999–2018 linked-mortality data, United States (N=2319): A) Stepwise participant flow from the ten pooled cross-sectional NHANES cycles to the final analytic sample. Counts are unweighted; B) Unweighted numbers of persistent smokers and post-diagnostic quitters in each survey cycle

Participants aged at least 40 years who reported congestive heart failure, coronary heart disease, angina, myocardial infarction, emphysema, or chronic bronchitis were considered. The lower age boundary was prespecified to focus on midlife and older adults with established cardiopulmonary disease and is consistent with diagnosis-based COPD research that enrolled adults aged ≥40 years^19^. For participants reporting more than one condition, age at first cardiopulmonary diagnosis was the earliest valid diagnosis age. Eligibility additionally required a valid age at initiation of regular smoking, smoking initiation no later than the diagnosis age, sufficient smoking-history information to determine smoking status at diagnosis, mortality-linkage eligibility, positive follow-up time, and valid interview weights and survey design variables. The sequential exclusions are shown in Figure 1A.

### Reconstruction of smoking status at diagnosis and post-diagnostic quitting

All smoking variables were self-reported. Ever smoking was defined as lifetime consumption of at least 100 cigarettes. Current smoking at the NHANES interview was defined as smoking every day or some days; former smoking was defined as smoking not at all among ever smokers. The NHANES variable SMD055 records the self-reported age at which a former smoker completely stopped smoking cigarettes. When SMD055 was unavailable, cessation age was derived from current age and the reported duration since quitting. Smoking at diagnosis was reconstructed by comparing age at regular smoking initiation, age at first cardiopulmonary diagnosis, age at cessation, and smoking status at the NHANES interview.

Post-diagnostic quitters were former smokers who had been smoking at diagnosis and whose reported or derived cessation age was at or after diagnosis. Persistent smokers had been smoking at diagnosis and remained current smokers at the NHANES interview. Participants who could not be assigned unambiguously to either group were excluded. Persistent smokers were the reference group in all regression models.

### Mortality outcomes

All-cause, heart-disease, and cancer mortality were evaluated for the full 1999–2018 analytic period. Heart-disease mortality corresponded to public-use leading cause-of-death category 001 and cancer mortality to category 002. Because cerebrovascular and chronic lower respiratory disease categories were not separately available for later cycles, cardiovascular composite mortality (heart disease plus cerebrovascular disease) and chronic lower respiratory disease mortality were evaluated in analyses restricted to NHANES 1999–2014. Follow-up accrued from the NHANES interview until death or 31 December 2019. Outcome counts and cycle availability are provided in the Supplementary file.

### Covariates

Covariates were selected a priori from established determinants of smoking cessation and mortality and from variables available consistently across NHANES cycles^4-9,14-19^. The fully adjusted core model included age (per 10 years), sex (male or female), race and ethnicity (non-Hispanic White, Hispanic, non-Hispanic Black, or Other/multi-racial), education level (high school or lower, some college or higher, or unknown), cardiopulmonary disease phenotype (cardiovascular disease only, chronic lung disease only, both disease classes, or other/uncategorized), years since first diagnosis (per 5 years), years of regular smoking before diagnosis (per 10 years), and centered two-year survey cycle. Extended analyses additionally incorporated body mass index, poverty-income ratio, self-reported diabetes, self-reported hypertension, and a smoking-burden proxy based on smoking duration and cigarettes smoked per day. Definitions and missingness are detailed in the Supplementary file.

### Statistical analysis

All descriptive and regression analyses accounted for NHANES strata, primary sampling units, and interview or examination weights as appropriate. Interview weights were used for the core analyses because the exposure, disease history, and core covariates were obtained from interview components; examination weights were used when body mass index and other examination-derived variables were introduced. Combined 20-year interview weights were constructed by dividing the 1999–2002 four-year interview weight by five and each later two-year interview weights by ten. Corresponding 18-year and 16-year weights were constructed for restricted-period analyses. Strata and primary sampling unit identifiers were made unique across survey cycles. The original NHANES weights were not recalibrated for mortality-linkage eligibility.

Baseline characteristics were summarized as survey-weighted means with standard deviations or unweighted counts with survey-weighted percentages. Absolute standardized differences (ASDs) quantified baseline imbalance; an ASD ≥0.10 indicated meaningful imbalance.

Survey-weighted Cox proportional hazards models were fitted with design-based variance estimation^32^. Model 0 was unadjusted. Model 1 adjusted for age, sex, race and ethnicity, and education level. Model 2 was the fully adjusted core model and additionally included disease phenotype, years since diagnosis, pre-diagnosis smoking duration, and survey cycle. Hazard ratios compare post-diagnostic quitters with persistent smokers. The three full-period outcomes were interpreted using nominal p-values and a conservative Bonferroni threshold of 0.0167.

Multicollinearity was assessed using conventional variance inflation factors (VIFs) from the covariate design matrix. VIF values 5–10 were interpreted as moderate multicollinearity and values >10 as potentially serious. For multi-degree-of-freedom factors, generalized VIFs and GVIF^(1/2df)^ were also reported. Leave-one-covariate-out models separately omitted years since diagnosis and pre-diagnosis smoking duration. Detailed diagnostics are provided in the Supplementary file.

Robustness analyses restricted the survey period to 1999–2016, required cessation age to exceed diagnosis age, introduced examination-weighted extended covariates, additionally adjusted for smoking burden, restricted the population to cardiovascular or chronic lung disease, and excluded deaths within 12, 24, or 36 months after the NHANES interview. Propensity scores were estimated from the core demographic and disease-history covariates, and survey weights were multiplied by overlap weights to emphasize participants with comparable exposure probabilities^33^. Quit-age reconstruction analyses excluded quitters whose reported and derived cessation ages differed by more than 1, 2, or 5 years and repeated the analysis among quitters with directly reported SMD055 ages only. The discrepancy distribution and analytic sample counts are provided in the Supplementary file.

Exploratory subgroup analyses were conducted by age group, sex, and disease phenotype. Interaction terms were tested with design-based Wald tests. A Bonferroni threshold of 0.0167 was used for the three interaction tests. The proportional hazards assumption was assessed using scaled Schoenfeld residuals^34,35^. Term-level tests preserved the degrees of freedom of multilevel categorical variables. A two-sided p<0.05 was considered nominally significant unless otherwise specified. Complete-case analysis was used within each model; core-model variables were complete in the analytic sample. Analyses were performed in R version 4.6.0.

### Ethics

The NHANES protocols were approved by the National Center for Health Statistics Research Ethics Review Board, and all participants provided written informed consent. This analysis used publicly available de-identified data and did not require additional institutional review board approval.

## RESULTS

### Study population and baseline characteristics

Among 101316 participants in the pooled NHANES cycles, 2319 met the diagnosis-anchored eligibility criteria (Figure 1A). The analytic sample included 975 post-diagnostic quitters and 1344 persistent smokers, with both groups represented in every survey cycle (Figure 1B). Post-diagnostic quitters were older than persistent smokers (weighted mean 64.41 vs 57.19 years; ASD=0.679) and had more years between first cardiopulmonary diagnosis and the NHANES interview (20.80 vs 12.22 years; ASD=0.720). Other large ASDs involved age group (0.539), cigarettes per day (0.504), pack-years (0.335), body mass index (0.330), poverty-income ratio (0.325), diabetes (0.263), and sex (0.196) (Table 1).

**Table 1:** Baseline characteristics by diagnosis-anchored smoking status in a secondary analysis of pooled NHANES 1999–2018 linked-mortality data, United States (N=2319)

*Characteristic*		*Persistent smokers* *(N=1344)*	*Post-diagnostic* *quitters* *(N=975)*	*Absolute standardized difference*
		*Mean ± SD*	*Mean ± SD*	
Age (years)		57.19 ± 10.26	64.41 ± 11.01	**0.679**
NHANES survey midyear		2008.95 ± 5.81	2009.08 ± 5.93	0.023
Years from first cardiopulmonary diagnosis to NHANES		12.22 ± 10.50	20.80 ± 13.19	**0.72**
Regular-smoking duration before diagnosis (years)		27.90 ± 13.52	26.58 ± 14.84	0.093
Family poverty-to-income ratio		2.18 ± 1.51	2.68 ± 1.56	**0.325**
Cigarettes per day proxy		17.88 ± 13.35	25.87 ± 18.02	**0.504**
Pack-years proxy		35.47 ± 27.45	46.42 ± 37.25	**0.335**
Body mass index (kg/m^2^)		28.44 ± 7.01	30.77 ± 7.10	**0.33**
	***Categories***	***n (%)***	***n (%)***	
Sex	Male	690 (44.7)	600 (54.5)	**0.196**
	Female	654 (55.3)	375 (45.5)	**0.196**
Race and ethnicity	Non-Hispanic White	775 (75.9)	620 (83.3)	**0.184**
	Hispanic	159 (4.8)	136 (4.8)	0.001
	Non-Hispanic Black	321 (11.8)	170 (7.6)	**0.143**
	Other/multi-racial	89 (7.5)	49 (4.3)	**0.135**
Education level	High school or lower	907 (63.7)	609 (57.3)	**0.13**
	Some college or higher	436 (36.3)	364 (42.5)	**0.128**
	Unknown	1 (0.0)	2 (0.1)	0.04
Cardiopulmonary disease phenotype	Cardiovascular disease only	534 (34.6)	429 (38.1)	0.072
	Chronic lung disease only	595 (50.0)	352 (41.7)	**0.165**
	Both cardiovascular and lung disease	214 (15.4)	194 (20.2)	**0.125**
	Other/uncategorized	1 (0.0)	0 (0.0)	0.02
Diabetes	No	1011 (80.4)	642 (69.0)	**0.263**
	Yes	295 (17.5)	302 (28.2)	**0.255**
	Unknown	38 (2.1)	31 (2.9)	0.049
Hypertension	No	527 (45.5)	346 (40.7)	0.097
	Yes	816 (54.4)	627 (59.1)	0.095
	Unknown	1 (0.1)	2 (0.2)	0.033
Age group (years)	40–64	909 (75.5)	381 (49.4)	**0.539**
	≥65	435 (24.5)	594 (50.6)	**0.539**

Values are survey-weighted means (SD) or unweighted counts (survey-weighted percentages). The absolute standardized difference (ASD) is the difference in means or proportions divided by a pooled standard deviation; an absolute ASD ≥0.10 indicates meaningful imbalance and is shown in bold. CVD: cardiovascular disease. NHANES: National Health and Nutrition Examination Survey.

### Associations with all-cause and cause-specific mortality

**Figure 2:**
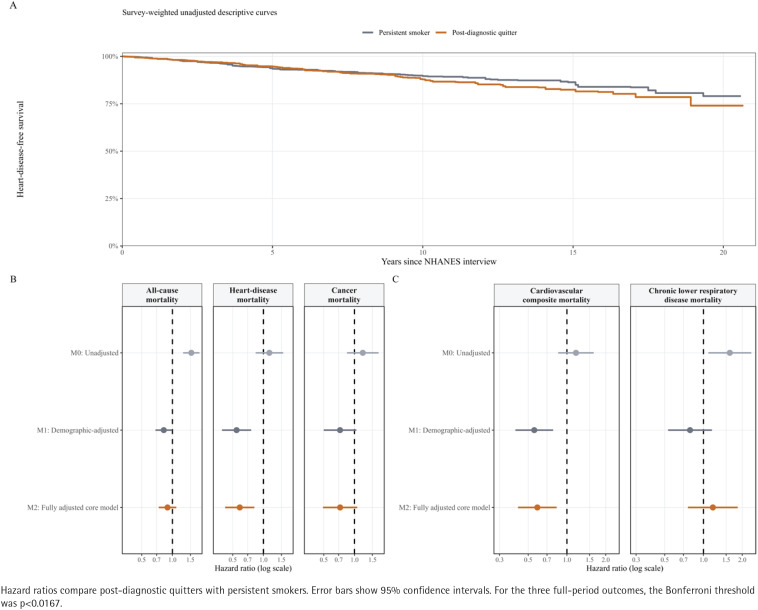
Post-diagnostic smoking cessation and mortality in a secondary analysis of pooled NHANES 1999–2018 linked-mortality data, United States (N=2319): A) Survey-weighted unadjusted descriptive Kaplan-Meier curves for heart-disease-free survival; B) Unadjusted, demographic-adjusted, and fully adjusted survey-weighted Cox estimates for all-cause, heart-disease, and cancer mortality in NHANES 1999-2018; C) Corresponding estimates for cardiovascular composite and chronic lower respiratory disease mortality in analyses restricted to NHANES 1999–2014 (N=1765)

During a median follow-up of 6.33 years, 999 allcause, 272 heart-disease, and 244 cancer deaths occurred. For all-cause mortality, the unadjusted HR was 1.53 (95% CI: 1.27–1.85; p<0.001), the demographic-adjusted HR (AHR) was 0.82 (95% CI: 0.68–0.99; p=0.043), and the fully adjusted AHR was 0.90 (95% CI: 0.74–1.09; p=0.276). For heart-disease mortality, the corresponding HRs were 1.14 (95% CI: 0.84–1.56; p=0.398), 0.55 (95% CI: 0.39–0.76; p<0.001), and 0.59 (95% CI: 0.42–0.82; p=0.001). For cancer mortality, the HRs were 1.21 (95% CI: 0.85–1.72; p=0.301), 0.72 (95% CI: 0.50–1.04; p=0.084), and 0.72 (95% CI: 0.49–1.06; p=0.100) (Table 2 and Figures 2A and 2B). The fully adjusted heart-disease association remained statistically significant at the Bonferroni threshold of 0.0167.

**Table 2:** Survey-weighted Cox models for mortality outcomes in pooled NHANES linked-mortality data, United States: full-period analyses (1999–2018; N=2319) and restricted-period analyses (1999–2014; N=1765)

*Outcome*	*Total* *n*	*Deaths*	*Model 0* *HR (95% CI); p*	*Model 1* *AHR (95% CI); p*	*Model 2* *AHR (95% CI); p*
**NHANES 1999–2018**					
All-cause mortality	2319	999	1.53 (1.27–1.85); <0.001	0.82 (0.68–0.99); 0.043	0.90 (0.74–1.09); 0.276
Heart-disease mortality	2319	272	1.14 (0.84–1.56); 0.398	0.55 (0.39–0.76); <0.001	0.59 (0.42–0.82); 0.001
Cancer mortality	2319	244	1.21 (0.85–1.72); 0.301	0.72 (0.50–1.04); 0.084	0.72 (0.49–1.06); 0.100
**NHANES 1999–2014**					
Cardiovascular composite mortality (heart + cerebrovascular)	1765	283	1.17 (0.86–1.61); 0.319	0.56 (0.40–0.78); <0.001	0.59 (0.42–0.83); 0.003
Chronic lower respiratory disease mortality	1765	152	1.60 (1.09–2.35); 0.016	0.79 (0.53–1.16); 0.229	1.18 (0.76–1.84); 0.461

Hazard ratios compare post-diagnostic quitters with persistent smokers (reference). Model 0: unadjusted model. Model 1 adjusted for age, sex, race and ethnicity, and education level. Model 2 additionally adjusted for cardiopulmonary disease phenotype, years since first diagnosis, pre-diagnosis smoking duration, and survey cycle. AHR: adjusted hazard ratio. For the three 1999–2018 outcomes, the Bonferroni threshold was p<0.0167.

In the 1999–2014 restricted analysis, the fully adjusted AHR was 0.59 (95% CI: 0.42–0.83; p=0.003) for cardiovascular composite mortality and 1.18 (95% CI: 0.76–1.84; p=0.461) for chronic lower respiratory disease mortality (Table 2 and Figure 2C).

### Sensitivity, landmark, and collinearity analyses

**Figure 3:**
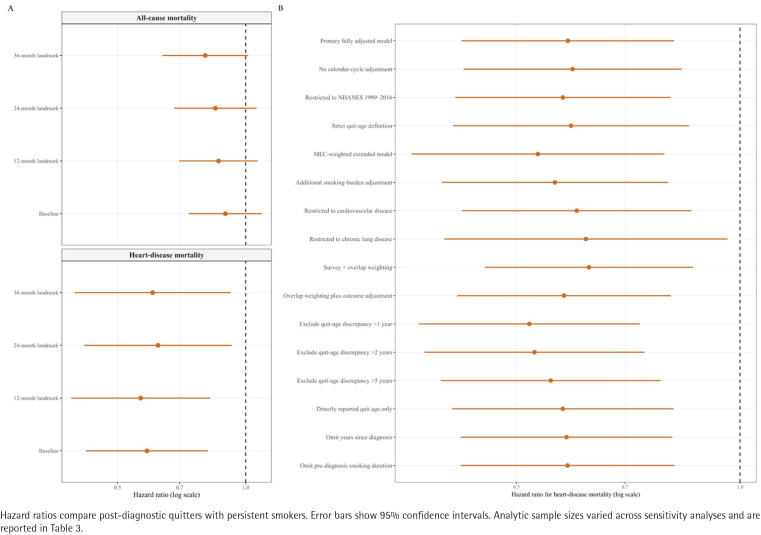
Landmark and sensitivity analyses in a secondary analysis of pooled NHANES 1999–2018 linked-mortality data, United States: A) Fully adjusted all-cause and heart-disease mortality estimates after 12-, 24-, and 36-month landmark exclusions; B) Heart-disease mortality estimates across alternative survey periods, exposure definitions, covariate specifications, disease restrictions, overlap weighting, quit-age reconstruction, and leave-one-covariate-out analyses

After exclusion of deaths within 12, 24, and 36 months, the fully adjusted heart-disease AHRs were 0.57 (95% CI: 0.39–0.82; p=0.003), 0.62 (95% CI: 0.42–0.93; p=0.020), and 0.60 (95% CI: 0.40–0.92; p=0.019), respectively (Table 3 and Figure 3A). Across alternative survey periods, exposure definitions, covariate specifications, disease restrictions, overlap weighting, and quit-age reconstruction analyses, heart-disease AHRs ranged from 0.52 to 0.63 (Table 3 and Figure 3B).

**Table 3:** Sensitivity, landmark, overlap-weighted, quit-age, and leave-one-covariate-out analyses in pooled NHANES 1999–2018 linked-mortality data, United States

*Analysis*	*n*	*All-cause* *deaths*	*All-cause* *HR (95% CI); p*	*Heart-disease* *deaths*	*Heart-disease* *AHR (95% CI); p*
**Conventional and landmark sensitivity**					
Primary 1999–2018 without calendar-cycle adjustment	2319	999	0.90 (0.74–1.09); 0.280	272	0.60 (0.42–0.83); 0.003
Restricted 1999–2016 with 18-year combined interview weights	2048	971	0.90 (0.74–1.10); 0.310	264	0.58 (0.41–0.81); 0.001
Strict exposure: quit age > diagnosis age	2093	877	0.99 (0.79–1.24); 0.945	235	0.59 (0.41–0.85); 0.005
MEC-weighted extended complete-case model	1943	801	0.95 (0.77–1.18); 0.645	212	0.53 (0.36–0.79); 0.002
Smoking-burden proxy complete-case sensitivity	2239	951	0.87 (0.70–1.07); 0.184	260	0.56 (0.40–0.80); 0.001
Restricted to participants with cardiovascular disease	1371	658	0.83 (0.66–1.05); 0.122	213	0.60 (0.42–0.86); 0.005
Restricted to participants with chronic lung disease	1356	570	1.03 (0.81–1.33); 0.790	136	0.62 (0.40–0.96); 0.033
Landmark at 12 months/M2_core	2196	876	0.86 (0.70–1.07); 0.174	233	0.57 (0.39–0.82); 0.003
Landmark at 24 months/M2_core	1958	768	0.85 (0.68–1.06); 0.149	205	0.62 (0.42–0.93); 0.020
Landmark at 36 months/M2_core	1742	662	0.80 (0.64–1.01); 0.064	175	0.60 (0.40–0.92); 0.019
**Overlap-weighted supportive analysis**					
Survey × overlap-weighted supportive analysis	2319	999	0.95 (0.78–1.16); 0.633	272	0.63 (0.45–0.86); 0.004
Survey × overlap-weighted doubly adjusted sensitivity	2319	999	0.90 (0.74–1.09); 0.270	272	0.58 (0.42–0.81); 0.001
**Quit-age reconstruction sensitivity**					
Quit-age discrepancy exclusion >1 years	2199	917	0.84 (0.68–1.04); 0.110	249	0.52 (0.37–0.73); <0.001
Quit-age discrepancy exclusion >2 years	2236	941	0.85 (0.69–1.05); 0.124	252	0.53 (0.38–0.74); <0.001
Quit-age discrepancy exclusion >5 years	2279	967	0.87 (0.72–1.07); 0.193	261	0.56 (0.40–0.78); <0.001
Quitters restricted to directly reported SMD055 quit age	2117	936	0.87 (0.70–1.08); 0.203	258	0.58 (0.41–0.81); 0.002
**Collinearity sensitivity**					
Fully adjusted model without years since diagnosis	2319	999	0.90 (0.74–1.10); 0.298	272	0.58 (0.42–0.81); 0.001
Fully adjusted model without pre-diagnosis smoking duration	2319	999	0.89 (0.73–1.09); 0.249	272	0.59 (0.42–0.82); 0.002

Hazard ratios compare post-diagnostic quitters with persistent smokers. Each row summarizes separate survey-weighted Cox models for all-cause and heart-disease mortality in the stated analytic sample. The main fully adjusted model included age, sex, race and ethnicity, education level, disease phenotype, years since diagnosis, pre-diagnosis smoking duration, and survey cycle unless a row explicitly omitted one covariate. AHR: adjusted hazard ratio. MEC: mobile examination center.

The largest individual VIFs were 7.08 for pre-diagnosis smoking duration and 6.03 for years since diagnosis; no VIF exceeded 10. Omitting years since diagnosis produced a heart-disease AHR of 0.58 (95% CI: 0.42–0.81; p=0.001), and omitting pre-diagnosis smoking duration produced an AHR of 0.59 (95% CI: 0.42–0.82; p=0.002). Corresponding all-cause estimates were 0.90 (95% CI: 0.74–1.10; p=0.298) and 0.89 (95% CI: 0.73–1.09; p=0.249) (Table 3; and Supplementary file).

### Overlap weighting and exploratory subgroup analyses

**Figure 4:**
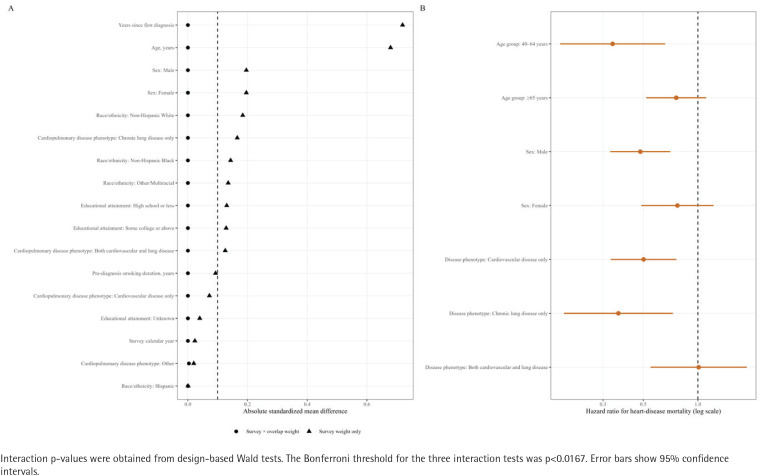
Covariate balance and exploratory subgroup analyses in a secondary analysis of pooled NHANES 1999–2018 linked-mortality data, United States (N=2319): A) Absolute standardized differences for variables included in the propensity-score model before and after multiplication of survey and overlap weights; the dashed line denotes the prespecified 0.10 threshold; B) Fully adjusted heart-disease mortality estimates by age, sex, and disease phenotype

Among variables included in the propensity-score model, the maximum absolute ASD decreased from 0.720 with survey weights alone to 0.003 after multiplication by overlap weights (Figure 4A; and Supplementary file). The survey-by-overlap-weighted heart-disease AHR was 0.63 (95% CI: 0.45–0.86; p=0.004), and the doubly adjusted overlap-weighted AHR was 0.58 (95% CI: 0.42–0.81; p=0.001) (Table 3).

For participants aged 40–64 years and ≥65 years, the heart-disease AHRs were 0.34 (95% CI: 0.17–0.66; p=0.001) and 0.76 (95% CI: 0.52–1.11; p=0.156), respectively; the interaction p was 0.101. The AHRs were 0.48 (95% CI: 0.33–0.71; p<0.001) for men and 0.77 (95% CI: 0.49–1.22; p=0.266) for women; the interaction p was 0.024. Disease-phenotype AHRs were 0.50 (95% CI: 0.33–0.76; p=0.001) for cardiovascular disease only, 0.36 (95% CI: 0.18–0.73; p=0.004) for chronic lung disease only, and 1.01 (95% CI: 0.55–1.86; p=0.973) for both disease classes; the interaction p was 0.129 (Table 4 and Figure 4B). None of the three interaction tests met the Bonferroni threshold of 0.0167.

**Table 4:** Exploratory subgroup analyses of heart-disease mortality in pooled NHANES 1999–2018 linked-mortality data, United States (N=2319)

*Variables*	*n*	*Deaths*	*AHR (95% CI)*	*p*	*p for interaction*
**Age** (years)					
40–64	1290	94	0.34 (0.17–0.66)	0.001	0.101
≥65	1029	178	0.76 (0.52–1.11)	0.156	0.101
**Sex**					
Male	1290	171	0.48 (0.33–0.71)	<0.001	0.024
Female	1029	101	0.77 (0.49–1.22)	0.266	0.024
**Disease phenotype**					
Cardiovascular disease only	963	136	0.50 (0.33–0.76)	0.001	0.129
Chronic lung disease only	947	59	0.36 (0.18–0.73)	0.004	0.129
Both cardiovascular and lung disease	408	77	1.01 (0.55–1.86)	0.973	0.129

Hazard ratios compare post-diagnostic quitters with persistent smokers and were adjusted for the core-model covariates other than the stratifying variable. Interaction p values were obtained from design-based Wald tests. The Bonferroni threshold for the three interaction tests was p<0.0167. AHR: adjusted hazard ratio.

### Proportional hazards assessment

The exposure-specific proportional hazards p-values were 0.065 for all-cause mortality, 0.569 for heart-disease mortality, and 0.264 for cancer mortality. The corresponding global p-values were 0.027, 0.174, and 0.635 (Table 5; and Supplementary file).

**Table 5:** Proportional hazards assessment for fully adjusted mortality models in pooled NHANES 1999–2018 linked-mortality data, United States (N=2319)

*Outcome*	*n*	*Deaths*	*Exposure-* *specific p*	*Global p*
All-cause mortality	2319	999	0.065	0.027
Heart-disease mortality	2319	272	0.569	0.174
Cancer mortality	2319	244	0.264	0.635

P-values were obtained from scaled Schoenfeld residual tests. A p<0.05 indicates statistical evidence against proportional hazards. Term-level global tests preserved the degrees of freedom of multilevel categorical variables.

## DISCUSSION

This nationally representative analysis found that post-diagnostic smoking cessation was associated with lower subsequent heart-disease mortality among adults reconstructed as smoking when cardiovascular or chronic lung disease was diagnosed. Associations with all-cause and cancer mortality were not statistically significant. The principal methodological contribution is the use of diagnosis as the temporal anchor, which separates cessation after disease recognition from cessation that occurred before diagnosis.

The reversal from an adverse unadjusted estimate to a lower adjusted heart-disease estimate illustrates the extent of confounding in comparisons between former and current smokers. Participants who quit after diagnosis were older and had survived longer from diagnosis to survey participation than persistent smokers. Adjustment, overlap weighting, and landmark exclusion reduced measured imbalance and the influence of early deaths, but cannot remove left truncation, survivor selection, residual confounding, or reverse causation.

The heart-disease finding is consistent with evidence that smoking cessation improves cardiovascular prognosis among people with coronary or other cardiovascular disease and with evidence of reduced mortality after cessation in COPD^16-19^. However, previous studies have differed in population composition, disease severity, sex distribution, exposure timing, availability of time-updated smoking status, covariate adjustment, and follow-up duration. These differences can explain why the magnitude and statistical significance of reported associations are not uniform across studies.

The absence of a statistically significant cancer-mortality association should not be interpreted as evidence of no benefit. Cancer risk may decline more slowly after cessation than cardiovascular risk, and the interval from quitting to NHANES enrollment varied substantially. The cancer analysis also had fewer events than the all-cause analysis, combined multiple cancer sites with different smoking-related latency, and relied on broad public-use cause-of-death classification. These features reduce precision and may attenuate an association within the available follow-up.

The all-cause result may reflect heterogeneous cause-specific associations, residual differences between exposure groups, and non-proportionality in at least one covariate of the all-cause model. Because the smoking-cessation coefficient itself did not show statistical evidence of non-proportionality, the all-cause HR can be viewed as an average association over follow-up, but it should be interpreted cautiously.

### Strengths and limitations

Strengths include the use of ten NHANES cycles, complex-survey methods, linked mortality follow-up, explicit cross-cycle weight construction, a clinically interpretable diagnosis anchor, and multiple prespecified robustness analyses. These features support the consistency of the observed heart-disease association but do not establish causality.

Several limitations define the scope of inference. First, participants with incomplete smoking or diagnosis histories were excluded, creating potential selection bias. Second, diagnosis and cessation ages, smoking intensity, and current smoking status were self-reported and susceptible to recall and social-desirability bias. Third, participants with worsening health may have been more likely to quit, producing sick-quitter reverse causation, while people had to survive from diagnosis to NHANES enrollment to enter the analysis, producing left truncation and survivor selection. Fourth, smoking status could have changed after NHANES enrollment, resulting in exposure misclassification. Fifth, disease severity, treatment, medication adherence, rehabilitation, alcohol use, and physical activity could not be harmonized and temporally aligned across all cycles. Sixth, the combined cardiopulmonary population was heterogeneous and subgroup analyses were imprecise. Seventh, public-use mortality categories are broad and may misclassify specific causes of death. Finally, residual and unmeasured confounding cannot be excluded. The net direction and magnitude of these biases are uncertain.

## CONCLUSIONS

Among adults who were retrospectively classified as smokers when cardiopulmonary disease was diagnosed, post-diagnostic cessation was associated with lower subsequent heart-disease mortality. Prospective studies with diagnosis-time enrollment, repeated smoking measures, disease-severity information, and treatment data are needed to clarify the magnitude, timing, and causal interpretation of this association.

## Supplementary Material



## Data Availability

NHANES survey data and the 2019 public-use Linked Mortality Files are publicly available from the National Center for Health Statistics. The exact data files and variable definitions used in this analysis are documented in the Supplementary file. The complete R analysis scripts are provided as Supplementary file Code, and numerical data underlying Figures 1–4 are provided as Supplementary file Figure Source Data.
