# Supplementary material for: Post-diagnostic smoking cessation and heart-disease mortality among adults smoking at cardiopulmonary disease diagnosis: An NHANES linked-mortality analysis
Source: Tob Induc Dis. 2026 Jul 31;24:10.18332/tid/226287. doi: 10.18332/tid/226287 (PMC13428304; doi:10.18332/tid/226287)
Supplement: Supplementary file 1 [file TID-24-134-s1.pdf]

TID
Tobacco Induced Diseases

Supplementary File

© 2026 Xu D. et al.

DOI:
10.18332/tid/226287

The content has been provided by the author(s) and has not been reviewed, verified, or endorsed by European Publishing. It may not have undergone peer review. The views, opinions, and recommendations expressed are solely those of the author(s) and do not necessarily reflect the position of European Publishing. European Publishing accepts no responsibility or liability for any consequences arising from the use of, or reliance on, this content.

Post-diagnostic smoking cessation and heart-disease mortality among adults smoking at cardiopulmonary disease diagnosis: An NHANES linked-mortality analysis

Supplementary methods

Cross-cycle data harmonization

Within each NHANES cycle, demographic, medical-conditions, smoking, body-measures, blood-pressure, and diabetes files were merged by participant identifier (SEQN) before linkage to the cycle-specific 2019 public-use mortality file. Variable names and labelled values were standardized across cycles. Cycle-level audits evaluated sample size, smoking-variable recoding, survey weights, harmonized variables, mortality-linkage eligibility, and cause-of-death availability.

Reconstruction of diagnosis and smoking timelines

Age at first cardiopulmonary diagnosis was the minimum valid age reported for congestive heart failure, coronary heart disease, angina, myocardial infarction, emphysema, or chronic bronchitis. Regular-smoking initiation age was required to be no later than diagnosis age. The NHANES variable SMD055 records the self-reported age at which a former smoker completely stopped smoking cigarettes and was used directly when available. Otherwise, cessation age was derived from interview age and reported duration since quitting. The code 66666 in SMQ050Q, indicating at least 50 years since quitting, was not used to derive an exact cessation age.

Combined survey weights and design variables

For the 20-year pooled analysis, the 1999-2002 four-year interview weight was divided by five and each later two-year interview weight by ten. For the 1999-2016 sensitivity analysis, the four-year weight was multiplied by 2/9 and later weights were divided by nine. For 1999-2014 analyses, the four-year weight was divided by four and later weights by eight. Survey strata and primary sampling unit identifiers were nested within survey start year. Original NHANES weights were not recalibrated for mortality-linkage eligibility.

Mortality outcome availability

Heart-disease mortality (public-use category 001) and cancer mortality (002) were available across NHANES 1999-2018. Cerebrovascular disease (005) and chronic lower respiratory disease (003) were not separately identifiable for 2015-2018; therefore, cardiovascular composite and chronic lower respiratory disease analyses were limited to NHANES 1999-2014.

Overlap weighting and covariate balance

A propensity score for post-diagnostic quitting was estimated from the prespecified core demographic and disease-history covariates. Overlap weights were defined as 1 minus the propensity score for quitters and as the propensity score for persistent smokers, and were multiplied by NHANES interview weights. Covariate balance was summarized using absolute standardized mean differences. Poverty-income ratio was not included in the core propensity-score model and is not displayed in the final Figure 4A balance panel.

Landmark and exposure-reconstruction analyses

Landmark analyses excluded participants whose follow-up ended within 12, 24, or 36 months and reset analysis time after the landmark. Quit-age analyses excluded quitters whose directly reported and derived cessation ages differed by more than 1, 2, or 5 years. A stricter analysis retained persistent smokers and only quitters with a directly reported SMD055 cessation age.

Multicollinearity diagnostics

Multicollinearity was assessed from the fully adjusted covariate design matrix. Conventional VIF values of 5-10 were interpreted as moderate and values above 10 as potentially serious. For multi-degree-of-freedom factors, raw generalized VIFs and GVIF^(1/[2 x df]) were reported. Leave-one-covariate-out survey-weighted Cox models separately omitted years since diagnosis and pre-diagnosis smoking duration.

Proportional hazards diagnostics

The proportional hazards assumption was assessed using scaled Schoenfeld residuals. Term-level tests retained the degrees of freedom of multilevel categorical variables. Exposure-specific and global tests were reported separately for the fully adjusted all-cause, heart-disease, and cancer mortality models.

Model quality control

Each fitted model was audited for model status, sample size, event count, representation of both exposure groups, finite estimates, confidence-interval width, and extreme P values. The residual Other/uncategorized disease subgroup contained one participant, no quitter, and no heart-disease death and was not estimated as a subgroup-specific model.

Supplementary results

The fully adjusted core variables were complete in the analytic sample. Poverty-income ratio was missing for 8.93%, body mass index for 8.11%, pack-years for 3.45%, and cigarettes per day for 3.41% of participants (Table S4).

Among 975 post-diagnostic quitters, 772 had both direct and derived cessation ages. The median absolute discrepancy was 0 years (interquartile range 0-1); 120 differed by more than 1 year, 83 by more than 2 years, and 40 by more than 5 years (Tables S7-S9).

The largest conventional VIF values were 7.08 for pre-diagnosis smoking duration and 6.03 for years since diagnosis; no VIF exceeded 10. Leave-one-covariate-out estimates were materially unchanged, with heart-disease HRs ranging from 0.584 to 0.586 (Table S5).

The exposure-specific proportional hazards tests were nonsignificant for all-cause, heart-disease, and cancer mortality (P=0.065, 0.569, and 0.264). The global test was significant for all-cause mortality (P=0.027) but not for heart-disease or cancer mortality (P=0.174 and 0.635) (Table S13).

Among variables included in the propensity-score model, the maximum absolute standardized mean difference decreased from 0.720 under survey weights alone to 0.003 after survey-by-overlap weighting (Table S11).

Table S1. Construction of the diagnosis-anchored analytic sample from pooled NHANES 1999-2018 linked-mortality data, United States

Step	Retained n	Excluded from previous step
All NHANES participants, 1999–2018	101316	
Age ≥40 years	36252	65064
Any prespecified cardiopulmonary disease	7100	29152
Valid age at first cardiopulmonary diagnosis	6964	136
Ever smoker who started by diagnosis	3972	2992
Classified as smoking at diagnosis	2388	1584
Exposure classifiable: post-diagnostic quitter or persistent smoker	2388	0
Eligible for mortality linkage with positive interview follow-up	2357	31
Valid 20-year interview weight and survey design variables	2319	38
Final primary analytic domain	2319	0

Counts are unweighted. The final analytic sample included 975 post-diagnostic quitters and 1344 persistent smokers.

Table S2. Continuous baseline variables in the analytic sample, pooled NHANES 1999-2018 linked-mortality data, United States (n=2319)

Variable	Group	Nonmissing n	Survey-weighted mean (SD)	Unweighted median (IQR)
Age, years	Persistent smoker	1344	57.19 ± 10.26	59.00 (51.00–67.00)
Age, years	Post-diagnostic quitter	975	64.41 ± 11.01	68.00 (61.00–76.00)
NHANES survey midyear	Persistent smoker	1344	2008.95 ± 5.81	2009.50 (2003.50–2013.50)
NHANES survey midyear	Post-diagnostic quitter	975	2009.08 ± 5.93	2007.50 (2003.50–2013.50)
Years since first cardiopulmonary diagnosis	Persistent smoker	1344	12.22 ± 10.50	10.00 (4.00–19.00)
Years since first cardiopulmonary diagnosis	Post-diagnostic quitter	975	20.80 ± 13.19	17.00 (10.00–30.00)
Regular-smoking duration before diagnosis, years	Persistent smoker	1344	27.90 ± 13.52	30.00 (20.00–40.00)
Regular-smoking duration before diagnosis, years	Post-diagnostic quitter	975	26.58 ± 14.84	30.00 (18.00–41.00)
Family poverty-to-income ratio	Persistent smoker	1215	2.18 ± 1.51	1.29 (0.82–2.42)
Family poverty-to-income ratio	Post-diagnostic quitter	897	2.68 ± 1.56	1.76 (1.09–3.22)

Variable	Group	Nonmissing n	Survey-weighted mean (SD)	Unweighted median (IQR)
Cigarettes per day proxy	Persistent smoker	1335	17.88 ± 13.35	15.00 (7.00–20.00)
Cigarettes per day proxy	Post-diagnostic quitter	905	25.87 ± 18.02	20.00 (10.00–40.00)
Pack-years proxy	Persistent smoker	1334	35.47 ± 27.45	28.50 (13.31–46.25)
Pack-years proxy	Post-diagnostic quitter	905	46.42 ± 37.25	39.00 (18.50–65.00)
Body mass index, kg/m2	Persistent smoker	1255	28.44 ± 7.01	27.42 (23.30–32.81)
Body mass index, kg/m2	Post-diagnostic quitter	876	30.77 ± 7.10	29.51 (25.62–33.99)

SD, standard deviation; IQR, interquartile range.

Table S3. Categorical baseline variables in the analytic sample, pooled NHANES 1999-2018 linked-mortality data, United States (n=2319)

Variable	Level	Group	Unweighted n (survey-weighted %)
Sex	Male	Persistent smoker	690 (44.7%)
Sex	Male	Post-diagnostic quitter	600 (54.5%)
Sex	Female	Persistent smoker	654 (55.3%)
Sex	Female	Post-diagnostic quitter	375 (45.5%)
Race and ethnicity	Non-Hispanic White	Persistent smoker	775 (75.9%)
Race and ethnicity	Non-Hispanic White	Post-diagnostic quitter	620 (83.3%)
Race and ethnicity	Hispanic	Persistent smoker	159 (4.8%)
Race and ethnicity	Hispanic	Post-diagnostic quitter	136 (4.8%)
Race and ethnicity	Non-Hispanic Black	Persistent smoker	321 (11.8%)
Race and ethnicity	Non-Hispanic Black	Post-diagnostic quitter	170 (7.6%)
Race and ethnicity	Other/Multiracial	Persistent smoker	89 (7.5%)
Race and ethnicity	Other/Multiracial	Post-diagnostic quitter	49 (4.3%)
Education	High school or less	Persistent smoker	907 (63.7%)
Education	High school or less	Post-diagnostic quitter	609 (57.3%)
Education	Some college or above	Persistent smoker	436 (36.3%)
Education	Some college or above	Post-diagnostic quitter	364 (42.5%)
Education	Unknown	Persistent smoker	1 (0.0%)
Education	Unknown	Post-diagnostic quitter	2 (0.1%)
Cardiopulmonary disease phenotype	Cardiovascular disease only	Persistent smoker	534 (34.6%)
Cardiopulmonary disease phenotype	Cardiovascular disease only	Post-diagnostic quitter	429 (38.1%)
Cardiopulmonary disease phenotype	Chronic lung disease only	Persistent smoker	595 (50.0%)
Cardiopulmonary disease phenotype	Chronic lung disease only	Post-diagnostic quitter	352 (41.7%)
Cardiopulmonary disease phenotype	Both cardiovascular and lung disease	Persistent smoker	214 (15.4%)
Cardiopulmonary disease phenotype	Both cardiovascular and lung disease	Post-diagnostic quitter	194 (20.2%)
Cardiopulmonary disease phenotype	Other	Persistent smoker	1 (0.0%)
Cardiopulmonary disease phenotype	Other	Post-diagnostic quitter	0 (0.0%)
Diabetes	No	Persistent smoker	1011 (80.4%)
Diabetes	No	Post-diagnostic quitter	642 (69.0%)
Diabetes	Yes	Persistent smoker	295 (17.5%)
Diabetes	Yes	Post-diagnostic quitter	302 (28.2%)
Diabetes	Unknown	Persistent smoker	38 (2.1%)
Diabetes	Unknown	Post-diagnostic quitter	31 (2.9%)
Hypertension	No	Persistent smoker	527 (45.5%)

Variable	Level	Group	Unweighted n (survey-weighted %)
Hypertension	No	Post-diagnostic quitter	346 (40.7%)
Hypertension	Yes	Persistent smoker	816 (54.4%)
Hypertension	Yes	Post-diagnostic quitter	627 (59.1%)
Hypertension	Unknown	Persistent smoker	1 (0.1%)
Hypertension	Unknown	Post-diagnostic quitter	2 (0.2%)
Age group	40–64 years	Persistent smoker	909 (75.5%)
Age group	40–64 years	Post-diagnostic quitter	381 (49.4%)
Age group	≥65 years	Persistent smoker	435 (24.5%)
Age group	≥65 years	Post-diagnostic quitter	594 (50.6%)

Percentages incorporate the pooled NHANES interview weights.

Table S4. Missingness in the primary analytic sample, pooled NHANES 1999-2018 linked-mortality data, United States (n=2319)

Variable	Total n	Missing n	Missing (%)
Family poverty-to-income ratio	2319	207	8.93
Body mass index	2319	188	8.11
Pack-years proxy	2319	80	3.45
Cigarettes per day proxy	2319	79	3.41
Age	2319	0	0.00
survey_midyear	2319	0	0.00
Years since diagnosis	2319	0	0.00
Pre-diagnosis smoking duration	2319	0	0.00
Sex	2319	0	0.00
Race and ethnicity	2319	0	0.00
Education	2319	0	0.00
Disease phenotype	2319	0	0.00
diabetes_f	2319	0	0.00
hypertension_f	2319	0	0.00
age_group_f	2319	0	0.00
Follow-up time	2319	0	0.00
All-cause death	2319	0	0.00
Heart-disease death	2319	0	0.00
Cancer death	2319	0	0.00
wt_int_20y	2319	0	0.00
sdmvpsu	2319	0	0.00
sdmvstra	2319	0	0.00

Complete-case analysis was used within each model. Variables in the fully adjusted core model had no missing observations.

Table S5. Multicollinearity diagnostics and leave-one-covariate-out sensitivity analyses for the fully adjusted core models

Part A. VIF/GVIF diagnostics

Term	Raw GVIF	df	GVIF^(1/[2 x df])	Equivalent VIF (1 df)	Interpretation

Term	Raw GVIF	df	GVIF^(1/[2 x df])	Equivalent VIF (1 df)	Interpretation
Post-diagnostic quitting	1.216	1	1.103	1.216	No meaningful multicollinearity
Age, per 10 years	4.781	1	2.186	4.781	No meaningful multicollinearity
Sex	1.14	1	1.068	1.14	No meaningful multicollinearity
Race and ethnicity	1.102	3	1.016		No meaningful multicollinearity
Education	1.038	2	1.009		No meaningful multicollinearity
Disease phenotype	1.189	3	1.029		No meaningful multicollinearity
Years since diagnosis, per 5 years	6.029	1	2.455	6.029	Moderate multicollinearity
Pre-diagnosis smoking duration, per 10 years	7.077	1	2.66	7.077	Moderate multicollinearity
Centered 2-year survey cycle	1.036	1	1.018	1.036	No meaningful multicollinearity

VIF values of 5-10 were considered moderate and values above 10 potentially serious. Equivalent standard VIF is shown only for one-degree-of-freedom terms.

Part B. Leave-one-covariate-out models

Outcome	Model	n	Deaths	HR (95% CI)	P value	Absolute change in log(HR)	Material change
All-cause mortality	Full Model 2	2319	999	0.90 (0.74–1.09)	0.276	0.000	No
All-cause mortality	Model 2 without years since diagnosis	2319	999	0.90 (0.74–1.10)	0.298	0.005	No
All-cause mortality	Model 2 without pre-diagnosis smoking duration	2319	999	0.89 (0.73–1.09)	0.249	0.007	No
Heart-disease mortality	Full Model 2	2319	272	0.59 (0.42–0.82)	0.001	0.000	No
Heart-disease mortality	Model 2 without years since diagnosis	2319	272	0.58 (0.42–0.81)	0.001	0.004	No
Heart-disease mortality	Model 2 without pre-diagnosis smoking duration	2319	272	0.59 (0.42–0.82)	0.002	0.001	No
Cancer mortality	Full Model 2	2319	244	0.72 (0.49–1.06)	0.1	0.000	No
Cancer mortality	Model 2 without years since diagnosis	2319	244	0.74 (0.51–1.09)	0.133	0.030	No
Cancer mortality	Model 2 without pre-diagnosis smoking duration	2319	244	0.71 (0.48–1.04)	0.081	0.018	No

Hazard ratios compare post-diagnostic quitters with persistent smokers. A material change flag was prespecified in the analysis code; none was observed.

Table S6. Mortality outcome counts and NHANES-cycle availability

Outcome	NHANES cycles	Deaths	Analytic n
All-cause mortality	1999–2018	999	2319
Heart-disease mortality (001)	1999–2018	272	2319
Cancer mortality (002)	1999–2018	244	2319
Cardiovascular composite mortality (001+005)	1999–2014	283	1765

Outcome	NHANES cycles	Deaths	Analytic n
Chronic lower respiratory disease mortality (003)	1999–2014	152	1765

Cause-of-death category codes are from the National Center for Health Statistics 2019 public-use Linked Mortality Files.

Table S7. Distribution of absolute discrepancies between directly reported and derived cessation ages among post-diagnostic quitters

Measure	Value
Quitters with both direct and derived cessation ages	772
Mean absolute discrepancy, years	1.00
Standard deviation, years	2.91
Median absolute discrepancy, years	0
25th percentile, years	0
75th percentile, years	1
90th percentile, years	3
Maximum discrepancy, years	30.17
Discrepancy >1 year, n	120
Discrepancy >2 years, n	83
Discrepancy >5 years, n	40

The discrepancy was calculated only for quitters with both cessation-age sources.

Table S8. Analytic sample counts for quit-age reconstruction sensitivity analyses.

Analysis	Excluded quitters	Retained n	Retained quitters	Persistent smokers	All-cause deaths	Heart-disease deaths	Cancer deaths
Exclude direct-derived quit-age discrepancy >1 years	120	2199	855	1344	917	249	232
Exclude direct-derived quit-age discrepancy >2 years	83	2236	892	1344	941	252	239
Exclude direct-derived quit-age discrepancy >5 years	40	2279	935	1344	967	261	240
Quitters restricted to directly reported SMD055 quit age		2117	773	1344	936	258	233

Participants with only one usable cessation-age source were retained in discrepancy-threshold analyses unless excluded by the specified rule.

Table S9. Quit-age source availability and direct-derived discrepancies by NHANES cycle

Cycle	Quitters	Direct age	Derived age	Both sources	>1 year	>2 years	>5 years	Median discrepancy
1999-2000	77	67	77	67	4	1	0	0
2001-2002	107	95	107	95	6	5	2	0
2003-2004	109	101	109	101	14	11	3	0
2005-2006	90	84	90	84	5	2	2	0
2007-2008	106	100	106	100	22	13	6	0
2009-2010	95	81	95	81	12	10	3	0
2011-2012	64	50	64	50	12	6	5	0

Cycle	Quitters	Direct age	Derived age	Both sources	>1 year	>2 years	>5 years	Median discrepancy
2013-2014	96	88	95	87	21	16	8	0
2015-2016	116	107	116	107	24	19	11	0
2017-2018	115	0	115	0	0	0	0	

Counts are unweighted.

Table S10. Complete survey-weighted Cox model results and quality-control flags

Analysis	Outcome	n	Deaths	HR (95% CI)	P value	Status	Narrow-CI flag	Extreme-P flag
Primary 1999–2018 / M0_unadjusted	All-cause mortality	2319	999	1.53 (1.27–1.85)	<0.001	ok	0	0
Primary 1999–2018 / M1_demographic	All-cause mortality	2319	999	0.82 (0.68–0.99)	0.043	ok	0	0
Primary 1999–2018 / M2_core	All-cause mortality	2319	999	0.90 (0.74–1.09)	0.276	ok	0	0
Primary 1999–2018 / M0_unadjusted	Heart-disease mortality	2319	272	1.14 (0.84–1.56)	0.398	ok	0	0
Primary 1999–2018 / M1_demographic	Heart-disease mortality	2319	272	0.55 (0.39–0.76)	<0.001	ok	0	0
Primary 1999–2018 / M2_core	Heart-disease mortality	2319	272	0.59 (0.42–0.82)	0.001	ok	0	0
Primary 1999–2018 / M0_unadjusted	Cancer mortality	2319	244	1.21 (0.85–1.72)	0.301	ok	0	0
Primary 1999–2018 / M1_demographic	Cancer mortality	2319	244	0.72 (0.50–1.04)	0.084	ok	0	0
Primary 1999–2018 / M2_core	Cancer mortality	2319	244	0.72 (0.49–1.06)	0.100	ok	0	0
Secondary restricted 1999–2014 / M0_unadjusted	Cardiovascular composite mortality (heart + cerebrovascular)	1765	283	1.17 (0.86–1.61)	0.319	ok	0	0
Secondary restricted 1999–2014 / M1_demographic	Cardiovascular composite mortality (heart + cerebrovascular)	1765	283	0.56 (0.40–0.78)	<0.001	ok	0	0
Secondary restricted 1999–2014 / M2_core	Cardiovascular composite mortality (heart + cerebrovascular)	1765	283	0.59 (0.42–0.83)	0.003	ok	0	0
Secondary restricted 1999–2014 / M0_unadjusted	Chronic lower respiratory disease mortality	1765	152	1.60 (1.09–2.35)	0.016	ok	0	0
Secondary restricted 1999–2014 / M1_demographic	Chronic lower respiratory disease mortality	1765	152	0.79 (0.53–1.16)	0.229	ok	0	0
Secondary restricted 1999–2014 / M2_core	Chronic lower respiratory disease mortality	1765	152	1.18 (0.76–1.84)	0.461	ok	0	0
Primary 1999–2018 without calendar-cycle adjustment	All-cause mortality	2319	999	0.90 (0.74–1.09)	0.280	ok	0	0
Primary 1999–2018 without calendar-cycle adjustment	Heart-disease mortality	2319	272	0.60 (0.42–0.83)	0.003	ok	0	0
Restricted 1999–2016 with 18-year combined interview weights	All-cause mortality	2048	971	0.90 (0.74–1.10)	0.310	ok	0	0
Restricted 1999–2016 with 18-year combined interview weights	Heart-disease mortality	2048	264	0.58 (0.41–0.81)	0.001	ok	0	0
Strict exposure: quit age > diagnosis age	All-cause mortality	2093	877	0.99 (0.79–1.24)	0.945	ok	0	0
Strict exposure: quit age > diagnosis age	Heart-disease mortality	2093	235	0.59 (0.41–0.85)	0.005	ok	0	0

Analysis	Outcome	n	Deaths	HR (95% CI)	P value	Status	Narrow-CI flag	Extreme-P flag
MEC-weighted extended complete-case model	All-cause mortality	1943	801	0.95 (0.77–1.18)	0.645	ok	0	0
MEC-weighted extended complete-case model	Heart-disease mortality	1943	212	0.53 (0.36–0.79)	0.002	ok	0	0
Smoking-burden proxy complete-case sensitivity	All-cause mortality	2239	951	0.87 (0.70–1.07)	0.184	ok	0	0
Smoking-burden proxy complete-case sensitivity	Heart-disease mortality	2239	260	0.56 (0.40–0.80)	0.001	ok	0	0
Restricted to participants with cardiovascular disease	All-cause mortality	1371	658	0.83 (0.66–1.05)	0.122	ok	0	0
Restricted to participants with chronic lung disease	All-cause mortality	1356	570	1.03 (0.81–1.33)	0.790	ok	0	0
Restricted to participants with cardiovascular disease	Heart-disease mortality	1371	213	0.60 (0.42–0.86)	0.005	ok	0	0
Restricted to participants with chronic lung disease	Heart-disease mortality	1356	136	0.62 (0.40–0.96)	0.033	ok	0	0
Landmark at 12 months / M2_core	All-cause mortality	2196	876	0.86 (0.70–1.07)	0.174	ok	0	0
Landmark at 12 months / M2_core	Heart-disease mortality	2196	233	0.57 (0.39–0.82)	0.003	ok	0	0
Landmark at 24 months / M2_core	All-cause mortality	1958	768	0.85 (0.68–1.06)	0.149	ok	0	0
Landmark at 24 months / M2_core	Heart-disease mortality	1958	205	0.62 (0.42–0.93)	0.020	ok	0	0
Landmark at 36 months / M2_core	All-cause mortality	1742	662	0.80 (0.64–1.01)	0.064	ok	0	0
Landmark at 36 months / M2_core	Heart-disease mortality	1742	175	0.60 (0.40–0.92)	0.019	ok	0	0
Survey × overlap-weighted supportive analysis	All-cause mortality	2319	999	0.95 (0.78–1.16)	0.633	ok	0	0
Survey × overlap-weighted supportive analysis	Heart-disease mortality	2319	272	0.63 (0.45–0.86)	0.004	ok	0	0
Survey × overlap-weighted doubly adjusted sensitivity	All-cause mortality	2319	999	0.90 (0.74–1.09)	0.270	ok	0	0
Survey × overlap-weighted doubly adjusted sensitivity	Heart-disease mortality	2319	272	0.58 (0.42–0.81)	0.001	ok	0	0
Subgroup: Age group = 40–64 years: Age group - 40–64 years	Heart-disease mortality	1290	94	0.34 (0.17–0.66)	0.001	ok	0	0
Subgroup: Age group = ≥65 years: Age group - ≥65 years	Heart-disease mortality	1029	178	0.76 (0.52–1.11)	0.156	ok	0	0
Subgroup: Sex = Male: Sex - Male	Heart-disease mortality	1290	171	0.48 (0.33–0.71)	<0.001	ok	0	0
Subgroup: Sex = Female: Sex - Female	Heart-disease mortality	1029	101	0.77 (0.49–1.22)	0.266	ok	0	0
Subgroup: Disease phenotype = Cardiovascular disease only: Disease phenotype - Cardiovascular disease only	Heart-disease mortality	963	136	0.50 (0.33–0.76)	0.001	ok	0	0
Subgroup: Disease phenotype = Chronic lung disease only: Disease phenotype - Chronic lung disease only	Heart-disease mortality	947	59	0.36 (0.18–0.73)	0.004	ok	0	0

Analysis	Outcome	n	Deaths	HR (95% CI)	P value	Status	Narrow-CI flag	Extreme-P flag
Subgroup: Disease phenotype = Both cardiovascular and lung disease: Disease phenotype - Both cardiovascular and lung disease	Heart-disease mortality	408	77	1.01 (0.55–1.86)	0.973	ok	0	0
Subgroup: Disease phenotype = Other: Disease phenotype - Other	Heart-disease mortality	1	0	Not estimable		not_run	0	0
Quit-age discrepancy exclusion >1 years	All-cause mortality	2199	917	0.84 (0.68–1.04)	0.110	ok	0	0
Quit-age discrepancy exclusion >1 years	Heart-disease mortality	2199	249	0.52 (0.37–0.73)	<0.001	ok	0	0
Quit-age discrepancy exclusion >1 years	Cancer mortality	2199	232	0.73 (0.49–1.07)	0.106	ok	0	0
Quit-age discrepancy exclusion >2 years	All-cause mortality	2236	941	0.85 (0.69–1.05)	0.124	ok	0	0
Quit-age discrepancy exclusion >2 years	Heart-disease mortality	2236	252	0.53 (0.38–0.74)	<0.001	ok	0	0
Quit-age discrepancy exclusion >2 years	Cancer mortality	2236	239	0.74 (0.50–1.09)	0.130	ok	0	0
Quit-age discrepancy exclusion >5 years	All-cause mortality	2279	967	0.87 (0.72–1.07)	0.193	ok	0	0
Quit-age discrepancy exclusion >5 years	Heart-disease mortality	2279	261	0.56 (0.40–0.78)	<0.001	ok	0	0
Quit-age discrepancy exclusion >5 years	Cancer mortality	2279	240	0.72 (0.49–1.07)	0.103	ok	0	0
Quitters restricted to directly reported SMD055 quit age	All-cause mortality	2117	936	0.87 (0.70–1.08)	0.203	ok	0	0
Quitters restricted to directly reported SMD055 quit age	Heart-disease mortality	2117	258	0.58 (0.41–0.81)	0.002	ok	0	0
Quitters restricted to directly reported SMD055 quit age	Cancer mortality	2117	233	0.72 (0.48–1.09)	0.124	ok	0	0

Hazard ratios compare post-diagnostic quitters with persistent smokers. The residual Other/uncategorized disease subgroup was not estimable because it contained one participant, no quitter, and no event.

Table S11. Covariate balance before and after multiplication of survey and overlap weights

Covariate	Survey weight only: absolute ASD	Survey x overlap weight: absolute ASD
Years since first diagnosis	0.720	0.000
Age, years	0.679	0.000
Sex: Male	0.196	0.000
Sex: Female	0.196	0.000
Race/ethnicity: Non-Hispanic White	0.184	0.000
Cardiopulmonary disease phenotype: Chronic lung disease only	0.165	0.000
Race/ethnicity: Non-Hispanic Black	0.143	0.000
Race/ethnicity: Other/Multiracial	0.135	0.000
Educational attainment: High school or less	0.130	0.000
Educational attainment: Some college or above	0.128	0.000
Cardiopulmonary disease phenotype: Both cardiovascular and lung disease	0.125	0.000
Pre-diagnosis smoking duration, years	0.093	0.000

Covariate	Survey weight only: absolute ASD	Survey x overlap weight: absolute ASD
Cardiopulmonary disease phenotype: Cardiovascular disease only	0.072	0.000
Educational attainment: Unknown	0.040	0.000
Survey calendar year	0.023	0.000
Cardiopulmonary disease phenotype: Other	0.020	0.003
Race/ethnicity: Hispanic	0.001	0.000

Only variables included in the propensity-score model are shown. An absolute ASD below 0.10 indicated acceptable balance. ASD, absolute standardized difference.

Table S12. Design-based interaction tests for exploratory heart-disease mortality subgroup analyses

Subgroup variable	n	Heart-disease deaths	P for interaction	Status
Age group	2319	272	0.101	ok
Sex	2319	272	0.024	ok
Disease phenotype	2318	272	0.129	ok

The Bonferroni threshold for the three interaction tests was P<0.0167.

Table S13. Corrected term-level proportional hazards tests for the fully adjusted mortality models

Outcome	n	Deaths	Term	Chi-square	df	P value
All-cause mortality	2319	999	Post-diagnostic quitting	3.404	1	0.065
All-cause mortality	2319	999	Age, per 10 years	2.455	1	0.117
All-cause mortality	2319	999	Sex	1.734	1	0.188
All-cause mortality	2319	999	Race and ethnicity	7.505	3	0.057
All-cause mortality	2319	999	Education	0.215	2	0.898
All-cause mortality	2319	999	Disease phenotype	4.174	3	0.243
All-cause mortality	2319	999	Years since diagnosis, per 5 years	0.087	1	0.768
All-cause mortality	2319	999	Pre-diagnosis smoking duration, per 10 years	0.703	1	0.402
All-cause mortality	2319	999	Centered 2-year survey cycle	1.779	1	0.182
All-cause mortality	2319	999	GLOBAL	25.897	14	0.027
Heart-disease mortality	2319	272	Post-diagnostic quitting	0.324	1	0.569
Heart-disease mortality	2319	272	Age, per 10 years	7.682	1	0.006
Heart-disease mortality	2319	272	Sex	0.167	1	0.683
Heart-disease mortality	2319	272	Race and ethnicity	4.300	3	0.231
Heart-disease mortality	2319	272	Education	0.003	2	0.999
Heart-disease mortality	2319	272	Disease phenotype	0.646	3	0.886
Heart-disease mortality	2319	272	Years since diagnosis, per 5 years	2.189	1	0.139
Heart-disease mortality	2319	272	Pre-diagnosis smoking duration, per 10 years	0.123	1	0.726
Heart-disease mortality	2319	272	Centered 2-year survey cycle	5.966	1	0.015
Heart-disease mortality	2319	272	GLOBAL	18.779	14	0.174
Cancer mortality	2319	244	Post-diagnostic quitting	1.247	1	0.264

Outcome	n	Deaths	Term	Chi-square	df	P value
Cancer mortality	2319	244	Age, per 10 years	0.799	1	0.371
Cancer mortality	2319	244	Sex	0.889	1	0.346
Cancer mortality	2319	244	Race and ethnicity	1.350	3	0.717
Cancer mortality	2319	244	Education	0.986	2	0.611
Cancer mortality	2319	244	Disease phenotype	5.620	3	0.132
Cancer mortality	2319	244	Years since diagnosis, per 5 years	0.071	1	0.79
Cancer mortality	2319	244	Pre-diagnosis smoking duration, per 10 years	0.094	1	0.76
Cancer mortality	2319	244	Centered 2-year survey cycle	0.032	1	0.859
Cancer mortality	2319	244	GLOBAL	11.647	14	0.635

P values were obtained from scaled Schoenfeld residual tests. A P value below 0.05 indicates statistical evidence against proportional hazards. Term-level tests preserved the degrees of freedom of multilevel categorical variables.

Figure source data

Post-diagnostic smoking cessation and heart-disease mortality among adults smoking at cardiopulmonary disease diagnosis: an NHANES linked-mortality analysis

This document provides the numerical data underlying Figures 1-4. Values are reproduced from the final analysis outputs used to generate the submitted TIFF files. Internal programming and model-object fields not displayed in the figures are omitted for readability.

Figure 1A source data. Participant-flow nodes

Order	Flowchart node	Retained n
1	All NHANES participants, 1999–2018	101316
2	Age ≥40 years	36252
3	Any prespecified cardiopulmonary disease	7100
4	Valid age at first cardiopulmonary diagnosis	6964
5	Ever smoker who started by diagnosis	3972
6	Classified as smoking at diagnosis	2388
7	Exposure classifiable: post-diagnostic quitter or persistent smoker	2388
8	Eligible for mortality linkage with positive interview follow-up	2357
9	Valid 20-year interview weight and survey design variables	2319

Figure 1A source data. Displayed exclusions

Reason	Excluded n	Displayed label
Age <40 years	65064	Excluded: 65,064<br>Age <40 years
No selected cardiopulmonary disease	29152	Excluded: 29,152<br>No selected cardiopulmonary<br>disease
Missing/invalid first diagnosis age	136	Excluded: 136<br>Missing/invalid first<br>diagnosis age
Never smoker or smoking began after diagnosis	2992	Excluded: 2,992<br>Never smoker or smoking began<br>after diagnosis
Not classifiable as smoking at diagnosis	1584	Excluded: 1,584<br>Not classifiable as smoking<br>at diagnosis
Mortality-linkage ineligible or no positive follow-up	31	Excluded: 31<br>Mortality-linkage ineligible<br>or no positive follow-up
Invalid combined interview weight/design variables	38	Excluded: 38<br>Invalid combined interview<br>weight/design variables

Zero-count exclusion annotations were not displayed in the final flowchart.

Figure 1B source data. Survey-cycle composition by exposure group

NHANES cycle	Exposure group	Unweighted n
1999-2000	Post-diagnostic quitter	77
1999-2000	Persistent smoker	108
2001-2002	Post-diagnostic quitter	107
2001-2002	Persistent smoker	105
2003-2004	Post-diagnostic quitter	109
2003-2004	Persistent smoker	124
2005-2006	Post-diagnostic quitter	90
2005-2006	Persistent smoker	126
2007-2008	Post-diagnostic quitter	106
2007-2008	Persistent smoker	162
2009-2010	Post-diagnostic quitter	95
2009-2010	Persistent smoker	145
2011-2012	Post-diagnostic quitter	64
2011-2012	Persistent smoker	121
2013-2014	Post-diagnostic quitter	96
2013-2014	Persistent smoker	130
2015-2016	Post-diagnostic quitter	116
2015-2016	Persistent smoker	167
2017-2018	Post-diagnostic quitter	115
2017-2018	Persistent smoker	156

Figure 2A source data. Group counts for the survey-weighted unadjusted descriptive Kaplan-Meier curves for heart-disease-free survival

Exposure group	Unweighted n
Persistent smoker	1344
Post-diagnostic quitter	975

Figure 2A source data. Survey-weighted unadjusted descriptive Kaplan-Meier coordinates for heart-disease-free survival

Exposure group	Follow-up time, years	Heart-disease-free survival probability
Persistent smoker	0.000000	1.00000000
Persistent smoker	0.083333	0.99937975
Persistent smoker	0.166667	0.99862702
Persistent smoker	0.250000	0.99833436
Persistent smoker	0.333333	0.99808864
Persistent smoker	0.416667	0.99727772
Persistent smoker	0.500000	0.99686595
Persistent smoker	0.583333	0.99545196
Persistent smoker	0.666667	0.99425418
Persistent smoker	0.750000	0.99425418
Persistent smoker	0.833333	0.99292557
Persistent smoker	0.916667	0.99239966
Persistent smoker	1.000000	0.98891348

Exposure group	Follow-up time, years	Heart-disease-free survival probability
Persistent smoker	1.083333	0.98891348
Persistent smoker	1.166667	0.98793396
Persistent smoker	1.250000	0.98793396
Persistent smoker	1.333333	0.98793396
Persistent smoker	1.416667	0.98793396
Persistent smoker	1.500000	0.98456647
Persistent smoker	1.583333	0.98344395
Persistent smoker	1.666667	0.98146489
Persistent smoker	1.750000	0.98111557
Persistent smoker	1.833333	0.98044457
Persistent smoker	1.916667	0.97778119
Persistent smoker	2.000000	0.97526227
Persistent smoker	2.083333	0.97521280
Persistent smoker	2.166667	0.97521280
Persistent smoker	2.250000	0.97497668
Persistent smoker	2.333333	0.97497668
Persistent smoker	2.416667	0.97461214
Persistent smoker	2.500000	0.97058146
Persistent smoker	2.583333	0.96967857
Persistent smoker	2.666667	0.96933465
Persistent smoker	2.750000	0.96768840
Persistent smoker	2.833333	0.96720039
Persistent smoker	2.916667	0.96598628
Persistent smoker	3.000000	0.96598628
Persistent smoker	3.083333	0.96565962
Persistent smoker	3.166667	0.96505767
Persistent smoker	3.250000	0.96245049
Persistent smoker	3.333333	0.96129712
Persistent smoker	3.416667	0.96056036
Persistent smoker	3.500000	0.95733041
Persistent smoker	3.583333	0.95733041
Persistent smoker	3.666667	0.95119434
Persistent smoker	3.750000	0.94999565
Persistent smoker	3.833333	0.94938914
Persistent smoker	3.916667	0.94784355
Persistent smoker	4.000000	0.94712849
Persistent smoker	4.083333	0.94712849
Persistent smoker	4.166667	0.94712849
Persistent smoker	4.250000	0.94712849
Persistent smoker	4.333333	0.94649117
Persistent smoker	4.416667	0.94596349
Persistent smoker	4.500000	0.94472203
Persistent smoker	4.583333	0.94407406
Persistent smoker	4.666667	0.94348923
Persistent smoker	4.750000	0.94333199
Persistent smoker	4.833333	0.94333199
Persistent smoker	4.916667	0.93799768

Exposure group	Follow-up time, years	Heart-disease-free survival probability
Persistent smoker	5.000000	0.93480012
Persistent smoker	5.083333	0.93425250
Persistent smoker	5.166667	0.93415608
Persistent smoker	5.250000	0.93084887
Persistent smoker	5.333333	0.93043503
Persistent smoker	5.416667	0.93043503
Persistent smoker	5.500000	0.93043503
Persistent smoker	5.583333	0.93043503
Persistent smoker	5.666667	0.93043503
Persistent smoker	5.750000	0.93043503
Persistent smoker	5.833333	0.92953860
Persistent smoker	5.916667	0.92953860
Persistent smoker	6.000000	0.92953860
Persistent smoker	6.083333	0.92953860
Persistent smoker	6.166667	0.92953860
Persistent smoker	6.250000	0.92953860
Persistent smoker	6.333333	0.92945085
Persistent smoker	6.500000	0.92643656
Persistent smoker	6.583333	0.92419715
Persistent smoker	6.666667	0.92395439
Persistent smoker	6.750000	0.92395439
Persistent smoker	6.833333	0.92395439
Persistent smoker	6.916667	0.92395439
Persistent smoker	7.000000	0.92292833
Persistent smoker	7.083333	0.91978499
Persistent smoker	7.166667	0.91978499
Persistent smoker	7.250000	0.91942213
Persistent smoker	7.333333	0.91823715
Persistent smoker	7.416667	0.91823715
Persistent smoker	7.500000	0.91823715
Persistent smoker	7.583333	0.91823715
Persistent smoker	7.666667	0.91809526
Persistent smoker	7.750000	0.91738927
Persistent smoker	7.833333	0.91326770
Persistent smoker	7.916667	0.91281948
Persistent smoker	8.000000	0.91281948
Persistent smoker	8.083333	0.91187256
Persistent smoker	8.166667	0.91187256
Persistent smoker	8.333333	0.91056137
Persistent smoker	8.416667	0.91014230
Persistent smoker	8.500000	0.91014230
Persistent smoker	8.583333	0.91014230
Persistent smoker	8.666667	0.90746628
Persistent smoker	8.750000	0.90675037
Persistent smoker	8.833333	0.90675037
Persistent smoker	8.916667	0.90675037
Persistent smoker	9.000000	0.90645521

Exposure group	Follow-up time, years	Heart-disease-free survival probability
Persistent smoker	9.083333	0.90617073
Persistent smoker	9.166667	0.90617073
Persistent smoker	9.250000	0.90441560
Persistent smoker	9.333333	0.90428743
Persistent smoker	9.416667	0.90298545
Persistent smoker	9.500000	0.90141203
Persistent smoker	9.583333	0.90141203
Persistent smoker	9.666667	0.89991112
Persistent smoker	9.750000	0.89921570
Persistent smoker	9.833333	0.89891065
Persistent smoker	9.916667	0.89891065
Persistent smoker	10.000000	0.89618262
Persistent smoker	10.083333	0.89596236
Persistent smoker	10.166667	0.89415839
Persistent smoker	10.250000	0.89415839
Persistent smoker	10.333333	0.89415839
Persistent smoker	10.416667	0.89415839
Persistent smoker	10.500000	0.89415839
Persistent smoker	10.583333	0.89415839
Persistent smoker	10.666667	0.89272042
Persistent smoker	10.750000	0.89272042
Persistent smoker	10.833333	0.89272042
Persistent smoker	10.916667	0.89272042
Persistent smoker	11.000000	0.89272042
Persistent smoker	11.083333	0.89272042
Persistent smoker	11.166667	0.89272042
Persistent smoker	11.250000	0.89272042
Persistent smoker	11.333333	0.88983727
Persistent smoker	11.416667	0.88983727
Persistent smoker	11.500000	0.88731428
Persistent smoker	11.583333	0.88731428
Persistent smoker	11.666667	0.88731428
Persistent smoker	11.750000	0.88731428
Persistent smoker	11.833333	0.88731428
Persistent smoker	11.916667	0.88731428
Persistent smoker	12.083333	0.88037357
Persistent smoker	12.166667	0.88037357
Persistent smoker	12.250000	0.87814556
Persistent smoker	12.333333	0.87814556
Persistent smoker	12.416667	0.87814556
Persistent smoker	12.500000	0.87553029
Persistent smoker	12.583333	0.87553029
Persistent smoker	12.666667	0.87553029
Persistent smoker	12.750000	0.87553029
Persistent smoker	12.833333	0.87553029
Persistent smoker	12.916667	0.87525297
Persistent smoker	13.000000	0.87525297

Exposure group	Follow-up time, years	Heart-disease-free survival probability
Persistent smoker	13.083333	0.87525297
Persistent smoker	13.166667	0.87326021
Persistent smoker	13.250000	0.87326021
Persistent smoker	13.333333	0.87326021
Persistent smoker	13.416667	0.87326021
Persistent smoker	13.500000	0.87326021
Persistent smoker	13.583333	0.87326021
Persistent smoker	13.750000	0.87326021
Persistent smoker	13.833333	0.87326021
Persistent smoker	13.916667	0.87326021
Persistent smoker	14.000000	0.87326021
Persistent smoker	14.083333	0.87326021
Persistent smoker	14.166667	0.87326021
Persistent smoker	14.250000	0.87326021
Persistent smoker	14.333333	0.87326021
Persistent smoker	14.416667	0.87326021
Persistent smoker	14.500000	0.87326021
Persistent smoker	14.583333	0.86724738
Persistent smoker	14.666667	0.86724738
Persistent smoker	14.750000	0.86724738
Persistent smoker	14.833333	0.86377878
Persistent smoker	14.916667	0.86377878
Persistent smoker	15.000000	0.86377878
Persistent smoker	15.083333	0.84979267
Persistent smoker	15.166667	0.83934761
Persistent smoker	15.250000	0.83934761
Persistent smoker	15.333333	0.83934761
Persistent smoker	15.416667	0.83934761
Persistent smoker	15.500000	0.83934761
Persistent smoker	15.583333	0.83934761
Persistent smoker	15.666667	0.83934761
Persistent smoker	15.750000	0.83934761
Persistent smoker	15.833333	0.83934761
Persistent smoker	15.916667	0.83934761
Persistent smoker	16.083333	0.83934761
Persistent smoker	16.166667	0.83934761
Persistent smoker	16.250000	0.83934761
Persistent smoker	16.333333	0.83934761
Persistent smoker	16.416667	0.83934761
Persistent smoker	16.500000	0.83934761
Persistent smoker	16.583333	0.83934761
Persistent smoker	16.666667	0.83934761
Persistent smoker	16.750000	0.83934761
Persistent smoker	16.916667	0.83694930
Persistent smoker	17.000000	0.83694930
Persistent smoker	17.083333	0.83694930
Persistent smoker	17.166667	0.83694930

Exposure group	Follow-up time, years	Heart-disease-free survival probability
Persistent smoker	17.250000	0.83694930
Persistent smoker	17.500000	0.82083586
Persistent smoker	17.583333	0.82083586
Persistent smoker	17.666667	0.82083586
Persistent smoker	17.750000	0.80667749
Persistent smoker	17.833333	0.80667749
Persistent smoker	18.000000	0.80667749
Persistent smoker	18.083333	0.80667749
Persistent smoker	18.166667	0.80667749
Persistent smoker	18.250000	0.80667749
Persistent smoker	18.333333	0.80667749
Persistent smoker	18.500000	0.80667749
Persistent smoker	18.583333	0.80667749
Persistent smoker	18.666667	0.80667749
Persistent smoker	18.750000	0.80667749
Persistent smoker	19.083333	0.80667749
Persistent smoker	19.166667	0.80667749
Persistent smoker	19.250000	0.80667749
Persistent smoker	19.333333	0.79044550
Persistent smoker	19.416667	0.79044550
Persistent smoker	19.583333	0.79044550
Persistent smoker	19.666667	0.79044550
Persistent smoker	19.750000	0.79044550
Persistent smoker	19.833333	0.79044550
Persistent smoker	19.916667	0.79044550
Persistent smoker	20.000000	0.79044550
Persistent smoker	20.083333	0.79044550
Persistent smoker	20.166667	0.79044550
Persistent smoker	20.250000	0.79044550
Persistent smoker	20.416667	0.79044550
Persistent smoker	20.500000	0.79044550
Persistent smoker	20.583333	0.79044550
Post-diagnostic quitter	0.000000	1.00000000
Post-diagnostic quitter	0.083333	0.99877803
Post-diagnostic quitter	0.166667	0.99877803
Post-diagnostic quitter	0.250000	0.99828947
Post-diagnostic quitter	0.333333	0.99783497
Post-diagnostic quitter	0.416667	0.99456728
Post-diagnostic quitter	0.500000	0.99410622
Post-diagnostic quitter	0.583333	0.99410622
Post-diagnostic quitter	0.666667	0.99351987
Post-diagnostic quitter	0.750000	0.99229778
Post-diagnostic quitter	0.833333	0.99134102
Post-diagnostic quitter	0.916667	0.98971144
Post-diagnostic quitter	1.000000	0.98914485
Post-diagnostic quitter	1.083333	0.98914485
Post-diagnostic quitter	1.166667	0.98660452

Exposure group	Follow-up time, years	Heart-disease-free survival probability
Post-diagnostic quitter	1.250000	0.98660452
Post-diagnostic quitter	1.333333	0.98654201
Post-diagnostic quitter	1.416667	0.98654201
Post-diagnostic quitter	1.500000	0.98542757
Post-diagnostic quitter	1.583333	0.98311157
Post-diagnostic quitter	1.666667	0.98195431
Post-diagnostic quitter	1.750000	0.98195431
Post-diagnostic quitter	1.833333	0.98195431
Post-diagnostic quitter	1.916667	0.98195431
Post-diagnostic quitter	2.000000	0.98121883
Post-diagnostic quitter	2.083333	0.97995327
Post-diagnostic quitter	2.166667	0.97828065
Post-diagnostic quitter	2.250000	0.97799079
Post-diagnostic quitter	2.333333	0.97728168
Post-diagnostic quitter	2.416667	0.97593159
Post-diagnostic quitter	2.500000	0.97325930
Post-diagnostic quitter	2.583333	0.97325930
Post-diagnostic quitter	2.666667	0.97166207
Post-diagnostic quitter	2.750000	0.97051999
Post-diagnostic quitter	2.833333	0.97051999
Post-diagnostic quitter	2.916667	0.97051999
Post-diagnostic quitter	3.000000	0.96971496
Post-diagnostic quitter	3.083333	0.96936098
Post-diagnostic quitter	3.166667	0.96891949
Post-diagnostic quitter	3.250000	0.96891949
Post-diagnostic quitter	3.333333	0.96690818
Post-diagnostic quitter	3.416667	0.96690818
Post-diagnostic quitter	3.500000	0.96524952
Post-diagnostic quitter	3.583333	0.96524952
Post-diagnostic quitter	3.666667	0.96524952
Post-diagnostic quitter	3.750000	0.96294482
Post-diagnostic quitter	3.833333	0.96294482
Post-diagnostic quitter	3.916667	0.95908418
Post-diagnostic quitter	4.000000	0.95593840
Post-diagnostic quitter	4.083333	0.95314325
Post-diagnostic quitter	4.166667	0.95285043
Post-diagnostic quitter	4.250000	0.95285043
Post-diagnostic quitter	4.333333	0.95257611
Post-diagnostic quitter	4.416667	0.95257611
Post-diagnostic quitter	4.500000	0.94895955
Post-diagnostic quitter	4.583333	0.94828864
Post-diagnostic quitter	4.666667	0.94828864
Post-diagnostic quitter	4.750000	0.94828864
Post-diagnostic quitter	4.833333	0.94828864
Post-diagnostic quitter	4.916667	0.94812999
Post-diagnostic quitter	5.000000	0.94656738
Post-diagnostic quitter	5.083333	0.94514405

Exposure group	Follow-up time, years	Heart-disease-free survival probability
Post-diagnostic quitter	5.166667	0.94304090
Post-diagnostic quitter	5.250000	0.94174375
Post-diagnostic quitter	5.333333	0.93930050
Post-diagnostic quitter	5.416667	0.93930050
Post-diagnostic quitter	5.500000	0.93930050
Post-diagnostic quitter	5.583333	0.93706927
Post-diagnostic quitter	5.666667	0.93625153
Post-diagnostic quitter	5.750000	0.93625153
Post-diagnostic quitter	5.833333	0.93531150
Post-diagnostic quitter	5.916667	0.93516203
Post-diagnostic quitter	6.000000	0.93141075
Post-diagnostic quitter	6.083333	0.92603875
Post-diagnostic quitter	6.166667	0.92603875
Post-diagnostic quitter	6.250000	0.92603875
Post-diagnostic quitter	6.333333	0.92487072
Post-diagnostic quitter	6.500000	0.92435849
Post-diagnostic quitter	6.583333	0.92435849
Post-diagnostic quitter	6.666667	0.92173283
Post-diagnostic quitter	6.750000	0.92173283
Post-diagnostic quitter	6.833333	0.91928348
Post-diagnostic quitter	6.916667	0.91928348
Post-diagnostic quitter	7.000000	0.91928348
Post-diagnostic quitter	7.083333	0.91928348
Post-diagnostic quitter	7.166667	0.91822353
Post-diagnostic quitter	7.250000	0.91478077
Post-diagnostic quitter	7.333333	0.91269700
Post-diagnostic quitter	7.416667	0.91231110
Post-diagnostic quitter	7.500000	0.91231110
Post-diagnostic quitter	7.583333	0.90984645
Post-diagnostic quitter	7.666667	0.90984645
Post-diagnostic quitter	7.833333	0.90895993
Post-diagnostic quitter	7.916667	0.90895993
Post-diagnostic quitter	8.000000	0.90895993
Post-diagnostic quitter	8.083333	0.90895993
Post-diagnostic quitter	8.166667	0.90895993
Post-diagnostic quitter	8.333333	0.90870397
Post-diagnostic quitter	8.416667	0.90668362
Post-diagnostic quitter	8.500000	0.90668362
Post-diagnostic quitter	8.583333	0.90515900
Post-diagnostic quitter	8.666667	0.90515900
Post-diagnostic quitter	8.750000	0.90446325
Post-diagnostic quitter	8.833333	0.90254897
Post-diagnostic quitter	8.916667	0.90254897
Post-diagnostic quitter	9.000000	0.90254897
Post-diagnostic quitter	9.083333	0.89585920
Post-diagnostic quitter	9.166667	0.89364278
Post-diagnostic quitter	9.250000	0.89136281

Exposure group	Follow-up time, years	Heart-disease-free survival probability
Post-diagnostic quitter	9.333333	0.88898765
Post-diagnostic quitter	9.416667	0.88898765
Post-diagnostic quitter	9.500000	0.88749010
Post-diagnostic quitter	9.583333	0.88749010
Post-diagnostic quitter	9.666667	0.88749010
Post-diagnostic quitter	9.750000	0.88643025
Post-diagnostic quitter	9.833333	0.88643025
Post-diagnostic quitter	9.916667	0.88005238
Post-diagnostic quitter	10.000000	0.87854740
Post-diagnostic quitter	10.083333	0.87411023
Post-diagnostic quitter	10.166667	0.87411023
Post-diagnostic quitter	10.250000	0.87058315
Post-diagnostic quitter	10.333333	0.86699628
Post-diagnostic quitter	10.416667	0.86699628
Post-diagnostic quitter	10.500000	0.86699628
Post-diagnostic quitter	10.583333	0.86699628
Post-diagnostic quitter	10.666667	0.86699628
Post-diagnostic quitter	10.750000	0.86699628
Post-diagnostic quitter	10.833333	0.86699628
Post-diagnostic quitter	10.916667	0.86699628
Post-diagnostic quitter	11.000000	0.86699628
Post-diagnostic quitter	11.083333	0.86488325
Post-diagnostic quitter	11.166667	0.86367301
Post-diagnostic quitter	11.250000	0.86367301
Post-diagnostic quitter	11.333333	0.86367301
Post-diagnostic quitter	11.416667	0.86367301
Post-diagnostic quitter	11.500000	0.86367301
Post-diagnostic quitter	11.583333	0.86367301
Post-diagnostic quitter	11.666667	0.86367301
Post-diagnostic quitter	11.750000	0.85876480
Post-diagnostic quitter	11.833333	0.85242124
Post-diagnostic quitter	11.916667	0.85242124
Post-diagnostic quitter	12.000000	0.85242124
Post-diagnostic quitter	12.083333	0.85242124
Post-diagnostic quitter	12.166667	0.85242124
Post-diagnostic quitter	12.250000	0.85242124
Post-diagnostic quitter	12.333333	0.85242124
Post-diagnostic quitter	12.416667	0.85242124
Post-diagnostic quitter	12.500000	0.85242124
Post-diagnostic quitter	12.583333	0.85010885
Post-diagnostic quitter	12.666667	0.84292587
Post-diagnostic quitter	12.750000	0.83823319
Post-diagnostic quitter	12.833333	0.83823319
Post-diagnostic quitter	12.916667	0.83823319
Post-diagnostic quitter	13.000000	0.83823319
Post-diagnostic quitter	13.250000	0.83823319
Post-diagnostic quitter	13.333333	0.83823319

Exposure group	Follow-up time, years	Heart-disease-free survival probability
Post-diagnostic quitter	13.416667	0.83823319
Post-diagnostic quitter	13.500000	0.83823319
Post-diagnostic quitter	13.583333	0.83823319
Post-diagnostic quitter	13.750000	0.83823319
Post-diagnostic quitter	13.833333	0.83621281
Post-diagnostic quitter	13.916667	0.83621281
Post-diagnostic quitter	14.000000	0.83621281
Post-diagnostic quitter	14.083333	0.82774899
Post-diagnostic quitter	14.166667	0.82774899
Post-diagnostic quitter	14.250000	0.82774899
Post-diagnostic quitter	14.333333	0.82774899
Post-diagnostic quitter	14.416667	0.82774899
Post-diagnostic quitter	14.583333	0.82442403
Post-diagnostic quitter	14.666667	0.82442403
Post-diagnostic quitter	14.750000	0.82442403
Post-diagnostic quitter	14.833333	0.82442403
Post-diagnostic quitter	14.916667	0.82442403
Post-diagnostic quitter	15.083333	0.81544513
Post-diagnostic quitter	15.166667	0.81544513
Post-diagnostic quitter	15.333333	0.81544513
Post-diagnostic quitter	15.416667	0.81544513
Post-diagnostic quitter	15.666667	0.81544513
Post-diagnostic quitter	15.750000	0.81544513
Post-diagnostic quitter	15.833333	0.81249852
Post-diagnostic quitter	15.916667	0.81249852
Post-diagnostic quitter	16.000000	0.81249852
Post-diagnostic quitter	16.083333	0.81249852
Post-diagnostic quitter	16.166667	0.81249852
Post-diagnostic quitter	16.250000	0.81249852
Post-diagnostic quitter	16.333333	0.80253650
Post-diagnostic quitter	16.583333	0.80253650
Post-diagnostic quitter	16.666667	0.80253650
Post-diagnostic quitter	16.750000	0.80253650
Post-diagnostic quitter	16.833333	0.80253650
Post-diagnostic quitter	16.916667	0.80253650
Post-diagnostic quitter	17.083333	0.78545761
Post-diagnostic quitter	17.166667	0.78545761
Post-diagnostic quitter	17.250000	0.78545761
Post-diagnostic quitter	17.333333	0.78545761
Post-diagnostic quitter	17.416667	0.78545761
Post-diagnostic quitter	17.583333	0.78545761
Post-diagnostic quitter	17.666667	0.78545761
Post-diagnostic quitter	17.750000	0.78545761
Post-diagnostic quitter	17.833333	0.78545761
Post-diagnostic quitter	18.000000	0.78545761
Post-diagnostic quitter	18.083333	0.78545761
Post-diagnostic quitter	18.166667	0.78545761

Exposure group	Follow-up time, years	Heart-disease-free survival probability
Post-diagnostic quitter	18.250000	0.78545761
Post-diagnostic quitter	18.333333	0.78545761
Post-diagnostic quitter	18.416667	0.78545761
Post-diagnostic quitter	18.500000	0.78545761
Post-diagnostic quitter	18.666667	0.78545761
Post-diagnostic quitter	18.833333	0.78545761
Post-diagnostic quitter	18.916667	0.74041877
Post-diagnostic quitter	19.083333	0.74041877
Post-diagnostic quitter	19.166667	0.74041877
Post-diagnostic quitter	19.250000	0.74041877
Post-diagnostic quitter	19.416667	0.74041877
Post-diagnostic quitter	19.583333	0.74041877
Post-diagnostic quitter	19.750000	0.74041877
Post-diagnostic quitter	20.083333	0.74041877
Post-diagnostic quitter	20.250000	0.74041877
Post-diagnostic quitter	20.416667	0.74041877
Post-diagnostic quitter	20.500000	0.74041877
Post-diagnostic quitter	20.583333	0.74041877
Post-diagnostic quitter	20.666667	0.74041877

These Kaplan-Meier curves are unadjusted and descriptive. They should not be interpreted as covariate-adjusted survival estimates.

Figure 2B source data. Full-period mortality models, NHANES 1999-2018

Outcome	Model	n	Deaths	HR	95% CI lower	95% CI upper	P value
All-cause mortality	M0: Unadjusted	2319	999	1.533	1.272	1.846	<0.001
All-cause mortality	M1: Demographic-adjusted	2319	999	0.823	0.682	0.994	0.043
All-cause mortality	M2: Fully adjusted core model	2319	999	0.896	0.735	1.092	0.276
Heart-disease mortality	M0: Unadjusted	2319	272	1.144	0.838	1.561	0.398
Heart-disease mortality	M1: Demographic-adjusted	2319	272	0.545	0.392	0.758	<0.001
Heart-disease mortality	M2: Fully adjusted core model	2319	272	0.586	0.422	0.815	0.001
Cancer mortality	M0: Unadjusted	2319	244	1.207	0.845	1.722	0.301
Cancer mortality	M1: Demographic-adjusted	2319	244	0.722	0.499	1.045	0.084
Cancer mortality	M2: Fully adjusted core model	2319	244	0.723	0.492	1.064	0.100

Figure 2C source data. Restricted-period mortality models, NHANES 1999-2014

Outcome	Model	n	Deaths	HR	95% CI lower	95% CI upper	P value
Cardiovascular composite mortality	M0: Unadjusted	1765	283	1.175	0.856	1.613	0.319
Cardiovascular composite mortality	M1: Demographic-adjusted	1765	283	0.557	0.398	0.781	<0.001

Outcome	Model	n	Deaths	HR	95% CI lower	95% CI upper	P value
Cardiovascular composite mortality	M2: Fully adjusted core model	1765	283	0.589	0.417	0.832	0.003
Chronic lower respiratory disease mortality	M0: Unadjusted	1765	152	1.600	1.090	2.349	0.016
Chronic lower respiratory disease mortality	M1: Demographic-adjusted	1765	152	0.786	0.531	1.163	0.229
Chronic lower respiratory disease mortality	M2: Fully adjusted core model	1765	152	1.182	0.758	1.845	0.461

Hazard ratios compare post-diagnostic quitters with persistent smokers.

Figure 3A source data. Landmark analyses

Outcome	Analysis	Landmark, months	n	Deaths	HR	95% CI lower	95% CI upper	P value
All-cause mortality	Baseline	0	2319	999	0.896	0.735	1.092	0.276
Heart-disease mortality	Baseline	0	2319	272	0.586	0.422	0.815	0.001
All-cause mortality	12-month landmark	12	2196	876	0.863	0.698	1.067	0.174
Heart-disease mortality	12-month landmark	12	2196	233	0.567	0.389	0.825	0.003
All-cause mortality	24-month landmark	24	1958	768	0.849	0.680	1.060	0.149
Heart-disease mortality	24-month landmark	24	1958	205	0.622	0.418	0.927	0.020
All-cause mortality	36-month landmark	36	1742	662	0.803	0.637	1.013	0.064
Heart-disease mortality	36-month landmark	36	1742	175	0.605	0.396	0.922	0.019

Figure 3B source data. Heart-disease mortality sensitivity analyses

Analysis	n	Deaths	HR	95% CI lower	95% CI upper	P value	Status
Primary fully adjusted model	2319	272	0.586	0.422	0.815	0.001	ok
No calendar-cycle adjustment	2319	272	0.595	0.424	0.834	0.003	ok
Restricted to NHANES 1999–2016	2048	264	0.577	0.414	0.805	0.001	ok
Strict quit-age definition	2093	235	0.592	0.411	0.853	0.005	ok
MEC-weighted extended model	1943	212	0.534	0.361	0.791	0.002	ok
Additional smoking-burden adjustment	2239	260	0.563	0.397	0.800	0.001	ok
Restricted to cardiovascular disease	1371	213	0.603	0.422	0.860	0.005	ok
Restricted to chronic lung disease	1356	136	0.620	0.400	0.962	0.033	ok
Survey × overlap weighting	2319	272	0.626	0.453	0.864	0.004	ok
Overlap weighting plus outcome adjustment	2319	272	0.579	0.416	0.807	0.001	ok
Exclude quit-age discrepancy >1 year	2199	249	0.520	0.370	0.733	<0.001	ok
Exclude quit-age discrepancy >2 years	2236	252	0.529	0.376	0.744	<0.001	ok

Analysis	n	Deaths	HR	95% CI lower	95% CI upper	P value	Status
Exclude quit-age discrepancy >5 years	2279	261	0.556	0.395	0.782	<0.001	ok
Directly reported quit age only	2117	258	0.577	0.410	0.813	0.002	ok
Omit years since diagnosis	2319	272	0.584	0.421	0.811	0.001	ok
Omit pre-diagnosis smoking duration	2319	272	0.586	0.421	0.816	0.002	ok

Hazard ratios compare post-diagnostic quitters with persistent smokers. Leave-one-covariate-out models are included in the final panel.

Figure 4A source data. Covariate balance before and after overlap weighting

Covariate	Survey weight only: absolute ASD	Survey x overlap weight: absolute ASD
Years since first diagnosis	0.719684	0.000001
Age, years	0.679367	0.000002
Sex: Male	0.195958	0.000005
Sex: Female	0.195958	0.000005
Race/ethnicity: Non-Hispanic White	0.183944	0.000012
Cardiopulmonary disease phenotype: Chronic lung disease only	0.165488	0.000005
Race/ethnicity: Non-Hispanic Black	0.143277	0.000019
Race/ethnicity: Other/Multiracial	0.135130	0.000001
Educational attainment: High school or less	0.130243	0.000005
Educational attainment: Some college or above	0.128327	0.000005
Cardiopulmonary disease phenotype: Both cardiovascular and lung disease	0.125296	0.000003
Pre-diagnosis smoking duration, years	0.092930	0.000000
Cardiopulmonary disease phenotype: Cardiovascular disease only	0.072198	0.000005
Educational attainment: Unknown	0.039784	0.000030
Survey calendar year	0.023429	0.000007
Cardiopulmonary disease phenotype: Other	0.019740	0.003462
Race/ethnicity: Hispanic	0.000882	0.000001

Poverty-income ratio was not included in the core propensity-score model and was not displayed in the final Figure 4A.

Figure 4B source data. Prespecified exploratory subgroup estimates for heart-disease mortality

Subgroup	Level	n	Deaths	HR	95% CI lower	95% CI upper	P value
Age group	40–64 years	1290	94	0.339	0.174	0.660	0.001
Age group	≥65 years	1029	178	0.760	0.520	1.110	0.156
Sex	Male	1290	171	0.482	0.329	0.705	<0.001
Sex	Female	1029	101	0.771	0.489	1.218	0.266
Disease phenotype	Cardiovascular disease only	963	136	0.502	0.332	0.761	0.001
Disease phenotype	Chronic lung disease only	947	59	0.365	0.183	0.728	0.004
Disease phenotype	Both cardiovascular and lung disease	408	77	1.010	0.549	1.860	0.973

Hazard ratios compare post-diagnostic quitters with persistent smokers and were adjusted for the core-model covariates other than the stratifying variable.

STROBE checklist for observational studies

Post-diagnostic smoking cessation and heart-disease mortality among adults smoking at cardiopulmonary disease diagnosis: An NHANES linked-mortality analysis

The study is a secondary observational analysis of pooled cross-sectional NHANES cycles linked to prospective mortality follow-up. The checklist below uses the STROBE reporting domains and points to the revised clean manuscript and supplementary material.

Item	Section	STROBE recommendation	Reported in revised manuscript
1	Title and abstract	Indicate the study design with a commonly used term in the title or abstract; provide an informative and balanced summary.	Title, page 1, lines 2-4; Abstract, pages 1-2, lines 12-39.
2	Background/rationale	Explain the scientific background and rationale.	Introduction, pages 2-3, lines 43-65.
3	Objectives	State specific objectives, including prespecified hypotheses.	Introduction, page 3, lines 66-73.
4	Study design	Present key elements of study design early.	Abstract Methods, page 1, lines 18-25; Methods, page 3, lines 75-81.
5	Setting	Describe setting, locations, and relevant dates.	Methods, pages 3-5, lines 75-116; mortality follow-up through 31 December 2019.
6	Participants	Give eligibility criteria and methods of selection.	Methods - Study population, pages 3-4, lines 82-93; Figure 1A; Supplementary Table S1.
7	Variables	Clearly define outcomes, exposures, predictors, confounders, and effect modifiers.	Exposure, pages 4, lines 94-107; outcomes, pages 4-5, lines 108-116; covariates, page 5, lines 117-128; subgroup variables, page 6, lines 166-170.
8	Data sources/measurement	For each variable, give sources and methods of assessment.	Methods, pages 3-6, lines 75-173; Supplementary methods and Tables S2-S4, S6-S9.
9	Bias	Describe efforts to address potential sources of bias.	Statistical analysis, pages 6, lines 150-170; sensitivity analyses, page 8, lines 204-231; Discussion limitations, pages 9-10, lines 247-284.
10	Study size	Explain how study size was arrived at.	All eligible NHANES participants were screened; Methods, pages 3-4, lines 82-93; Figure 1A; Supplementary Table S1.
11	Quantitative variables	Explain how quantitative variables were handled.	Covariates and scaling, page 5, lines 117-128; model specifications, page 6, lines 144-149.
12	Statistical methods	Describe confounding control, subgroup/interaction methods, missing data, sampling strategy, sensitivity analyses.	Statistical analysis, pages 5-6, lines 129-173; Tables 2-5; Supplementary Tables S4-S13.
13	Participants	Report numbers at each stage and reasons for non-participation.	Results, page 7, lines 181-188; Figure 1A; Supplementary Table S1.
14	Descriptive data	Give participant characteristics and missing data.	Results, page 7, lines 181-188; Table 1; Supplementary Tables S2-S4.
15	Outcome data	Report numbers of outcome events.	Results, pages 7-8, lines 191-202; Table 2; Supplementary Table S6.
16	Main results	Give unadjusted and adjusted estimates with precision; clarify confounders.	Results, pages 7-8, lines 191-202; Table 2 and Figure 2; model covariates described at page 6, lines 144-149.
17	Other analyses	Report subgroup and sensitivity analyses.	Results, pages 8-9, lines 204-238; Tables 3-5; Figures 3-4; Supplementary Tables S5, S7-S13.
18	Key results	Summarize key results with reference to objectives.	Discussion, page 9, lines 241-246.

Item	Section	STROBE recommendation	Reported in revised manuscript
19	Limitations	Discuss limitations, direction, and magnitude of potential bias.	Discussion, pages 9-10, lines 247-284.
20	Interpretation	Give cautious overall interpretation in light of objectives, limitations, multiplicity, and other evidence.	Discussion and Conclusions, pages 9-11, lines 241-294.
21	Generalisability	Discuss external validity.	Discussion, pages 9-10, lines 253-288; Conclusions, page 11, lines 289-294.
22	Funding	Give source and role of funders.	Declarations - Funding, page 11, line 301; revised Title Page.

Page and line numbers refer to 01_TID-02054-2026-01_Revised_Manuscript_Clean.docx. Supplementary-table references refer to the revised Supplementary Material.

Supplementary Code
==================

Manuscript title:
Post-diagnostic smoking cessation and heart-disease mortality among adults
smoking at cardiopulmonary disease diagnosis: an NHANES linked-mortality analysis

Manuscript ID: TID-02054-2026-01

Contents
----
1. Supplementary_Code_1_Complete_Analysis.R
   Complete integrated analysis workflow, including data harmonization, survey-weighted
   mortality models, sensitivity analyses, multicollinearity diagnostics, proportional
   hazards diagnostics, tables, figures, and source-data outputs.

2. Supplementary_Code_2_Final_Figure_Patch.R
   Final figure-only patch. It does not refit statistical models. It removes the zero-count
   exclusion annotation from Figure 1A and excludes poverty-income ratio from Figure 4A,
   while retaining the final Figure 2 and Figure 3 files unchanged.

Execution notes

- The complete analysis script was run under R 4.6.0 on Windows.
- Before execution, edit PROJECT_PARENT near the beginning of the complete analysis script
  to point to the local project directory.
- The script expects the locked NHANES cycle files and mortality files in the documented
  00_raw_data directory structure.
- The figure-only patch expects the final figure package created by the complete workflow.
- Numerical data underlying Figures 1-4 are supplied separately in the Figure Source Data file.
- No confidential participant-level data are included in this archive.

```r
########################################################################################
# 项目：心肺疾病诊断后戒烟与长期死亡风险——NHANES 1999–2018最终整合分析
# 文件：从锁定00_raw_data开始，一次运行全部主分析、返修分析、补充诊断、单面板图、拼图与源数据
# 版本：TID_MAJOR_REVISION_FINAL_ALL_IN_ONE_COMPLETE_RUN_v1_2026-07-17
#
# 使用说明：
# 1) 不要删除上次项目。脚本只读取上次项目中的00_raw_data，并在TID_MAJOR_REVISION_2026中生成全新输出。
# 2) 本脚本只读取锁定00_raw_data中的NHANES周期RDS和死亡DAT，不下载任何研究数据，
#    也不修改00_raw_data。
# 3) 脚本重新完成：合并、变量协调、复杂抽样权重、主模型、次要结局、
#    landmark、扩展协变量、吸烟负担、重叠加权、亚组、交互作用、
#    戒烟年龄不一致排除敏感性分析及比例风险假设检查。
# 4) 先完整复现上次结果，再执行TID大修新增分析和表图重构：
#    VIF/GVIF、调查加权生存曲线、1999–2014三层模型、流程图和Table 1–2重构。
# 5) 所有正式生存模型使用survey::svycoxph；主要暴露的比例风险假设使用
#    survival::cox.zph诊断。
########################################################################################

rm(list = ls())

# 本最终脚本整合：
# 1) 完整原始数据重建与变量协调；
# 2) 复杂抽样主模型、次要结局、landmark、重叠加权、亚组和戒烟年龄敏感性分析；
# 3) TID大修所需流程图、加权生存曲线、限定时期M0/M1/M2和表格重构；
# 4) VIF/GVIF、leave-one-covariate-out和修正版比例风险诊断；
# 5) Figure 1–4全部单面板600 dpi TIFF、完整拼图、对应CSV源数据；
# 6) 不生成任何ZIP压缩包。
#
# 注意：本脚本不下载研究数据，不修改锁定的00_raw_data。
########################################################################################

# ==============================================================================
# 0. 用户设置
# ==============================================================================

PROJECT_PARENT <- "C:/Users/33652/Desktop/Q"

# 锁定的上次分析项目：只读取其中的00_raw_data和旧结果，不修改该文件夹。
SOURCE_PROJECT_NAME <- "CARDIOPULMONARY_SMOKING_REANALYSIS_FINAL_1999_2018"
SOURCE_PROJECT_ROOT <- file.path(PROJECT_PARENT, SOURCE_PROJECT_NAME)

# 本次大修独立输出项目：全部重新分析结果写入此处。
PROJECT_NAME    <- "TID_MAJOR_REVISION_FINAL_ALL_IN_ONE"
PROJECT_ROOT    <- file.path(PROJECT_PARENT, PROJECT_NAME)

# 为避免上次中断运行留下的旧文件混入本次结果，默认清空本脚本自己的输出目录。
# 不会删除SOURCE_PROJECT_ROOT或任何00_raw_data。
CLEAN_OUTPUT_BEFORE_RUN <- TRUE
```

```
if (CLEAN_OUTPUT_BEFORE_RUN && dir.exists(PROJECT_ROOT)) {
  unlink(PROJECT_ROOT, recursive = TRUE, force = TRUE)
}

RAW_ROOT <- file.path(SOURCE_PROJECT_ROOT, "00_raw_data")

# 兼容两种实际目录结构：
# A. Q/CARDIOPULMONARY_SMOKING_REANALYSIS_FINAL_1999_2018/00_raw_data
# B. Q/00_raw_data
if (!dir.exists(RAW_ROOT) && dir.exists(file.path(PROJECT_PARENT, "00_raw_data"))) {
  RAW_ROOT <- file.path(PROJECT_PARENT, "00_raw_data")
}

RAW_NHANES <- file.path(RAW_ROOT, "NHANES")
RAW_MORT   <- file.path(RAW_ROOT, "NHANES_mortality")

MIN_AGE <- 40
LANDMARK_MONTHS <- c(12, 24, 36)
MIN_EVENTS_MODEL <- 10
MIN_EVENTS_SUBGROUP <- 15
WRITE_TIFF <- TRUE
WRITE_PDF <- TRUE
DPI <- 600
BASE_FAMILY <- "Times New Roman"
MORTALITY_VINTAGE <- "2019"
AUTO_INSTALL_PACKAGES <- TRUE

if (!dir.exists(RAW_NHANES) || !dir.exists(RAW_MORT)) {
  stop(
    "未找到00_raw_data中的NHANES或NHANES_mortality文件夹。应位于：\n",
    RAW_NHANES, "\n", RAW_MORT
  )
}

# ==============================================================================
# 1. 安装并载入R包
# ==============================================================================

required_packages <- c(
  "haven", "readr", "dplyr", "tidyr", "purrr", "stringr", "tibble",
  "survey", "survival", "ggplot2", "patchwork", "scales", "fs"
)
optional_packages <- c("EValue")

installed_now <- rownames(installed.packages())
if (AUTO_INSTALL_PACKAGES) {
  for (pkg in c(required_packages, optional_packages)) {
    if (!pkg %in% installed_now) {
      try(install.packages(pkg, repos = "https://cloud.r-project.org", dependencies
= TRUE), silent = TRUE)
```

```r
    }
  }
}
missing_required <- required_packages[!vapply(required_packages, requireNamespace,
logical(1), quietly = TRUE)]
if (length(missing_required) > 0) {
  stop("缺少必需R包:", paste(missing_required, collapse = ", "))
}

suppressPackageStartupMessages({
  library(haven); library(readr); library(dplyr); library(tidyr); library(purrr)
  library(stringr); library(tibble); library(survey); library(survival)
  library(ggplot2); library(patchwork); library(scales); library(fs)
})
HAS_EVALUE <- requireNamespace("EValue", quietly = TRUE)
options(stringsAsFactors = FALSE)
options(timeout = 1200)
options(survey.lonely.psu = "adjust")
options(warn = 1)

# ===============================================================================
# 2. 周期、模块和输出文件夹
# ===============================================================================

NHANES_CYCLES <- tibble::tribble(
  ~cycle,      ~suffix, ~begin_year,
  "1999-2000", "",      1999,
  "2001-2002", "_B",    2001,
  "2003-2004", "_C",    2003,
  "2005-2006", "_D",    2005,
  "2007-2008", "_E",    2007,
  "2009-2010", "_F",    2009,
  "2011-2012", "_G",    2011,
  "2013-2014", "_H",    2013,
  "2015-2016", "_I",    2015,
  "2017-2018", "_J",    2017
) %>% mutate(cycle_index = row_number(), survey_midyear = begin_year + 0.5)

MODULE_PLAN <- tibble::tribble(
  ~module, ~required, ~purpose,
  "DEMO", TRUE,  "人口学、权重及抽样设计",
  "MCQ",  TRUE,  "心肺疾病与诊断年龄",
  "SMQ",  TRUE,  "吸烟状态、戒烟时间与强度",
  "BMX",  FALSE, "BMI敏感性分析",
  "BPQ",  FALSE, "高血压扩展变量",
  "DIQ",  FALSE, "糖尿病扩展变量"
)

DIRS <- list(
  raw_nhanes = RAW_NHANES,
```

```
  raw_mort = RAW_MORT,
  code = file.path(PROJECT_ROOT, "01_code"),
  processed = file.path(PROJECT_ROOT, "02_processed_data"),
  audit = file.path(PROJECT_ROOT, "03_audit"),
  results = file.path(PROJECT_ROOT, "04_results"),
  figures = file.path(PROJECT_ROOT, "05_figures"),
  source_data = file.path(PROJECT_ROOT, "05_figures", "source_data"),
  tables = file.path(PROJECT_ROOT, "06_tables"),
  supp_tables = file.path(PROJECT_ROOT, "07_supplementary_tables"),
  reports = file.path(PROJECT_ROOT, "08_reports"),
  logs = file.path(PROJECT_ROOT, "09_logs")
)
invisible(lapply(DIRS[names(DIRS) != "raw_nhanes" & names(DIRS) != "raw_mort"],
fs::dir_create))

run_stamp <- format(Sys.time(), "%Y%m%d_%H%M%S")
log_file <- file.path(DIRS$logs, paste0("final_integrated_reanalysis_log_",
run_stamp, ".txt"))
sink(log_file, split = TRUE)
on.exit({
  while (sink.number() > 0) sink()
}, add = TRUE)

cat("============================================================\n")
cat("NHANES 1999-2018 final integrated reanalysis from 00_raw_data\n")
cat("Started: ", format(Sys.time()), "\n", sep = "")
cat("Project root: ", PROJECT_ROOT, "\n", sep = "")
cat("Data download: DISABLED\n")
cat("============================================================\n\n")

# ============================================================================
# 3. 通用函数
# ============================================================================

`%||%` <- function(x, y) if (is.null(x) || length(x) == 0) y else x

strip_labels <- function(x) {
  if (inherits(x, "haven_labelled")) x <- haven::zap_labels(x)
  if (is.factor(x)) x <- as.character(x)
  x
}

normalize_df <- function(df) {
  df <- as.data.frame(df)
  names(df) <- toupper(names(df))
  df[] <- lapply(df, strip_labels)
  df
}

normalize_text <- function(x) {
```

```
  x <- strip_labels(x)
  out <- toupper(trimws(as.character(x)))
  out[out %in% c("", "NA", "N/A", ".", "<NA>")] <- NA_character_
  out <- gsub("[[:punct:]]+", " ", out)
  out <- gsub("\\s+", " ", trimws(out))
  out
}

safe_numeric <- function(x) {
  x <- strip_labels(x)
  if (is.numeric(x) || is.integer(x)) return(as.numeric(x))
  suppressWarnings(as.numeric(trimws(as.character(x))))
}

clean_yes_no <- function(x) {
  ch <- normalize_text(x)
  num <- suppressWarnings(as.numeric(ch))
  out <- rep(NA_integer_, length(ch))
  out[num == 1 | ch %in% c("YES", "Y")] <- 1L
  out[num == 2 | ch %in% c("NO", "N")] <- 0L
  out[num %in% c(7, 9, 77, 99, 777, 999) |
        grepl("REFUSED|DON T KNOW|DONT KNOW|UNKNOWN", ch %||% "")] <- NA_integer_
  out
}

clean_smq040 <- function(x) {
  ch <- normalize_text(x)
  num <- suppressWarnings(as.numeric(ch))
  out <- rep(NA_integer_, length(ch))
  out[num == 1 | grepl("^EVERY DAY", ch %||% "")] <- 1L
  out[num == 2 | grepl("^SOME DAY", ch %||% "")] <- 2L
  out[num == 3 | grepl("^NOT AT ALL|^NONE$", ch %||% "")] <- 3L
  out[num %in% c(7, 9) | grepl("REFUSED|DON T KNOW|DONT KNOW", ch %||% "")] <-
NA_integer_
  out
}

clean_time_unit <- function(x) {
  ch <- normalize_text(x)
  num <- suppressWarnings(as.numeric(ch))
  out <- rep(NA_integer_, length(ch))
  out[num == 1 | grepl("^DAY", ch %||% "")] <- 1L
  out[num == 2 | grepl("^WEEK", ch %||% "")] <- 2L
  out[num == 3 | grepl("^MONTH", ch %||% "")] <- 3L
  out[num == 4 | grepl("^YEAR", ch %||% "")] <- 4L
  out[num %in% c(7, 9) | grepl("REFUSED|DON T KNOW|DONT KNOW", ch %||% "")] <-
NA_integer_
  out
}
```

```
clean_sex_label <- function(x) {
  ch <- normalize_text(x); num <- suppressWarnings(as.numeric(ch))
  dplyr::case_when(
    num == 1 | grepl("^MALE$", ch %||% "") ~ "Male",
    num == 2 | grepl("^FEMALE$", ch %||% "") ~ "Female",
    TRUE ~ "Unknown"
  )
}

clean_race_label <- function(x) {
  ch <- normalize_text(x); num <- suppressWarnings(as.numeric(ch))
  dplyr::case_when(
    num %in% c(1, 2) | grepl("MEXICAN AMERICAN|OTHER HISPANIC", ch %||% "") ~
"Hispanic",
    num == 3 | grepl("NON HISPANIC WHITE", ch %||% "") ~ "Non-Hispanic White",
    num == 4 | grepl("NON HISPANIC BLACK", ch %||% "") ~ "Non-Hispanic Black",
    num %in% c(5, 6, 7) | grepl("ASIAN|OTHER RACE|MULTI RACIAL|MULTIRACIAL", ch %||%
"") ~ "Other/Multiracial",
    TRUE ~ "Unknown"
  )
}

clean_education_label <- function(x) {
  ch <- normalize_text(x); num <- suppressWarnings(as.numeric(ch))
  dplyr::case_when(
    num %in% c(1, 2, 3) |
      grepl("LESS THAN 9|9 11TH|HIGH SCHOOL|GED", ch %||% "") ~ "High school or
less",
    num %in% c(4, 5) |
      grepl("SOME COLLEGE|AA DEGREE|COLLEGE GRADUATE|ABOVE", ch %||% "") ~ "Some
college or above",
    TRUE ~ "Unknown"
  )
}

clean_age <- function(x, max_age = 120, zero_is_missing = FALSE) {
  x <- safe_numeric(x)
  if (zero_is_missing) x[x == 0] <- NA_real_
  x[!is.finite(x) | x < 0 | x > max_age | x %in% c(777, 999, 7777, 9999)] <-
NA_real_
  x
}

clean_positive <- function(x, upper = Inf, zero_allowed = TRUE) {
  x <- safe_numeric(x)
  if (!zero_allowed) x[x == 0] <- NA_real_
  x[!is.finite(x) | x < 0 | x > upper | x %in% c(77, 99, 777, 999, 7777, 9999,
77777, 99999)] <- NA_real_
  x
}
```

```
get_first <- function(df, candidates, default = NA_real_) {
  hit <- toupper(candidates)
  hit <- hit[hit %in% names(df)]
  if (length(hit) == 0) return(rep(default, nrow(df)))
  df[[hit[1]]]
}

get_num_coalesce <- function(df, candidates, default = NA_real_) {
  hit <- toupper(candidates); hit <- hit[hit %in% names(df)]
  if (length(hit) == 0) return(rep(default, nrow(df)))
  vals <- lapply(hit, function(v) safe_numeric(df[[v]]))
  Reduce(function(x, y) dplyr::coalesce(x, y), vals)
}

get_yes_coalesce <- function(df, candidates) {
  hit <- toupper(candidates); hit <- hit[hit %in% names(df)]
  if (length(hit) == 0) return(rep(NA_integer_, nrow(df)))
  vals <- lapply(hit, function(v) clean_yes_no(df[[v]]))
  Reduce(function(x, y) dplyr::coalesce(x, y), vals)
}

get_age_coalesce <- function(df, candidates, max_age = 120, zero_is_missing = FALSE)
{
  clean_age(get_num_coalesce(df, candidates), max_age = max_age, zero_is_missing =
zero_is_missing)
}
get_num <- function(df, candidates) get_num_coalesce(df, candidates)
get_yes <- function(df, candidates) get_yes_coalesce(df, candidates)
get_age <- function(df, candidates) get_age_coalesce(df, candidates)

row_any_yes <- function(...) {
  mat <- do.call(cbind, list(...))
  any_yes <- rowSums(mat == 1, na.rm = TRUE) > 0
  all_missing <- rowSums(!is.na(mat)) == 0
  as.integer(ifelse(any_yes, 1L, ifelse(all_missing, NA_integer_, 0L)))
}

row_min_na <- function(...) {
  mat <- do.call(cbind, list(...))
  apply(mat, 1, function(z) {
    z <- z[is.finite(z)]
    if (length(z) == 0) NA_real_ else min(z)
  })
}

safe_quantile <- function(x, prob) {
  x <- safe_numeric(x); x <- x[is.finite(x)]
  if (length(x) == 0) return(NA_real_)
  as.numeric(stats::quantile(x, probs = prob, names = FALSE, na.rm = TRUE))
```

```
}

safe_max <- function(x) {
  x <- safe_numeric(x); x <- x[is.finite(x)]
  if (length(x) == 0) NA_real_ else max(x)
}

weighted_mean_safe <- function(x, w) {
  x <- safe_numeric(x); w <- safe_numeric(w)
  ok <- is.finite(x) & is.finite(w) & w > 0
  if (!any(ok)) return(NA_real_)
  sum(x[ok] * w[ok]) / sum(w[ok])
}
weighted_var_safe <- function(x, w) {
  x <- safe_numeric(x); w <- safe_numeric(w)
  ok <- is.finite(x) & is.finite(w) & w > 0
  if (sum(ok) < 2) return(NA_real_)
  mu <- weighted_mean_safe(x[ok], w[ok])
  sum(w[ok] * (x[ok] - mu)^2) / sum(w[ok])
}
weighted_sd_safe <- function(x, w) sqrt(weighted_var_safe(x, w))
weighted_prop_safe <- function(x, w, level) {
  w <- safe_numeric(w); ok <- !is.na(x) & is.finite(w) & w > 0
  if (!any(ok)) return(NA_real_)
  sum(w[ok] * as.numeric(x[ok] == level)) / sum(w[ok])
}
smd_continuous <- function(x, treat, w) {
  treat <- as.integer(treat)
  m1 <- weighted_mean_safe(x[treat == 1], w[treat == 1]); m0 <-
weighted_mean_safe(x[treat == 0], w[treat == 0])
  v1 <- weighted_var_safe(x[treat == 1], w[treat == 1]); v0 <-
weighted_var_safe(x[treat == 0], w[treat == 0])
  den <- sqrt((v1 + v0) / 2)
  if (!is.finite(den) || den == 0) return(NA_real_)
  (m1 - m0) / den
}
smd_binary_level <- function(x, treat, w, level) {
  treat <- as.integer(treat)
  p1 <- weighted_prop_safe(x[treat == 1], w[treat == 1], level); p0 <-
weighted_prop_safe(x[treat == 0], w[treat == 0], level)
  pbar <- (p1 + p0) / 2; den <- sqrt(pbar * (1 - pbar))
  if (!is.finite(den) || den == 0) return(NA_real_)
  (p1 - p0) / den
}
format_hr <- function(hr, lo, hi) ifelse(is.na(hr), NA_character_, sprintf("%.2f
(%.2f–%.2f)", hr, lo, hi))
format_p <- function(p) ifelse(is.na(p), NA_character_, ifelse(p < 0.001, "<0.001",
sprintf("%.3f", p)))

save_tiff <- function(plot, filename, width, height) {
```

```
  if (!WRITE_TIFF) return(invisible(NULL))
  ggplot2::ggsave(filename, plot, width = width, height = height, units = "in", dpi
= DPI,
                  device = "tiff", compression = "lzw", limitsize = FALSE)
}

save_pub_figure <- function(plot, stem, width, height) {
  if (WRITE_TIFF) {
    ggplot2::ggsave(
      filename = file.path(DIRS$figures, paste0(stem, ".tiff")),
      plot = plot,
      width = width,
      height = height,
      units = "in",
      dpi = DPI,
      device = "tiff",
      compression = "lzw",
      limitsize = FALSE
    )
  }
  if (WRITE_PDF) {
    ggplot2::ggsave(
      filename = file.path(DIRS$figures, paste0(stem, ".pdf")),
      plot = plot,
      width = width,
      height = height,
      units = "in",
      device = grDevices::cairo_pdf,
      limitsize = FALSE
    )
  }
}
pub_theme <- function(base_size = 10) {
  ggplot2::theme_bw(base_size = base_size, base_family = BASE_FAMILY) +
    ggplot2::theme(plot.title = element_text(face = "bold", hjust = 0),
                   plot.subtitle = element_text(hjust = 0), legend.position =
"bottom",
                   legend.title = element_blank(), panel.grid.minor =
element_blank(),
                   strip.background = element_rect(fill = "grey95"), strip.text =
element_text(face = "bold"))
}
safe_write_csv <- function(x, path) readr::write_csv(as.data.frame(x), path, na =
"")

complete_design <- function(design, vars) {
  vars <- unique(vars); vars <- vars[vars %in% names(design$variables)]
  ok <- stats::complete.cases(design$variables[, vars, drop = FALSE])
  design$variables$.complete_model_internal <- ok
  out <- base::subset(design, .complete_model_internal)
```

```
  out$variables <- droplevels(out$variables)
  out
}

bind_rows_harmonized <- function(lst) {
  lst <- lapply(lst, normalize_df)
  all_names <- Reduce(union, lapply(lst, names))
  class_map <- lapply(all_names, function(v) unique(na.omit(vapply(lst, function(d)
if (v %in% names(d)) class(d[[v]])[1] else NA_character_, character(1)))))
  names(class_map) <- all_names
  mixed <- names(class_map)[vapply(class_map, length, integer(1)) > 1]
  lst2 <- lapply(lst, function(d) {
    for (v in setdiff(all_names, names(d))) d[[v]] <- NA
    d <- d[, all_names, drop = FALSE]
    if (length(mixed) > 0) d[mixed] <- lapply(d[mixed], as.character)
    d
  })
  dplyr::bind_rows(lst2)
}

extract_svycox_term <- function(fit, term = "exposure_quit") {
  b <- stats::coef(fit); V <- stats::vcov(fit)
  if (!term %in% names(b)) return(tibble(term = term, logHR = NA_real_, SE =
NA_real_, HR = NA_real_, CI_low = NA_real_, CI_high = NA_real_, p = NA_real_))
  se <- sqrt(V[term, term]); z <- b[term] / se
  tibble(term = term, logHR = unname(b[term]), SE = unname(se), HR =
exp(unname(b[term])),
         CI_low = exp(unname(b[term]) - qnorm(0.975) * se), CI_high =
exp(unname(b[term]) + qnorm(0.975) * se),
         p = 2 * pnorm(abs(z), lower.tail = FALSE))
}

run_svycox <- function(design, time_var, event_var, rhs, analysis_label,
outcome_label,
                       term = "exposure_quit", min_events = MIN_EVENTS_MODEL) {
  needed <- unique(c(time_var, event_var, all.vars(as.formula(paste0("~", rhs)))))
  des <- complete_design(design, needed)
  n_model <- nrow(des$variables); n_events <- sum(des$variables[[event_var]] == 1,
na.rm = TRUE)
  n_quit <- sum(des$variables$exposure_quit == 1, na.rm = TRUE); n_persist <-
sum(des$variables$exposure_quit == 0, na.rm = TRUE)
  base <- tibble(analysis = analysis_label, outcome = outcome_label, time_variable =
time_var,
                 event_variable = event_var, formula_rhs = rhs, model_type =
"survey::svycoxph",
                 n = n_model, events = n_events, n_quit = n_quit, n_persistent =
n_persist,
                 design_df = tryCatch(survey::degf(des), error = function(e)
NA_real_))
  if (n_model == 0 || n_events < min_events || n_quit == 0 || n_persist == 0) {
```

```
    return(bind_cols(base, tibble(term = term, logHR = NA_real_, SE = NA_real_, HR =
NA_real_, CI_low = NA_real_, CI_high = NA_real_, p = NA_real_, status = "not_run",
error = "样本、事件数或暴露组不足")))
  }
  fml <- as.formula(paste0("survival::Surv(", time_var, ", ", event_var, ") ~ ",
rhs))
  tryCatch({
    fit <- suppressWarnings(survey::svycoxph(fml, design = des))
    est <- extract_svycox_term(fit, term)
    bind_cols(base, est) %>% mutate(status = if_else(is.finite(HR), "ok", "failed"),
error = NA_character_)
  }, error = function(e) bind_cols(base, tibble(term = term, logHR = NA_real_, SE =
NA_real_, HR = NA_real_, CI_low = NA_real_, CI_high = NA_real_, p = NA_real_, status
= "failed", error = conditionMessage(e))))
}

wald_interaction_p <- function(fit, subgroup_var) {
  b <- coef(fit); V <- vcov(fit); nms <- names(b)
  idx <- grep(paste0("(^exposure_quit:.*", subgroup_var, ")|(.*", subgroup_var,
":exposure_quit$)"), nms)
  if (length(idx) == 0) return(NA_real_)
  stat <- tryCatch(as.numeric(t(b[idx]) %*% solve(V[idx, idx, drop = FALSE],
b[idx])), error = function(e) NA_real_)
  if (!is.finite(stat)) return(NA_real_)
  pchisq(stat, df = length(idx), lower.tail = FALSE)
}
manual_evalue <- function(hr, lo, hi) {
  if (!is.finite(hr) || hr <= 0) return(tibble(E_value_point = NA_real_, E_value_CI
= NA_real_))
  rr <- if (hr < 1) 1 / hr else hr; point <- rr + sqrt(rr * (rr - 1)); ci_val <-
NA_real_
  if (hr < 1 && is.finite(hi) && hi < 1) { rr_ci <- 1 / hi; ci_val <- rr_ci +
sqrt(rr_ci * (rr_ci - 1)) }
  if (hr > 1 && is.finite(lo) && lo > 1) { rr_ci <- lo; ci_val <- rr_ci + sqrt(rr_ci
* (rr_ci - 1)) }
  tibble(E_value_point = point, E_value_CI = ci_val)
}

# ================================================================================
# 4. 只读取00_raw_data并重新合并
# ================================================================================

module_code <- function(module, suffix) paste0(module, suffix)
local_nhanes_path <- function(cycle, code) file.path(RAW_NHANES, paste0(cycle, "__",
code, ".rds"))

read_local_nhanes <- function(cycle, code, required = TRUE) {
  path <- local_nhanes_path(cycle, code)
  if (!file.exists(path)) {
    # 兼容文件名大小写差异
```

```
    hits <- list.files(RAW_NHANES, full.names = TRUE)
    target <- toupper(basename(path))
    hits <- hits[toupper(basename(hits)) == target]
    if (length(hits) > 0) path <- hits[1]
  }
  if (!file.exists(path)) {
    if (required) stop("缺少本地核心缓存:", cycle, " / ", code, "。本脚本不会下载。")
    return(list(ok = FALSE, data = NULL, path = NA_character_))
  }
  list(ok = TRUE, data = normalize_df(readRDS(path)), path = path)
}

read_mortality_fwf <- function(path) {
  lines <- readLines(path, warn = FALSE)
  out <- tibble(
    SEQN = suppressWarnings(as.numeric(trimws(substr(lines, 1, 6)))),
    ELIGSTAT = suppressWarnings(as.integer(trimws(substr(lines, 15, 15)))),
    MORTSTAT = suppressWarnings(as.integer(trimws(substr(lines, 16, 16)))),
    UCOD_LEADING = trimws(substr(lines, 17, 19)),
    DIABETES_MCOD = suppressWarnings(as.integer(trimws(substr(lines, 20, 20)))),
    HYPERTEN_MCOD = suppressWarnings(as.integer(trimws(substr(lines, 21, 21)))),
    PERMTH_INT = suppressWarnings(as.numeric(trimws(substr(lines, 43, 45)))),
    PERMTH_EXM = suppressWarnings(as.numeric(trimws(substr(lines, 46, 48))))
  ) %>% mutate(UCOD_LEADING = na_if(UCOD_LEADING, ""))
  if (nrow(out) == 0 || sum(out$ELIGSTAT == 1, na.rm = TRUE) < 1000) stop("死亡文件
解析异常:", path)
  out
}

mortality_path <- function(cycle) file.path(RAW_MORT, paste0("NHANES_", gsub("-",
"_", cycle), "_MORT_", MORTALITY_VINTAGE, "_PUBLIC.dat"))

cat("[1/14] 从00_raw_data读取并合并10个周期……\n")
cache_log <- list(); cycle_list <- list()
for (i in seq_len(nrow(NHANES_CYCLES))) {
  cyc <- NHANES_CYCLES$cycle[i]; suf <- NHANES_CYCLES$suffix[i]; byear <-
NHANES_CYCLES$begin_year[i]
  cat("  周期:", cyc, "\n", sep = "")
  module_data <- list()
  for (j in seq_len(nrow(MODULE_PLAN))) {
    mod <- MODULE_PLAN$module[j]; code <- module_code(mod, suf)
    res <- read_local_nhanes(cyc, code, MODULE_PLAN$required[j])
    cache_log[[length(cache_log) + 1]] <- tibble(cycle = cyc, begin_year = byear,
module = mod,
      table_code = code, required = MODULE_PLAN$required[j], success = res$ok,
      source_path = res$path, n = if (res$ok) nrow(res$data) else NA_integer_, p =
if (res$ok) ncol(res$data) else NA_integer_)
    if (res$ok) module_data[[mod]] <- res$data
  }
  mpath <- mortality_path(cyc)
```

```
  if (!file.exists(mpath)) stop("缺少本地死亡文件：", mpath)
  mort <- read_mortality_fwf(mpath)
  cache_log[[length(cache_log) + 1]] <- tibble(cycle = cyc, begin_year = byear,
module = "MORTALITY",
      table_code = basename(mpath), required = TRUE, success = TRUE, source_path =
mpath, n = nrow(mort), p = ncol(mort))

  merged <- module_data$DEMO %>% arrange(SEQN) %>% distinct(SEQN, .keep_all = TRUE)
  for (mod in setdiff(names(module_data), "DEMO")) {
    tmp <- module_data[[mod]] %>% arrange(SEQN) %>% distinct(SEQN, .keep_all = TRUE)
    dup <- intersect(setdiff(names(tmp), "SEQN"), names(merged))
    if (length(dup) > 0) tmp <- tmp %>% select(-all_of(dup))
    merged <- left_join(merged, tmp, by = "SEQN")
  }
  old_mort_cols <- intersect(c("ELIGSTAT", "MORTSTAT", "UCOD_LEADING",
"DIABETES_MCOD", "HYPERTEN_MCOD", "PERMTH_INT", "PERMTH_EXM"), names(merged))
  if (length(old_mort_cols) > 0) merged <- merged %>% select(-all_of(old_mort_cols))
  merged <- merged %>% left_join(mort, by = "SEQN") %>% mutate(CYCLE = cyc,
BEGIN_YEAR = byear)
  cycle_list[[cyc]] <- merged
  cat("    ", nrow(merged), "人，", ncol(merged), "列\n", sep = "")
}
all_raw <- bind_rows_harmonized(cycle_list)
safe_write_csv(bind_rows(cache_log), file.path(DIRS$audit,
"01_local_raw_cache_read_log.csv"))
saveRDS(all_raw, file.path(DIRS$processed, "01_merged_raw_1999_2018_rebuilt.rds"),
compress = "xz")

required_cols <- c("SEQN", "CYCLE", "BEGIN_YEAR", "RIDAGEYR", "RIAGENDR",
"RIDRETH1", "WTINT2YR", "WTMEC2YR", "SDMVPSU", "SDMVSTRA", "MCQ160B", "MCQ160C",
"MCQ160D", "MCQ160E", "MCQ160G", "MCQ160K", "SMQ020", "SMD030", "SMQ040",
"ELIGSTAT", "MORTSTAT", "UCOD_LEADING", "PERMTH_INT", "PERMTH_EXM")
miss <- setdiff(required_cols, names(all_raw))
if (length(miss) > 0) stop("合并数据缺少变量：", paste(miss, collapse = ", "))
if (anyDuplicated(paste(all_raw$CYCLE, get_num_coalesce(all_raw, "SEQN"), sep =
"__")) > 0) stop("周期内存在重复SEQN。")

cycle_preflight <- tibble(cycle = as.character(all_raw$CYCLE), begin_year =
safe_numeric(all_raw$BEGIN_YEAR)) %>% count(cycle, begin_year, name = "n") %>%
arrange(begin_year)
safe_write_csv(cycle_preflight, file.path(DIRS$audit,
"00_preflight_cycle_counts.csv"))
if (!all(NHANES_CYCLES$cycle %in% cycle_preflight$cycle)) stop("并非全部10个周期均已
合并。")

# ==============================================================================
# 5. 重新构造变量
# ==============================================================================

cat("[2/14] 重新构造疾病、吸烟、权重和死亡变量……\n")
```

```
age <- clean_age(get_first(all_raw, "RIDAGEYR"), 120)
sex_label <- clean_sex_label(get_first(all_raw, "RIAGENDR"))
race_label <- clean_race_label(get_first(all_raw, "RIDRETH1"))
education_label <- clean_education_label(get_first(all_raw, "DMDEDUC2"))
pir <- clean_positive(get_first(all_raw, "INDFMPIR"), 5)

wtint2yr <- clean_positive(get_first(all_raw, "WTINT2YR")); wtint4yr <-
clean_positive(get_first(all_raw, "WTINT4YR"))
wtmec2yr <- clean_positive(get_first(all_raw, "WTMEC2YR")); wtmec4yr <-
clean_positive(get_first(all_raw, "WTMEC4YR"))
sdmvpsu <- clean_positive(get_first(all_raw, "SDMVPSU")); sdmvstra <-
clean_positive(get_first(all_raw, "SDMVSTRA"))

hf_flag <- get_yes_coalesce(all_raw, "MCQ160B"); hf_age <- ifelse(hf_flag == 1,
get_age_coalesce(all_raw, c("MCQ180B", "MCD180B")), NA_real_)
chd_flag <- get_yes_coalesce(all_raw, "MCQ160C"); chd_age <- ifelse(chd_flag == 1,
get_age_coalesce(all_raw, c("MCQ180C", "MCD180C")), NA_real_)
angina_flag <- get_yes_coalesce(all_raw, "MCQ160D"); angina_age <-
ifelse(angina_flag == 1, get_age_coalesce(all_raw, c("MCQ180D", "MCD180D")),
NA_real_)
mi_flag <- get_yes_coalesce(all_raw, "MCQ160E"); mi_age <- ifelse(mi_flag == 1,
get_age_coalesce(all_raw, c("MCQ180E", "MCD180E")), NA_real_)
emphy_flag <- get_yes_coalesce(all_raw, "MCQ160G"); emphy_age <- ifelse(emphy_flag
== 1, get_age_coalesce(all_raw, c("MCQ180G", "MCD180G")), NA_real_)
bronch_flag <- get_yes_coalesce(all_raw, "MCQ160K"); bronch_age <-
ifelse(bronch_flag == 1, get_age_coalesce(all_raw, c("MCQ180K", "MCD180K")),
NA_real_)
cvd_any <- row_any_yes(hf_flag, chd_flag, angina_flag, mi_flag); lung_any <-
row_any_yes(emphy_flag, bronch_flag); cardiopulmonary_any <- row_any_yes(cvd_any,
lung_any)
cp_dx_age_raw <- row_min_na(hf_age, chd_age, angina_age, mi_age, emphy_age,
bronch_age)
cp_dx_age <- ifelse(is.finite(cp_dx_age_raw) & is.finite(age) & cp_dx_age_raw <=
age, cp_dx_age_raw, NA_real_)

smq020 <- clean_yes_no(get_first(all_raw, "SMQ020"))
smq040 <- clean_smq040(get_first(all_raw, "SMQ040"))
smoking_start_age <- get_age_coalesce(all_raw, "SMD030", zero_is_missing = TRUE)
smoking_start_age[!is.finite(age) | smoking_start_age > age] <- NA_real_

quit_age_direct <- get_age_coalesce(all_raw, "SMD055", zero_is_missing = TRUE)
quit_age_direct[!is.finite(age) | quit_age_direct > age] <- NA_real_
quit_time_q_raw <- clean_positive(get_first(all_raw, "SMQ050Q"), upper = 66666)
quit_time_unit <- clean_time_unit(get_first(all_raw, "SMQ050U"))
quit_duration_years <- dplyr::case_when(
  quit_time_q_raw == 66666 ~ NA_real_,
  quit_time_q_raw >= 0 & quit_time_q_raw < 10000 & quit_time_unit == 1 ~
quit_time_q_raw / 365.25,
  quit_time_q_raw >= 0 & quit_time_q_raw < 10000 & quit_time_unit == 2 ~
```

```
quit_time_q_raw / 52.1775,
  quit_time_q_raw >= 0 & quit_time_q_raw < 10000 & quit_time_unit == 3 ~
quit_time_q_raw / 12,
  quit_time_q_raw >= 0 & quit_time_q_raw < 10000 & quit_time_unit == 4 ~
quit_time_q_raw,
  TRUE ~ NA_real_
)
quit_age_derived <- ifelse(is.finite(age) & is.finite(quit_duration_years), age -
quit_duration_years, NA_real_)
quit_age_derived[!is.finite(quit_age_derived) | quit_age_derived < 0 |
quit_age_derived > age] <- NA_real_
quit_age <- dplyr::coalesce(quit_age_direct, quit_age_derived)
quit_age_source <- dplyr::case_when(
  is.finite(quit_age_direct) ~ "Direct SMD055",
  !is.finite(quit_age_direct) & is.finite(quit_age_derived) ~ "Derived SMQ050Q/U",
  quit_time_q_raw == 66666 ~ "Right-censored >=50 years; unclassified",
  TRUE ~ "Missing/unusable"
)
quit_age_discrepancy_years <- ifelse(is.finite(quit_age_direct) &
is.finite(quit_age_derived), abs(quit_age_direct - quit_age_derived), NA_real_)

cig_day_at_quit <- clean_positive(get_first(all_raw, "SMD057"), upper = 95,
zero_allowed = FALSE)
cig_day_current <- clean_positive(get_num_coalesce(all_raw, c("SMD070", "SMD090",
"SMD650")), upper = 95, zero_allowed = FALSE)

current_smoker_baseline <- dplyr::case_when(smq020 == 1 & smq040 %in% c(1, 2) ~ 1L,
smq020 == 1 & smq040 == 3 ~ 0L, TRUE ~ NA_integer_)
former_smoker_baseline <- dplyr::case_when(smq020 == 1 & smq040 == 3 ~ 1L, smq020 ==
1 & smq040 %in% c(1, 2) ~ 0L, TRUE ~ NA_integer_)
smoking_started_by_dx <- ifelse(smq020 == 1 & is.finite(smoking_start_age) &
is.finite(cp_dx_age), as.integer(smoking_start_age <= cp_dx_age), NA_integer_)
smoking_at_diagnosis <- dplyr::case_when(
  smoking_started_by_dx == 0 ~ 0L,
  smoking_started_by_dx == 1 & current_smoker_baseline == 1 ~ 1L,
  smoking_started_by_dx == 1 & former_smoker_baseline == 1 & is.finite(quit_age) &
quit_age >= cp_dx_age ~ 1L,
  smoking_started_by_dx == 1 & former_smoker_baseline == 1 & is.finite(quit_age) &
quit_age < cp_dx_age ~ 0L,
  TRUE ~ NA_integer_
)
postdiagnostic_quitter <- as.integer(smoking_at_diagnosis == 1 &
former_smoker_baseline == 1 & is.finite(quit_age) & quit_age >= cp_dx_age)
persistent_smoker <- as.integer(smoking_at_diagnosis == 1 & current_smoker_baseline
== 1)
exposure_classifiable <- as.integer(postdiagnostic_quitter == 1 | persistent_smoker
== 1)
exposure_quit <- dplyr::case_when(postdiagnostic_quitter == 1 ~ 1L,
persistent_smoker == 1 ~ 0L, TRUE ~ NA_integer_)
```

```
years_since_dx <- age - cp_dx_age; years_from_dx_to_quit <- quit_age - cp_dx_age;
years_since_quit <- age - quit_age
pre_dx_smoking_years <- cp_dx_age - smoking_start_age
pre_dx_smoking_years[!is.finite(pre_dx_smoking_years) | pre_dx_smoking_years < 0 |
pre_dx_smoking_years > 90] <- NA_real_
smoking_end_age <- ifelse(exposure_quit == 1, quit_age, age)
smoking_duration_proxy <- smoking_end_age - smoking_start_age
smoking_duration_proxy[!is.finite(smoking_duration_proxy) | smoking_duration_proxy <
0 | smoking_duration_proxy > 90] <- NA_real_
cig_per_day_proxy <- ifelse(exposure_quit == 1, cig_day_at_quit, cig_day_current)
pack_years_proxy <- (cig_per_day_proxy / 20) * smoking_duration_proxy
pack_years_proxy[!is.finite(pack_years_proxy) | pack_years_proxy < 0 |
pack_years_proxy > 300] <- NA_real_

bmi <- clean_positive(get_first(all_raw, "BMXBMI"), 100)
diabetes_q <- clean_yes_no(get_first(all_raw, "DIQ010")); hypertension_q <-
clean_yes_no(get_first(all_raw, "BPQ020"))
eligstat <- safe_numeric(get_first(all_raw, "ELIGSTAT")); mortstat <-
safe_numeric(get_first(all_raw, "MORTSTAT"))
ucod_leading <- trimws(as.character(get_first(all_raw, "UCOD_LEADING",
NA_character_)))
ucod_leading[ucod_leading %in% c("", "NA", ".")] <- NA_character_
ucod_num <- suppressWarnings(as.integer(ucod_leading));
ucod_leading[is.finite(ucod_num)] <- sprintf("%03d", ucod_num[is.finite(ucod_num)])
permth_int <- clean_positive(get_first(all_raw, "PERMTH_INT"), 500); permth_exm <-
clean_positive(get_first(all_raw, "PERMTH_EXM"), 500)
event_allcause <- ifelse(mortstat == 1, 1L, ifelse(mortstat == 0, 0L, NA_integer_))
event_heart <- ifelse(event_allcause == 1 & ucod_leading == "001", 1L,
ifelse(event_allcause %in% c(0, 1), 0L, NA_integer_))
event_cancer <- ifelse(event_allcause == 1 & ucod_leading == "002", 1L,
ifelse(event_allcause %in% c(0, 1), 0L, NA_integer_))
event_clrd_raw <- ifelse(event_allcause == 1 & ucod_leading == "003", 1L,
ifelse(event_allcause %in% c(0, 1), 0L, NA_integer_))
event_cerebro_raw <- ifelse(event_allcause == 1 & ucod_leading == "005", 1L,
ifelse(event_allcause %in% c(0, 1), 0L, NA_integer_))
event_cvd_composite_raw <- ifelse(event_allcause == 1 & ucod_leading %in% c("001",
"005"), 1L, ifelse(event_allcause %in% c(0, 1), 0L, NA_integer_))

begin_year <- safe_numeric(all_raw$BEGIN_YEAR); cycle <- as.character(all_raw$CYCLE)
wt_int_20y <- case_when(begin_year %in% c(1999, 2001) ~ wtint4yr / 5, begin_year
%in% c(2003, 2005, 2007, 2009, 2011, 2013, 2015, 2017) ~ wtint2yr / 10, TRUE ~
NA_real_)
wt_mec_20y <- case_when(begin_year %in% c(1999, 2001) ~ wtmec4yr / 5, begin_year
%in% c(2003, 2005, 2007, 2009, 2011, 2013, 2015, 2017) ~ wtmec2yr / 10, TRUE ~
NA_real_)
wt_int_18y_sens <- case_when(begin_year %in% c(1999, 2001) ~ wtint4yr * 2/9,
begin_year %in% c(2003, 2005, 2007, 2009, 2011, 2013, 2015) ~ wtint2yr / 9, TRUE ~
NA_real_)
wt_int_16y <- case_when(begin_year %in% c(1999, 2001) ~ wtint4yr / 4, begin_year
%in% c(2003, 2005, 2007, 2009, 2011, 2013) ~ wtint2yr / 8, TRUE ~ NA_real_)
```

```
cycle_index <- match(begin_year, NHANES_CYCLES$begin_year); survey_midyear <-
begin_year + 0.5; survey_cycle_2y_centered <- (survey_midyear - 2008.5)/2
survey_strata_uid <- interaction(begin_year, sdmvstra, drop = TRUE, lex.order =
TRUE)
survey_psu_uid <- interaction(begin_year, sdmvstra, sdmvpsu, drop = TRUE, lex.order
= TRUE)

analysis_all <- tibble(
  SEQN = get_num_coalesce(all_raw, "SEQN"), cycle, begin_year, cycle_index,
survey_midyear, survey_cycle_2y_centered,
  age, sex_label, race_label, education_label, pir, sdmvpsu, sdmvstra,
survey_strata_uid, survey_psu_uid,
  wtint2yr, wtint4yr, wtmec2yr, wtmec4yr, wt_int_20y, wt_mec_20y, wt_int_18y_sens,
wt_int_16y,
  hf_flag, chd_flag, angina_flag, mi_flag, emphy_flag, bronch_flag, cvd_any,
lung_any, cardiopulmonary_any,
  cp_dx_age, years_since_dx, smoking_start_age, smq020, smq040, quit_time_q_raw,
quit_time_unit, quit_duration_years,
  quit_age_direct, quit_age_derived, quit_age, quit_age_source,
quit_age_discrepancy_years,
  current_smoker_baseline, former_smoker_baseline, smoking_started_by_dx,
smoking_at_diagnosis,
  postdiagnostic_quitter, persistent_smoker, exposure_classifiable, exposure_quit,
  years_from_dx_to_quit, years_since_quit, pre_dx_smoking_years, cig_day_at_quit,
cig_day_current,
  cig_per_day_proxy, smoking_duration_proxy, pack_years_proxy, bmi, diabetes_q,
hypertension_q,
  eligstat, mortstat, ucod_leading, permth_int, permth_exm, time_int_years =
permth_int/12, time_exm_years = permth_exm/12,
  event_allcause, event_heart, event_cancer, event_cerebro_raw, event_clrd_raw,
event_cvd_composite_raw
) %>% mutate(
  sex_f = factor(sex_label, levels = c("Male", "Female", "Unknown")),
  race_f = factor(race_label, levels = c("Non-Hispanic White", "Hispanic",
"Non-Hispanic Black", "Other/Multiracial", "Unknown")),
  education_f = factor(education_label, levels = c("High school or less", "Some
college or above", "Unknown")),
  disease_f = factor(case_when(cvd_any == 1 & lung_any == 0 ~ "Cardiovascular
disease only", cvd_any == 0 & lung_any == 1 ~ "Chronic lung disease only", cvd_any
== 1 & lung_any == 1 ~ "Both cardiovascular and lung disease", TRUE ~ "Other"),
                     levels = c("Cardiovascular disease only", "Chronic lung disease
only", "Both cardiovascular and lung disease", "Other")),
  diabetes_f = factor(case_when(diabetes_q == 1 ~ "Yes", diabetes_q == 0 ~ "No",
TRUE ~ "Unknown"), levels = c("No", "Yes", "Unknown")),
  hypertension_f = factor(case_when(hypertension_q == 1 ~ "Yes", hypertension_q == 0
~ "No", TRUE ~ "Unknown"), levels = c("No", "Yes", "Unknown")),
  exposure_f = factor(ifelse(exposure_quit == 1, "Post-diagnostic quitter",
"Persistent smoker"), levels = c("Persistent smoker", "Post-diagnostic quitter")),
  age_group_f = factor(ifelse(age >= 65, "≥65 years", "40–64 years"), levels =
c("40–64 years", "≥65 years")),
```

```r
  age10 = age/10, years_since_dx5 = years_since_dx/5, pre_dx_smoking_years10 = pre_dx_smoking_years/10,
  bmi5 = bmi/5, pack_years10 = pack_years_proxy/10,
  strict_quit_definition = as.integer(exposure_quit == 0 | (exposure_quit == 1 & is.finite(years_from_dx_to_quit) & years_from_dx_to_quit > 0)),
  domain_primary = as.integer(coalesce(age >= MIN_AGE, FALSE) & coalesce(cardiopulmonary_any == 1, FALSE) & is.finite(cp_dx_age) & coalesce(smoking_at_diagnosis == 1, FALSE) & coalesce(exposure_classifiable == 1, FALSE) & coalesce(eligstat == 1, FALSE) & is.finite(permth_int) & permth_int > 0 & is.finite(wt_int_20y) & wt_int_20y > 0 & !is.na(survey_psu_uid) & !is.na(survey_strata_uid)),
  domain_mec_extended = as.integer(domain_primary == 1 & is.finite(permth_exm) & permth_exm > 0 & is.finite(wt_mec_20y) & wt_mec_20y > 0 & is.finite(bmi)),
  domain_18y_sens = as.integer(domain_primary == 1 & begin_year <= 2015 & is.finite(wt_int_18y_sens) & wt_int_18y_sens > 0),
  domain_16y = as.integer(domain_primary == 1 & begin_year <= 2013 & is.finite(wt_int_16y) & wt_int_16y > 0),
  event_cvd_composite_16y = ifelse(begin_year <= 2013, event_cvd_composite_raw, NA_integer_),
  event_clrd_16y = ifelse(begin_year <= 2013, event_clrd_raw, NA_integer_),
  qc_dx_after_baseline = as.integer(is.finite(cp_dx_age) & cp_dx_age > age),
  qc_quit_before_start = as.integer(is.finite(quit_age) & is.finite(smoking_start_age) & quit_age < smoking_start_age),
  qc_both_exposure_groups = as.integer(postdiagnostic_quitter == 1 & persistent_smoker == 1)
)

# 原始标签与重建频数审计:用于确认不再出现SMQ040全缺失。
raw_label_audit <- bind_rows(
  tibble(variable = "SMQ020_raw", value = as.character(get_first(all_raw, "SMQ020"))),
  tibble(variable = "SMQ040_raw", value = as.character(get_first(all_raw, "SMQ040"))),
  tibble(variable = "SMQ050U_raw", value = as.character(get_first(all_raw, "SMQ050U"))),
  tibble(variable = "RIAGENDR_raw", value = as.character(get_first(all_raw, "RIAGENDR"))),
  tibble(variable = "RIDRETH1_raw", value = as.character(get_first(all_raw, "RIDRETH1"))),
  tibble(variable = "DMDEDUC2_raw", value = as.character(get_first(all_raw, "DMDEDUC2")))
) %>% count(variable, value, name = "n", sort = TRUE)
safe_write_csv(raw_label_audit, file.path(DIRS$audit, "02a_raw_label_frequency_audit.csv"))

recode_audit <- analysis_all %>% group_by(cycle, begin_year) %>% summarise(
  n = n(), n_smq020_valid = sum(smq020 %in% c(0,1), na.rm = TRUE), n_smq040_valid = sum(smq040 %in% c(1,2,3), na.rm = TRUE),
  n_valid_start_age = sum(is.finite(smoking_start_age)), n_current_smoker = sum(current_smoker_baseline == 1, na.rm = TRUE),
```

```
  n_former_smoker = sum(former_smoker_baseline == 1, na.rm = TRUE),
n_smoking_at_diagnosis = sum(smoking_at_diagnosis == 1, na.rm = TRUE),
  n_postdiagnostic_quitter = sum(exposure_quit == 1, na.rm = TRUE),
n_persistent_smoker = sum(exposure_quit == 0, na.rm = TRUE), .groups = "drop")
safe_write_csv(recode_audit, file.path(DIRS$audit,
"02b_smoking_recode_audit_by_cycle.csv"))

build_flow_diagnostic <- function(dat) tibble(
  step = c("All records", paste0("Age >=", MIN_AGE), "Any cardiopulmonary disease",
"Valid diagnosis age",
           "Ever smoker with valid regular-smoking start age by diagnosis", "Smoking
at diagnosis", "Exposure classifiable",
           "Mortality linkage eligible and positive follow-up", "Valid weight/design
variables", "Final primary domain"),
  n = c(nrow(dat), sum(dat$age >= MIN_AGE, na.rm=TRUE),
        sum(dat$age >= MIN_AGE & dat$cardiopulmonary_any == 1, na.rm=TRUE),
        sum(dat$age >= MIN_AGE & dat$cardiopulmonary_any == 1 &
is.finite(dat$cp_dx_age), na.rm=TRUE),
        sum(dat$age >= MIN_AGE & dat$cardiopulmonary_any == 1 &
is.finite(dat$cp_dx_age) & dat$smoking_started_by_dx == 1, na.rm=TRUE),
        sum(dat$age >= MIN_AGE & dat$cardiopulmonary_any == 1 &
is.finite(dat$cp_dx_age) & dat$smoking_at_diagnosis == 1, na.rm=TRUE),
        sum(dat$age >= MIN_AGE & dat$cardiopulmonary_any == 1 &
is.finite(dat$cp_dx_age) & dat$smoking_at_diagnosis == 1 & dat$exposure_classifiable
== 1, na.rm=TRUE),
        sum(dat$age >= MIN_AGE & dat$cardiopulmonary_any == 1 &
is.finite(dat$cp_dx_age) & dat$smoking_at_diagnosis == 1 & dat$exposure_classifiable
== 1 & dat$eligstat == 1 & is.finite(dat$permth_int) & dat$permth_int > 0,
na.rm=TRUE),
        sum(dat$age >= MIN_AGE & dat$cardiopulmonary_any == 1 &
is.finite(dat$cp_dx_age) & dat$smoking_at_diagnosis == 1 & dat$exposure_classifiable
== 1 & dat$eligstat == 1 & is.finite(dat$permth_int) & dat$permth_int > 0 &
is.finite(dat$wt_int_20y) & dat$wt_int_20y > 0 & !is.na(dat$survey_psu_uid) &
!is.na(dat$survey_strata_uid), na.rm=TRUE),
        sum(dat$domain_primary == 1, na.rm=TRUE))
)
flow_check <- build_flow_diagnostic(analysis_all)
safe_write_csv(flow_check, file.path(DIRS$audit, "02c_reconstruction_flow.csv"))

primary_n_check <- sum(analysis_all$domain_primary == 1, na.rm = TRUE)
cat("  重建后主分析域样本量:", primary_n_check, "\n", sep = "")
if (sum(analysis_all$smq040 %in% c(1,2,3), na.rm = TRUE) < 1000) stop("SMQ040重编码
后有效人数异常少，请发送02a/02b审计文件。")
if (primary_n_check < 500) stop("主分析样本仍异常少（n=", primary_n_check, "）。请发送
02a、02b、02c审计文件，不要继续投稿。")

saveRDS(analysis_all, file.path(DIRS$processed, "02_analysis_all_derived.rds"),
compress = "xz")
safe_write_csv(head(analysis_all, 5000), file.path(DIRS$audit,
"02_analysis_preview_first5000.csv"))
```

```
# ==============================================================================
# 7. 权重、死亡代码、流程和一致性审计
# ==============================================================================

cat("[3/14] 进行权重、死亡代码和队列流程审计……\n")

weight_audit <- analysis_all %>%
  group_by(cycle, begin_year) %>%
  summarise(
    n = n(),
    n_wtint4 = sum(is.finite(wtint4yr) & wtint4yr > 0),
    n_wtint2 = sum(is.finite(wtint2yr) & wtint2yr > 0),
    n_wtmec4 = sum(is.finite(wtmec4yr) & wtmec4yr > 0),
    n_wtmec2 = sum(is.finite(wtmec2yr) & wtmec2yr > 0),
    n_wt_int_20y = sum(is.finite(wt_int_20y) & wt_int_20y > 0),
    sum_wt_int_20y = sum(wt_int_20y, na.rm = TRUE),
    n_wt_int_18y_sens = sum(is.finite(wt_int_18y_sens) & wt_int_18y_sens > 0),
    sum_wt_int_18y_sens = sum(wt_int_18y_sens, na.rm = TRUE),
    n_wt_int_16y = sum(is.finite(wt_int_16y) & wt_int_16y > 0),
    sum_wt_int_16y = sum(wt_int_16y, na.rm = TRUE),
    .groups = "drop"
  )
safe_write_csv(weight_audit, file.path(DIRS$audit, "03_weight_audit_by_cycle.csv"))

mortality_code_audit <- analysis_all %>%
  filter(eligstat == 1) %>%
  count(cycle, begin_year, mortstat, ucod_leading, name = "n") %>%
  arrange(begin_year, desc(n))
safe_write_csv(mortality_code_audit, file.path(DIRS$audit,
"04_mortality_code_audit_by_cycle.csv"))

variable_harmonization_audit <- analysis_all %>%
  group_by(cycle, begin_year) %>%
  summarise(
    n = n(),
    n_any_cardiopulmonary = sum(cardiopulmonary_any == 1, na.rm = TRUE),
    n_valid_diagnosis_age = sum(is.finite(cp_dx_age)),
    n_postdiagnostic_quitters = sum(exposure_quit == 1, na.rm = TRUE),
    n_quit_age_direct = sum(quit_age_source == "Direct SMD055", na.rm = TRUE),
    n_quit_age_derived = sum(quit_age_source == "Derived SMQ050Q/U", na.rm = TRUE),
    n_quit_duration_right_censored_50plus = sum(quit_time_q_raw == 66666, na.rm =
TRUE),
    median_direct_derived_abs_difference = median(quit_age_discrepancy_years, na.rm
= TRUE),
    n_current_cigarettes_per_day = sum(is.finite(cig_day_current)),
    .groups = "drop"
  ) %>%
  mutate(
    median_direct_derived_abs_difference = ifelse(
```

```
      is.nan(median_direct_derived_abs_difference), NA_real_,
median_direct_derived_abs_difference
    )
  )
safe_write_csv(variable_harmonization_audit,
               file.path(DIRS$audit, "04b_variable_harmonization_by_cycle.csv"))

linkage_audit <- analysis_all %>%
  group_by(cycle, begin_year) %>%
  summarise(
    n = n(),
    n_linkage_eligible = sum(eligstat == 1, na.rm = TRUE),
    linkage_eligible_percent = 100 * mean(eligstat == 1, na.rm = TRUE),
    n_positive_interview_followup = sum(eligstat == 1 & is.finite(permth_int) &
permth_int > 0, na.rm = TRUE),
    .groups = "drop"
  )
safe_write_csv(linkage_audit, file.path(DIRS$audit,
"04c_mortality_linkage_eligibility_by_cycle.csv"))

analytic_cycle_audit <- analysis_all %>%
  filter(domain_primary == 1) %>%
  group_by(cycle, begin_year) %>%
  summarise(
    n_primary = n(),
    n_postdiagnostic_quitters = sum(exposure_quit == 1, na.rm = TRUE),
    n_persistent_smokers = sum(exposure_quit == 0, na.rm = TRUE),
    all_cause_deaths = sum(event_allcause == 1, na.rm = TRUE),
    heart_disease_deaths = sum(event_heart == 1, na.rm = TRUE),
    median_followup_years = median(time_int_years, na.rm = TRUE),
    max_followup_years = safe_max(time_int_years),
    .groups = "drop"
  )
safe_write_csv(analytic_cycle_audit,
               file.path(DIRS$audit,
"04d_primary_analytic_sample_and_events_by_cycle.csv"))

cause_availability_note <- tibble(
  outcome = c(
    "All-cause mortality",
    "Heart-disease mortality (UCOD_LEADING=001)",
    "Cancer mortality (UCOD_LEADING=002)",
    "Cardiovascular composite mortality (001+005)",
    "Chronic lower respiratory disease mortality (003)"
  ),
  cycles_used = c(
    "1999–2018", "1999–2018", "1999–2018", "1999–2014", "1999–2014"
  ),
  reason = c(
    "MORTSTAT available in all included cycles",
```

```
    "001 is separately available for 2015–2018 NHANES in the public LMF",
    "002 is separately available for 2015–2018 NHANES in the public LMF",
    "005 is not separately available for 2015–2018 NHANES in the public LMF",
    "003 is not separately available for 2015–2018 NHANES in the public LMF"
  )
)
safe_write_csv(cause_availability_note, file.path(DIRS$audit,
"05_cause_of_death_availability_and_naming.csv"))

cohort_flow <- tibble(
  step = c(
    "All NHANES participants, 1999–2018",
    paste0("Age ≥", MIN_AGE, " years"),
    "Any prespecified cardiopulmonary disease",
    "Valid age at first cardiopulmonary diagnosis",
    "Ever smoker who started by diagnosis",
    "Classified as smoking at diagnosis",
    "Exposure classifiable: post-diagnostic quitter or persistent smoker",
    "Eligible for mortality linkage with positive interview follow-up",
    "Valid 20-year interview weight and survey design variables",
    "Final primary analytic domain"
  ),
  n = c(
    nrow(analysis_all),
    sum(analysis_all$age >= MIN_AGE, na.rm = TRUE),
    sum(analysis_all$age >= MIN_AGE & analysis_all$cardiopulmonary_any == 1, na.rm =
TRUE),
    sum(analysis_all$age >= MIN_AGE & analysis_all$cardiopulmonary_any == 1 &
is.finite(analysis_all$cp_dx_age), na.rm = TRUE),
    sum(analysis_all$age >= MIN_AGE & analysis_all$cardiopulmonary_any == 1 &
is.finite(analysis_all$cp_dx_age) & analysis_all$smoking_started_by_dx == 1, na.rm =
TRUE),
    sum(analysis_all$age >= MIN_AGE & analysis_all$cardiopulmonary_any == 1 &
is.finite(analysis_all$cp_dx_age) & analysis_all$smoking_at_diagnosis == 1, na.rm =
TRUE),
    sum(analysis_all$age >= MIN_AGE & analysis_all$cardiopulmonary_any == 1 &
is.finite(analysis_all$cp_dx_age) & analysis_all$smoking_at_diagnosis == 1 &
analysis_all$exposure_classifiable == 1, na.rm = TRUE),
    sum(analysis_all$age >= MIN_AGE & analysis_all$cardiopulmonary_any == 1 &
is.finite(analysis_all$cp_dx_age) & analysis_all$smoking_at_diagnosis == 1 &
analysis_all$exposure_classifiable == 1 & analysis_all$eligstat == 1 &
analysis_all$permth_int > 0, na.rm = TRUE),
    sum(analysis_all$domain_primary == 1, na.rm = TRUE),
    sum(analysis_all$domain_primary == 1, na.rm = TRUE)
  )
) %>%
  mutate(excluded_from_previous = c(NA_integer_, head(n, -1) - tail(n, -1)))
safe_write_csv(cohort_flow, file.path(DIRS$supp_tables, "Table_S1_cohort_flow.csv"))

qc_summary <- tibble(
```

```
  check = c(
    "Diagnosis age after baseline age",
    "Quit age before smoking-start age",
    "Simultaneously classified as quitter and persistent smoker",
    "Missing 4-year interview weight in 1999–2002 among interviewed adults",
    "Missing 4-year MEC weight in 1999–2002 among examined adults",
    "Direct and derived quit ages differ by >2 years where both exist",
    "Former smokers with right-censored quit duration >=50 years"
  ),
  n = c(
    sum(analysis_all$qc_dx_after_baseline == 1, na.rm = TRUE),
    sum(analysis_all$qc_quit_before_start == 1, na.rm = TRUE),
    sum(analysis_all$qc_both_exposure_groups == 1, na.rm = TRUE),
    sum(analysis_all$begin_year %in% c(1999, 2001) & analysis_all$age >= 18 &
!is.finite(analysis_all$wtint4yr), na.rm = TRUE),
    sum(analysis_all$begin_year %in% c(1999, 2001) & analysis_all$age >= 18 &
!is.finite(analysis_all$wtmec4yr), na.rm = TRUE),
    sum(analysis_all$quit_age_discrepancy_years > 2, na.rm = TRUE),
    sum(analysis_all$quit_time_q_raw == 66666, na.rm = TRUE)
  )
)
safe_write_csv(qc_summary, file.path(DIRS$audit, "06_core_qc_summary.csv"))

# ============================================================================
# 8. 建立复杂抽样设计对象
# ============================================================================

cat("[4/14] 建立NHANES复杂抽样设计对象……\n")

# 先在所有具有有效权重和死亡随访的成人中建立设计，再用subset定义研究子总体。
base_int_20 <- analysis_all %>%
  filter(
    age >= 18,
    eligstat == 1,
    is.finite(permth_int), permth_int > 0,
    is.finite(wt_int_20y), wt_int_20y > 0,
    is.finite(sdmvpsu), is.finite(sdmvstra)
  )

design_int_20_full <- survey::svydesign(
  ids = ~survey_psu_uid,
  strata = ~survey_strata_uid,
  weights = ~wt_int_20y,
  nest = TRUE,
  data = base_int_20
)
design_primary <- base::subset(design_int_20_full, domain_primary == 1)
design_primary$variables <- droplevels(design_primary$variables)

base_mec_20 <- analysis_all %>%
```

```
  filter(
    age >= 18,
    eligstat == 1,
    is.finite(permth_exm), permth_exm > 0,
    is.finite(wt_mec_20y), wt_mec_20y > 0,
    is.finite(sdmvpsu), is.finite(sdmvstra)
  )

design_mec_20_full <- survey::svydesign(
  ids = ~survey_psu_uid,
  strata = ~survey_strata_uid,
  weights = ~wt_mec_20y,
  nest = TRUE,
  data = base_mec_20
)
design_mec_extended <- base::subset(design_mec_20_full, domain_mec_extended == 1)
design_mec_extended$variables <- droplevels(design_mec_extended$variables)

base_int_18_sens <- analysis_all %>%
  filter(
    begin_year <= 2015,
    age >= 18,
    eligstat == 1,
    is.finite(permth_int), permth_int > 0,
    is.finite(wt_int_18y_sens), wt_int_18y_sens > 0,
    is.finite(sdmvpsu), is.finite(sdmvstra)
  )

design_int_18_sens_full <- survey::svydesign(
  ids = ~survey_psu_uid,
  strata = ~survey_strata_uid,
  weights = ~wt_int_18y_sens,
  nest = TRUE,
  data = base_int_18_sens
)
design_18_sens <- base::subset(design_int_18_sens_full, domain_18y_sens == 1)
design_18_sens$variables <- droplevels(design_18_sens$variables)

base_int_16 <- analysis_all %>%
  filter(
    begin_year <= 2013,
    age >= 18,
    eligstat == 1,
    is.finite(permth_int), permth_int > 0,
    is.finite(wt_int_16y), wt_int_16y > 0,
    is.finite(sdmvpsu), is.finite(sdmvstra)
  )

design_int_16_full <- survey::svydesign(
  ids = ~survey_psu_uid,
```

```
  strata = ~survey_strata_uid,
  weights = ~wt_int_16y,
  nest = TRUE,
  data = base_int_16
)
design_16 <- base::subset(design_int_16_full, domain_16y == 1)
design_16$variables <- droplevels(design_16$variables)

primary_df <- design_primary$variables %>% droplevels()
saveRDS(primary_df, file.path(DIRS$processed, "03_primary_analysis_dataset.rds"),
compress = "xz")

analysis_summary <- tibble(
  item = c(
    "Primary analysis n",
    "Post-diagnostic quitters",
    "Persistent smokers",
    "All-cause deaths",
    "Heart-disease deaths (001)",
    "Cancer deaths (002)",
    "1999–2016 sensitivity-analysis n",
    "1999–2014 cardiovascular composite deaths (001+005)",
    "1999–2014 chronic lower respiratory deaths (003)",
    "Median interview follow-up, years",
    "Survey design degrees of freedom, primary domain"
  ),
  value = c(
    nrow(primary_df),
    sum(primary_df$exposure_quit == 1),
    sum(primary_df$exposure_quit == 0),
    sum(primary_df$event_allcause == 1),
    sum(primary_df$event_heart == 1),
    sum(primary_df$event_cancer == 1),
    nrow(design_18_sens$variables),
    sum(design_16$variables$event_cvd_composite_16y == 1, na.rm = TRUE),
    sum(design_16$variables$event_clrd_16y == 1, na.rm = TRUE),
    median(primary_df$time_int_years, na.rm = TRUE),
    survey::degf(design_primary)
  )
)
safe_write_csv(analysis_summary, file.path(DIRS$results, "00_analysis_summary.csv"))

# ===============================================================================
# 9. 基线特征和缺失情况
# ===============================================================================

cat("[5/14] 输出基线特征、缺失和事件构成……\n")

continuous_vars <- c(
  "age", "survey_midyear", "years_since_dx", "pre_dx_smoking_years", "pir",
```

```
  "cig_per_day_proxy", "pack_years_proxy", "bmi"
)

baseline_cont <- map_dfr(continuous_vars, function(v) {
  map_dfr(c(0, 1), function(g) {
    sub <- primary_df %>% filter(exposure_quit == g)
    tibble(
      variable = v,
      group = ifelse(g == 1, "Post-diagnostic quitter", "Persistent smoker"),
      n_nonmissing = sum(is.finite(safe_numeric(sub[[v]]))),
      mean_weighted = weighted_mean_safe(sub[[v]], sub$wt_int_20y),
      sd_weighted = weighted_sd_safe(sub[[v]], sub$wt_int_20y),
      median_unweighted = safe_quantile(sub[[v]], 0.50),
      q1_unweighted = safe_quantile(sub[[v]], 0.25),
      q3_unweighted = safe_quantile(sub[[v]], 0.75)
    )
  })
}) %>%
  mutate(
    weighted_mean_sd = sprintf("%.2f ± %.2f", mean_weighted, sd_weighted),
    unweighted_median_iqr = sprintf("%.2f (%.2f–%.2f)", median_unweighted,
q1_unweighted, q3_unweighted)
  )
safe_write_csv(baseline_cont, file.path(DIRS$supp_tables,
"Table_S2_baseline_continuous_source.csv"))

categorical_vars <- c(
  "sex_f", "race_f", "education_f", "disease_f",
  "diabetes_f", "hypertension_f", "age_group_f"
)

baseline_cat <- map_dfr(categorical_vars, function(v) {
  levs <- levels(factor(primary_df[[v]]))
  map_dfr(levs, function(lv) {
    map_dfr(c(0, 1), function(g) {
      sub <- primary_df %>% filter(exposure_quit == g)
      n_raw <- sum(sub[[v]] == lv, na.rm = TRUE)
      p_w <- weighted_prop_safe(sub[[v]], sub$wt_int_20y, lv)
      tibble(
        variable = v,
        level = lv,
        group = ifelse(g == 1, "Post-diagnostic quitter", "Persistent smoker"),
        n = n_raw,
        weighted_percent = 100 * p_w,
        n_weighted_percent = sprintf("%d (%.1f%%)", n_raw, 100 * p_w)
      )
    })
  })
})
safe_write_csv(baseline_cat, file.path(DIRS$supp_tables,
```

```
"Table_S3_baseline_categorical_source.csv"))

missingness <- tibble(
  variable = c(
    continuous_vars, categorical_vars,
    "time_int_years", "event_allcause", "event_heart", "event_cancer",
    "wt_int_20y", "sdmvpsu", "sdmvstra"
  )
) %>%
  mutate(
    n_total = nrow(primary_df),
    n_missing = map_int(variable, ~ sum(is.na(primary_df[[.x]]))),
    missing_percent = 100 * n_missing / n_total
  ) %>%
  arrange(desc(missing_percent))
safe_write_csv(missingness, file.path(DIRS$audit,
"07_missingness_primary_dataset.csv"))

outcome_counts <- tibble(
  outcome = c(
    "All-cause mortality",
    "Heart-disease mortality (001)",
    "Cancer mortality (002)"
  ),
  cycles = "1999–2018",
  events = c(
    sum(primary_df$event_allcause == 1),
    sum(primary_df$event_heart == 1),
    sum(primary_df$event_cancer == 1)
  ),
  n = nrow(primary_df)
) %>%
  bind_rows(tibble(
    outcome = c(
      "Cardiovascular composite mortality (001+005)",
      "Chronic lower respiratory disease mortality (003)"
    ),
    cycles = "1999–2014",
    events = c(
      sum(design_16$variables$event_cvd_composite_16y == 1, na.rm = TRUE),
      sum(design_16$variables$event_clrd_16y == 1, na.rm = TRUE)
    ),
    n = nrow(design_16$variables)
  ))
safe_write_csv(outcome_counts, file.path(DIRS$supp_tables,
"Table_S4_outcome_counts_and_cycles.csv"))

# ============================================================================
# 10. 主模型和次要结局模型
# ============================================================================
```

```
cat("[6/14] 运行复杂抽样Cox主模型……\n")

RHS_MODELS <- list(
  M0_unadjusted = "exposure_quit",
  M1_demographic = "exposure_quit + age10 + sex_f + race_f + education_f",
  M2_core = paste(
    "exposure_quit + age10 + sex_f + race_f + education_f +",
    "disease_f + years_since_dx5 + pre_dx_smoking_years10 +
survey_cycle_2y_centered"
  ),
  M2_core_no_calendar = paste(
    "exposure_quit + age10 + sex_f + race_f + education_f +",
    "disease_f + years_since_dx5 + pre_dx_smoking_years10"
  )
)

main_outcomes <- tibble::tribble(
  ~event_var,       ~outcome_label,
  "event_allcause", "All-cause mortality",
  "event_heart",    "Heart-disease mortality",
  "event_cancer",   "Cancer mortality"
)

main_models <- list()
for (k in seq_len(nrow(main_outcomes))) {
  ev <- main_outcomes$event_var[k]
  ol <- main_outcomes$outcome_label[k]
  for (nm in c("M0_unadjusted", "M1_demographic", "M2_core")) {
    main_models[[length(main_models) + 1]] <- run_svycox(
      design = design_primary,
      time_var = "time_int_years",
      event_var = ev,
      rhs = RHS_MODELS[[nm]],
      analysis_label = paste0("Primary 1999-2018 / ", nm),
      outcome_label = ol
    )
  }
}
main_models_df <- bind_rows(main_models) %>%
  mutate(HR_95CI = format_hr(HR, CI_low, CI_high), p_formatted = format_p(p))
safe_write_csv(main_models_df, file.path(DIRS$results,
"MODEL_primary_survey_weighted_cox_models.csv"))

# 1999-2014可识别的心血管复合与慢性下呼吸道死亡。
secondary_16_models <- bind_rows(
  run_svycox(
    design_16, "time_int_years", "event_cvd_composite_16y", RHS_MODELS$M2_core,
    "Secondary restricted 1999-2014 / M2_core",
    "Cardiovascular composite mortality (heart + cerebrovascular)"
```

```
  ),
  run_svycox(
    design_16, "time_int_years", "event_clrd_16y", RHS_MODELS$M2_core,
    "Secondary restricted 1999–2014 / M2_core",
    "Chronic lower respiratory disease mortality"
  )
) %>%
  mutate(HR_95CI = format_hr(HR, CI_low, CI_high), p_formatted = format_p(p))
safe_write_csv(secondary_16_models, file.path(DIRS$results,
"MODEL_secondary_1999_2014_cause_specific_models.csv"))

# E-value只针对主结局M2_core（若有稳定估计）。
evalue_results <- main_models_df %>%
  filter(grepl("M2_core$", analysis), outcome %in% c("All-cause mortality",
"Heart-disease mortality")) %>%
  rowwise() %>%
  mutate(evalue_tmp = list(manual_evalue(HR, CI_low, CI_high))) %>%
  unnest_wider(evalue_tmp) %>%
  ungroup()
safe_write_csv(evalue_results, file.path(DIRS$results,
"01_E_values_primary_M2.csv"))

# ===============================================================================
# 11. 敏感性分析：严格戒烟、Landmark、MEC扩展、吸烟强度、疾病限制
# ===============================================================================

cat("[7/14] 运行landmark及其他敏感性分析……\n")

sensitivity_results <- list()

# 11.0a 不调整调查周期，核查日历时间项对结果的影响。
for (ev in c("event_allcause", "event_heart")) {
  sensitivity_results[[length(sensitivity_results) + 1]] <- run_svycox(
    design_primary, "time_int_years", ev, RHS_MODELS$M2_core_no_calendar,
    "Primary 1999–2018 without calendar-cycle adjustment",
    ifelse(ev == "event_allcause", "All-cause mortality", "Heart-disease mortality")
  )
}

# 11.0b 排除2017–2018：去除直接SMD055结构性缺失的周期，并评估较短随访的影响。
for (ev in c("event_allcause", "event_heart")) {
  sensitivity_results[[length(sensitivity_results) + 1]] <- run_svycox(
    design_18_sens, "time_int_years", ev, RHS_MODELS$M2_core,
    "Restricted 1999–2016 with 18-year combined interview weights",
    ifelse(ev == "event_allcause", "All-cause mortality", "Heart-disease mortality")
  )
}

# 11.1 严格定义：戒烟年龄必须大于诊断年龄，而不是同岁。
design_strict <- base::subset(design_primary, strict_quit_definition == 1)
```

```
for (ev in c("event_allcause", "event_heart")) {
  sensitivity_results[[length(sensitivity_results) + 1]] <- run_svycox(
    design_strict, "time_int_years", ev, RHS_MODELS$M2_core,
    "Strict exposure: quit age > diagnosis age",
    ifelse(ev == "event_allcause", "All-cause mortality", "Heart-disease mortality")
  )
}

# 11.2 Landmark:必须随访超过landmark,并从landmark后重新计时。
landmark_results <- list()
for (m in LANDMARK_MONTHS) {
  time_name <- paste0("time_after_landmark_", m, "m")
  design_lm <- design_primary
  design_lm$variables[[time_name]] <- (design_lm$variables$permth_int - m) / 12
  design_lm$variables$eligible_landmark_internal <- design_lm$variables$permth_int >
m
  design_lm <- base::subset(design_lm, eligible_landmark_internal)
  design_lm$variables <- droplevels(design_lm$variables)

  for (ev in c("event_allcause", "event_heart")) {
    landmark_results[[length(landmark_results) + 1]] <- run_svycox(
      design_lm,
      time_name,
      ev,
      RHS_MODELS$M2_core,
      paste0("Landmark at ", m, " months / M2_core"),
      ifelse(ev == "event_allcause", "All-cause mortality", "Heart-disease
mortality")
    ) %>% mutate(landmark_months = m)
  }
}
landmark_results_df <- bind_rows(landmark_results) %>%
  mutate(HR_95CI = format_hr(HR, CI_low, CI_high), p_formatted = format_p(p))
safe_write_csv(landmark_results_df, file.path(DIRS$results,
"MODEL_landmark_analyses_corrected.csv"))

# 11.3 MEC/BMI扩展模型:对应使用MEC权重和从MEC日期起算的随访时间。
rhs_mec_extended <- paste(
  RHS_MODELS$M2_core,
  "+ bmi5 + pir + diabetes_f + hypertension_f"
)
for (ev in c("event_allcause", "event_heart")) {
  sensitivity_results[[length(sensitivity_results) + 1]] <- run_svycox(
    design_mec_extended,
    "time_exm_years",
    ev,
    rhs_mec_extended,
    "MEC-weighted extended complete-case model",
    ifelse(ev == "event_allcause", "All-cause mortality", "Heart-disease mortality")
  )
```

```
}

# 11.4 吸烟强度代理完整病例敏感性;明确不是主模型。
rhs_smoking_burden <- paste(RHS_MODELS$M2_core, "+ pack_years10")
for (ev in c("event_allcause", "event_heart")) {
  sensitivity_results[[length(sensitivity_results) + 1]] <- run_svycox(
    design_primary,
    "time_int_years",
    ev,
    rhs_smoking_burden,
    "Smoking-burden proxy complete-case sensitivity",
    ifelse(ev == "event_allcause", "All-cause mortality", "Heart-disease mortality")
  )
}

# 11.5 限制在心血管病或慢性肺病人群。
design_cvd_only <- base::subset(design_primary, cvd_any == 1)
design_lung_only <- base::subset(design_primary, lung_any == 1)
for (ev in c("event_allcause", "event_heart")) {
  sensitivity_results[[length(sensitivity_results) + 1]] <- run_svycox(
    design_cvd_only, "time_int_years", ev,
    "exposure_quit + age10 + sex_f + race_f + education_f + years_since_dx5 +
pre_dx_smoking_years10 + survey_cycle_2y_centered",
    "Restricted to participants with cardiovascular disease",
    ifelse(ev == "event_allcause", "All-cause mortality", "Heart-disease mortality")
  )
  sensitivity_results[[length(sensitivity_results) + 1]] <- run_svycox(
    design_lung_only, "time_int_years", ev,
    "exposure_quit + age10 + sex_f + race_f + education_f + years_since_dx5 +
pre_dx_smoking_years10 + survey_cycle_2y_centered",
    "Restricted to participants with chronic lung disease",
    ifelse(ev == "event_allcause", "All-cause mortality", "Heart-disease mortality")
  )
}

sensitivity_results_df <- bind_rows(sensitivity_results, landmark_results_df) %>%
  mutate(HR_95CI = format_hr(HR, CI_low, CI_high), p_formatted = format_p(p))
safe_write_csv(sensitivity_results_df, file.path(DIRS$results,
"MODEL_sensitivity_analyses.csv"))

# ============================================================================
# 12. 倾向评分重叠加权(支持性,不取代主设计型回归)
# ============================================================================

cat("[8/14] 运行支持性的倾向评分重叠加权分析……\n")

ps_formula <- exposure_quit ~ age10 + sex_f + race_f + education_f +
  disease_f + years_since_dx5 + pre_dx_smoking_years10 + survey_cycle_2y_centered

ps_needed <- unique(c("exposure_quit", all.vars(ps_formula)))
```

```
ps_design <- complete_design(design_primary, ps_needed)

ps_fit <- tryCatch(
  survey::svyglm(
    ps_formula,
    design = ps_design,
    family = quasibinomial(),
    control = glm.control(maxit = 100)
  ),
  error = function(e) NULL
)

if (is.null(ps_fit)) {
  overlap_results <- tibble(
    analysis = "Survey × overlap-weighted supportive analysis",
    outcome = c("All-cause mortality", "Heart-disease mortality"),
    status = "failed",
    error = "Survey-weighted propensity-score model failed"
  )
  balance_results <- tibble(note = "Propensity-score model failed; balance not
calculated.")
  primary_ps <- ps_design$variables
} else {
  primary_ps <- ps_design$variables %>%
    mutate(
      ps_quit = pmin(pmax(as.numeric(stats::predict(ps_fit, type = "response")),
0.01), 0.99),
      overlap_factor = ifelse(exposure_quit == 1, 1 - ps_quit, ps_quit),
      weight_overlap_combined = wt_int_20y * overlap_factor
    )

  overlap_design <- survey::svydesign(
    ids = ~survey_psu_uid,
    strata = ~survey_strata_uid,
    weights = ~weight_overlap_combined,
    nest = TRUE,
    data = primary_ps
  )

  overlap_results <- bind_rows(
    run_svycox(
      overlap_design, "time_int_years", "event_allcause", "exposure_quit",
      "Survey × overlap-weighted supportive analysis",
      "All-cause mortality"
    ),
    run_svycox(
      overlap_design, "time_int_years", "event_heart", "exposure_quit",
      "Survey × overlap-weighted supportive analysis",
      "Heart-disease mortality"
    ),
```

```
    run_svycox(
      overlap_design, "time_int_years", "event_allcause", RHS_MODELS$M2_core,
      "Survey × overlap-weighted doubly adjusted sensitivity",
      "All-cause mortality"
    ),
    run_svycox(
      overlap_design, "time_int_years", "event_heart", RHS_MODELS$M2_core,
      "Survey × overlap-weighted doubly adjusted sensitivity",
      "Heart-disease mortality"
    )
  ) %>%
    mutate(HR_95CI = format_hr(HR, CI_low, CI_high), p_formatted = format_p(p))

  balance_cont_vars <- c("age", "survey_midyear", "years_since_dx",
"pre_dx_smoking_years", "pir")
  balance_cat_vars <- c("sex_f", "race_f", "education_f", "disease_f")

  balance_cont <- map_dfr(balance_cont_vars, function(v) {
    tibble(
      variable = v,
      level = NA_character_,
      type = "continuous",
      smd_survey = smd_continuous(primary_ps[[v]], primary_ps$exposure_quit,
primary_ps$wt_int_20y),
      smd_overlap = smd_continuous(primary_ps[[v]], primary_ps$exposure_quit,
primary_ps$weight_overlap_combined)
    )
  })

  balance_cat <- map_dfr(balance_cat_vars, function(v) {
    map_dfr(levels(factor(primary_ps[[v]])), function(lv) {
      tibble(
        variable = v,
        level = lv,
        type = "categorical_level",
        smd_survey = smd_binary_level(primary_ps[[v]], primary_ps$exposure_quit,
primary_ps$wt_int_20y, lv),
        smd_overlap = smd_binary_level(primary_ps[[v]], primary_ps$exposure_quit,
primary_ps$weight_overlap_combined, lv)
      )
    })
  })

  balance_results <- bind_rows(balance_cont, balance_cat) %>%
    mutate(
      label = ifelse(is.na(level), variable, paste0(variable, ": ", level)),
      abs_smd_survey = abs(smd_survey),
      abs_smd_overlap = abs(smd_overlap)
    ) %>%
    arrange(desc(abs_smd_survey))
```

```
}

safe_write_csv(overlap_results, file.path(DIRS$results,
"MODEL_overlap_weighted_supportive_models.csv"))
safe_write_csv(balance_results, file.path(DIRS$results,
"02_covariate_balance_before_after_overlap.csv"))
saveRDS(primary_ps, file.path(DIRS$processed,
"04_primary_dataset_with_propensity_scores.rds"), compress = "xz")

# ==============================================================================
# 13. 预设亚组和交互作用
# ==============================================================================

cat("[9/14] 运行预设亚组与交互作用分析……\n")

subgroup_specs <- list(
  age_group_f = list(
    display = "Age group",
    strat_rhs = "exposure_quit + age10 + sex_f + race_f + education_f + disease_f +
years_since_dx5 + pre_dx_smoking_years10 + survey_cycle_2y_centered",
    interaction_rhs = "exposure_quit * age_group_f + age10 + sex_f + race_f +
education_f + disease_f + years_since_dx5 + pre_dx_smoking_years10 +
survey_cycle_2y_centered"
  ),
  sex_f = list(
    display = "Sex",
    strat_rhs = "exposure_quit + age10 + race_f + education_f + disease_f +
years_since_dx5 + pre_dx_smoking_years10 + survey_cycle_2y_centered",
    interaction_rhs = "exposure_quit * sex_f + age10 + race_f + education_f +
disease_f + years_since_dx5 + pre_dx_smoking_years10 + survey_cycle_2y_centered"
  ),
  disease_f = list(
    display = "Disease phenotype",
    strat_rhs = "exposure_quit + age10 + sex_f + race_f + education_f +
years_since_dx5 + pre_dx_smoking_years10 + survey_cycle_2y_centered",
    interaction_rhs = "exposure_quit * disease_f + age10 + sex_f + race_f +
education_f + years_since_dx5 + pre_dx_smoking_years10 + survey_cycle_2y_centered"
  )
)

subgroup_results <- list()
interaction_results <- list()

for (sg in names(subgroup_specs)) {
  spec <- subgroup_specs[[sg]]
  levels_sg <- levels(factor(design_primary$variables[[sg]]))

  for (lv in levels_sg) {
    des_sub <- design_primary
    des_sub$variables$.sg_internal <- des_sub$variables[[sg]] == lv
```

```
    des_sub <- base::subset(des_sub, .sg_internal)
    des_sub$variables <- droplevels(des_sub$variables)

    n_sub <- nrow(des_sub$variables)
    events_sub <- sum(des_sub$variables$event_heart == 1, na.rm = TRUE)
    n_q <- sum(des_sub$variables$exposure_quit == 1, na.rm = TRUE)
    n_p <- sum(des_sub$variables$exposure_quit == 0, na.rm = TRUE)

    if (events_sub >= MIN_EVENTS_SUBGROUP && n_q >= 20 && n_p >= 20) {
      one <- run_svycox(
        des_sub, "time_int_years", "event_heart", spec$strat_rhs,
        paste0("Subgroup: ", spec$display, " = ", lv),
        "Heart-disease mortality"
      )
    } else {
      one <- tibble(
        analysis = paste0("Subgroup: ", spec$display, " = ", lv),
        outcome = "Heart-disease mortality",
        time_variable = "time_int_years",
        event_variable = "event_heart",
        formula_rhs = spec$strat_rhs,
        model_type = "survey::svycoxph",
        n = n_sub,
        events = events_sub,
        n_quit = n_q,
        n_persistent = n_p,
        design_df = tryCatch(survey::degf(des_sub), error = function(e) NA_real_),
        term = "exposure_quit",
        logHR = NA_real_, SE = NA_real_, HR = NA_real_, CI_low = NA_real_, CI_high =
NA_real_, p = NA_real_,
        status = "not_run", error = "亚组事件数或两组样本不足"
      )
    }
    one$subgroup_variable <- sg
    one$subgroup_label <- spec$display
    one$subgroup_level <- lv
    subgroup_results[[length(subgroup_results) + 1]] <- one
  }

  # 全样本交互作用检验。疾病表型交互作用排除仅1例且无事件的Other水平，
  # 避免无信息水平导致信息矩阵奇异。
  des_int_source <- design_primary
  if (sg == "disease_f") {
    des_int_source$variables$.interaction_keep_internal <-
      des_int_source$variables$disease_f != "Other"
    des_int_source <- base::subset(
      des_int_source,
      .interaction_keep_internal
    )
    des_int_source$variables <- droplevels(des_int_source$variables)
```

```
  }
  needed_int <- unique(c("time_int_years", "event_heart",
all.vars(as.formula(paste0("~", spec$interaction_rhs)))))
  des_int <- complete_design(des_int_source, needed_int)
  fml_int <- as.formula(paste0("Surv(time_int_years, event_heart) ~ ",
spec$interaction_rhs))

  int_row <- tryCatch({
    fit_int <- suppressWarnings(survey::svycoxph(fml_int, design = des_int))
    tibble(
      subgroup_variable = sg,
      subgroup_label = spec$display,
      interaction_p = wald_interaction_p(fit_int, sg),
      n = nrow(des_int$variables),
      events = sum(des_int$variables$event_heart == 1, na.rm = TRUE),
      status = "ok",
      error = NA_character_
    )
  }, error = function(e) {
    tibble(
      subgroup_variable = sg,
      subgroup_label = spec$display,
      interaction_p = NA_real_,
      n = nrow(des_int$variables),
      events = sum(des_int$variables$event_heart == 1, na.rm = TRUE),
      status = "failed",
      error = conditionMessage(e)
    )
  })
  interaction_results[[length(interaction_results) + 1]] <- int_row
}

subgroup_results_df <- bind_rows(subgroup_results) %>%
  mutate(HR_95CI = format_hr(HR, CI_low, CI_high), p_formatted = format_p(p))
interaction_results_df <- bind_rows(interaction_results) %>%
  mutate(interaction_p_formatted = format_p(interaction_p))

safe_write_csv(subgroup_results_df, file.path(DIRS$results,
"MODEL_prespecified_subgroup_estimates.csv"))
safe_write_csv(interaction_results_df, file.path(DIRS$results,
"MODEL_interaction_tests.csv"))


# ============================================================================
# 14. 戒烟年龄一致性敏感性分析与比例风险假设检查
# ============================================================================

cat("[10/14] 运行戒烟年龄一致性敏感性分析和比例风险假设检查……\n")

# ----------------------------------------------------------------------------
```

```
# 14.1 戒烟年龄直接值与推导值的一致性
# ------------------------------------------------------------------------------

design_primary$variables <- design_primary$variables %>%
  mutate(
    both_quit_ages_available =
      exposure_quit == 1 &
      is.finite(quit_age_direct) &
      is.finite(quit_age_derived),
    discordant_gt1 =
      both_quit_ages_available & quit_age_discrepancy_years > 1,
    discordant_gt2 =
      both_quit_ages_available & quit_age_discrepancy_years > 2,
    discordant_gt5 =
      both_quit_ages_available & quit_age_discrepancy_years > 5,
    keep_discrepancy_1 = !dplyr::coalesce(discordant_gt1, FALSE),
    keep_discrepancy_2 = !dplyr::coalesce(discordant_gt2, FALSE),
    keep_discrepancy_5 = !dplyr::coalesce(discordant_gt5, FALSE),
    keep_direct_quit_age_only =
      exposure_quit == 0 |
      (exposure_quit == 1 & quit_age_source == "Direct SMD055")
  )

design_primary$variables <- droplevels(design_primary$variables)
primary_df <- design_primary$variables

quit_age_discrepancy_distribution <- primary_df %>%
  filter(both_quit_ages_available) %>%
  summarise(
    n_with_both_sources = n(),
    mean_difference_years = mean(quit_age_discrepancy_years, na.rm = TRUE),
    sd_difference_years = sd(quit_age_discrepancy_years, na.rm = TRUE),
    median_difference_years = median(quit_age_discrepancy_years, na.rm = TRUE),
    p25_difference_years = quantile(quit_age_discrepancy_years, 0.25, na.rm = TRUE,
names = FALSE),
    p75_difference_years = quantile(quit_age_discrepancy_years, 0.75, na.rm = TRUE,
names = FALSE),
    p90_difference_years = quantile(quit_age_discrepancy_years, 0.90, na.rm = TRUE,
names = FALSE),
    max_difference_years = max(quit_age_discrepancy_years, na.rm = TRUE),
    n_gt1 = sum(discordant_gt1, na.rm = TRUE),
    n_gt2 = sum(discordant_gt2, na.rm = TRUE),
    n_gt5 = sum(discordant_gt5, na.rm = TRUE)
  )

safe_write_csv(
  quit_age_discrepancy_distribution,
  file.path(DIRS$supp_tables, "Table_S5_quit_age_discrepancy_distribution.csv")
)
```

```
quit_age_count_list <- list()
quit_age_model_list <- list()

for (threshold in c(1, 2, 5)) {
  keep_var <- paste0("keep_discrepancy_", threshold)
  discord_var <- paste0("discordant_gt", threshold)

  des_threshold <- design_primary
  des_threshold$variables$.keep_internal <-
    des_threshold$variables[[keep_var]]
  des_threshold <- base::subset(des_threshold, .keep_internal)
  des_threshold$variables <- droplevels(des_threshold$variables)

  quit_age_count_list[[as.character(threshold)]] <- tibble(
    analysis = paste0("Exclude direct-derived quit-age discrepancy >", threshold, "
years"),
    threshold_years = threshold,
    original_n = nrow(primary_df),
    original_quitters = sum(primary_df$exposure_quit == 1),
    quitters_with_both_sources = sum(primary_df$both_quit_ages_available, na.rm =
TRUE),
    discordant_quitters_excluded = sum(primary_df[[discord_var]], na.rm = TRUE),
    retained_n = nrow(des_threshold$variables),
    retained_quitters = sum(des_threshold$variables$exposure_quit == 1),
    retained_persistent_smokers = sum(des_threshold$variables$exposure_quit == 0),
    allcause_deaths = sum(des_threshold$variables$event_allcause == 1),
    heart_deaths = sum(des_threshold$variables$event_heart == 1),
    cancer_deaths = sum(des_threshold$variables$event_cancer == 1)
  )

  for (k in seq_len(nrow(main_outcomes))) {
    quit_age_model_list[[length(quit_age_model_list) + 1]] <- run_svycox(
      design = des_threshold,
      time_var = "time_int_years",
      event_var = main_outcomes$event_var[k],
      rhs = RHS_MODELS$M2_core,
      analysis_label = paste0("Quit-age discrepancy exclusion >", threshold, "
years"),
      outcome_label = main_outcomes$outcome_label[k]
    ) %>%
      mutate(
        sensitivity_type = "Quit-age discrepancy exclusion",
        threshold_years = threshold
      )
  }
}

# 最严格的来源分析:持续吸烟者全部保留;戒烟者仅保留直接SMD055戒烟年龄。
design_direct_quit_age <- design_primary
design_direct_quit_age$variables$.keep_direct_internal <-
```

```
  design_direct_quit_age$variables$keep_direct_quit_age_only
design_direct_quit_age <- base::subset(
  design_direct_quit_age,
  .keep_direct_internal
)
design_direct_quit_age$variables <- droplevels(design_direct_quit_age$variables)

quit_age_direct_count <- tibble(
  analysis = "Quitters restricted to directly reported SMD055 quit age",
  original_n = nrow(primary_df),
  original_quitters = sum(primary_df$exposure_quit == 1),
  retained_n = nrow(design_direct_quit_age$variables),
  retained_quitters = sum(design_direct_quit_age$variables$exposure_quit == 1),
  retained_persistent_smokers = sum(design_direct_quit_age$variables$exposure_quit
== 0),
  excluded_quitters_without_direct_age =
    sum(primary_df$exposure_quit == 1) -
    sum(design_direct_quit_age$variables$exposure_quit == 1),
  allcause_deaths = sum(design_direct_quit_age$variables$event_allcause == 1),
  heart_deaths = sum(design_direct_quit_age$variables$event_heart == 1),
  cancer_deaths = sum(design_direct_quit_age$variables$event_cancer == 1)
)

for (k in seq_len(nrow(main_outcomes))) {
  quit_age_model_list[[length(quit_age_model_list) + 1]] <- run_svycox(
    design = design_direct_quit_age,
    time_var = "time_int_years",
    event_var = main_outcomes$event_var[k],
    rhs = RHS_MODELS$M2_core,
    analysis_label = "Quitters restricted to directly reported SMD055 quit age",
    outcome_label = main_outcomes$outcome_label[k]
  ) %>%
    mutate(
      sensitivity_type = "Direct quit-age source only",
      threshold_years = NA_real_
    )
}

quit_age_counts_df <- bind_rows(
  bind_rows(quit_age_count_list),
  quit_age_direct_count
)
quit_age_sensitivity_models <- bind_rows(quit_age_model_list) %>%
  mutate(
    HR_95CI = format_hr(HR, CI_low, CI_high),
    p_formatted = format_p(p)
  )

safe_write_csv(
  quit_age_counts_df,
```

```
  file.path(DIRS$supp_tables, "Table_S6_quit_age_sensitivity_sample_counts.csv")
)
safe_write_csv(
  quit_age_sensitivity_models,
  file.path(DIRS$results, "MODEL_quit_age_sensitivity_models.csv")
)

quit_age_by_cycle <- primary_df %>%
  filter(exposure_quit == 1) %>%
  group_by(cycle, begin_year) %>%
  summarise(
    n_quitters = n(),
    n_direct_age = sum(is.finite(quit_age_direct)),
    n_derived_age = sum(is.finite(quit_age_derived)),
    n_both_sources = sum(both_quit_ages_available),
    n_discrepancy_gt1 = sum(discordant_gt1),
    n_discrepancy_gt2 = sum(discordant_gt2),
    n_discrepancy_gt5 = sum(discordant_gt5),
    median_discrepancy_years = ifelse(
      sum(both_quit_ages_available) > 0,
      median(quit_age_discrepancy_years[both_quit_ages_available], na.rm = TRUE),
      NA_real_
    ),
    .groups = "drop"
  )
safe_write_csv(
  quit_age_by_cycle,
  file.path(DIRS$supp_tables, "Table_S7_quit_age_discrepancy_by_cycle.csv")
)

# ------------------------------------------------------------------------------
# 14.2 比例风险假设检查
# ------------------------------------------------------------------------------

extract_zph_table <- function(zph, outcome_label, n_model, events) {
  tb <- as.data.frame(zph$table)
  tb$term <- rownames(tb)
  rownames(tb) <- NULL
  tibble(
    outcome = outcome_label,
    n = n_model,
    events = events,
    term = tb$term,
    chisq = if ("chisq" %in% names(tb)) tb$chisq else NA_real_,
    df = if ("df" %in% names(tb)) tb$df else NA_real_,
    p = if ("p" %in% names(tb)) tb$p else NA_real_,
    status = "ok",
    error = NA_character_
  )
}
```

```
ph_full_list <- list()

for (k in seq_len(nrow(main_outcomes))) {
  ev <- main_outcomes$event_var[k]
  ol <- main_outcomes$outcome_label[k]
  needed <- unique(c(
    "time_int_years",
    ev,
    all.vars(as.formula(paste0("~", RHS_MODELS$M2_core)))
  ))
  des_ph <- complete_design(design_primary, needed)
  fml_ph <- as.formula(
    paste0("survival::Surv(time_int_years, ", ev, ") ~ ", RHS_MODELS$M2_core)
  )

  fit_ph <- tryCatch(
    suppressWarnings(
      survey::svycoxph(
        fml_ph,
        design = des_ph,
        x = TRUE,
        y = TRUE,
        model = TRUE
      )
    ),
    error = function(e) e
  )

  if (inherits(fit_ph, "error")) {
    ph_full_list[[length(ph_full_list) + 1]] <- tibble(
      outcome = ol,
      n = nrow(des_ph$variables),
      events = sum(des_ph$variables[[ev]] == 1),
      term = NA_character_,
      chisq = NA_real_,
      df = NA_real_,
      p = NA_real_,
      status = "failed",
      error = conditionMessage(fit_ph)
    )
  } else {
    zph <- tryCatch(
      survival::cox.zph(
        fit_ph,
        transform = "km",
        terms = TRUE,
        singledf = TRUE,
        global = TRUE
      ),
```

```
      error = function(e) e
    )

    if (inherits(zph, "error")) {
      ph_full_list[[length(ph_full_list) + 1]] <- tibble(
        outcome = ol,
        n = nrow(des_ph$variables),
        events = sum(des_ph$variables[[ev]] == 1),
        term = NA_character_,
        chisq = NA_real_,
        df = NA_real_,
        p = NA_real_,
        status = "failed",
        error = conditionMessage(zph)
      )
    } else {
      ph_full_list[[length(ph_full_list) + 1]] <- extract_zph_table(
        zph,
        ol,
        nrow(des_ph$variables),
        sum(des_ph$variables[[ev]] == 1)
      )
    }
  }
}

ph_full_results <- bind_rows(ph_full_list) %>%
  mutate(p_formatted = format_p(p))

ph_summary <- map_dfr(main_outcomes$outcome_label, function(ol) {
  tmp <- ph_full_results %>% filter(outcome == ol, status == "ok")
  exposure_row <- tmp %>% filter(term == "exposure_quit")
  global_row <- tmp %>% filter(toupper(term) == "GLOBAL")
  exposure_p <- if (nrow(exposure_row) > 0) exposure_row$p[1] else NA_real_
  global_p <- if (nrow(global_row) > 0) global_row$p[1] else NA_real_
  tibble(
    outcome = ol,
    n = if (nrow(tmp) > 0) tmp$n[1] else NA_integer_,
    events = if (nrow(tmp) > 0) tmp$events[1] else NA_integer_,
    exposure_ph_p = exposure_p,
    global_ph_p = global_p,
    exposure_conclusion = case_when(
      !is.finite(exposure_p) ~ "Assessment unavailable",
      exposure_p < 0.05 ~ "Evidence of non-proportionality for smoking cessation",
      TRUE ~ "No statistical evidence of non-proportionality for smoking cessation"
    ),
    global_conclusion = case_when(
      !is.finite(global_p) ~ "Assessment unavailable",
      global_p < 0.05 ~ "Global model test indicates at least one time-varying
coefficient",
```

```
      TRUE ~ "No statistical evidence of global non-proportionality"
    )
  )
})

safe_write_csv(
  ph_full_results,
  file.path(DIRS$supp_tables, "Table_S8_full_proportional_hazards_tests.csv")
)
safe_write_csv(
  ph_summary,
  file.path(DIRS$results, "MODEL_proportional_hazards_summary.csv")
)

# ==============================================================================
# 15. 结果质量控制：异常窄置信区间、模型失败和命名
# ==============================================================================

cat("[11/14] 进行模型结果质量控制……\n")

all_model_results <- bind_rows(
  main_models_df,
  secondary_16_models,
  sensitivity_results_df,
  overlap_results,
  subgroup_results_df,
  quit_age_sensitivity_models
) %>%
  mutate(
    log_ci_width = ifelse(is.finite(CI_low) & is.finite(CI_high) & CI_low > 0,
                          log(CI_high) - log(CI_low), NA_real_),
    qc_suspiciously_narrow_ci = as.integer(
      is.finite(log_ci_width) & log_ci_width < 0.10 & events < 500
    ),
    qc_extreme_p = as.integer(is.finite(p) & p < 1e-20),
    HR_95CI = format_hr(HR, CI_low, CI_high)
  )

safe_write_csv(all_model_results, file.path(DIRS$results,
"03_all_model_results_with_QC_flags.csv"))

model_qc_summary <- all_model_results %>%
  summarise(
    total_rows = n(),
    successful_models = sum(status == "ok", na.rm = TRUE),
    failed_or_not_run = sum(status != "ok", na.rm = TRUE),
    suspiciously_narrow_CI = sum(qc_suspiciously_narrow_ci == 1, na.rm = TRUE),
    extreme_p_values = sum(qc_extreme_p == 1, na.rm = TRUE)
  )
safe_write_csv(model_qc_summary, file.path(DIRS$audit, "08_model_QC_summary.csv"))
```

```
# ==============================================================================
# 16. 生成TID投稿候选主表(最多5张)
# ==============================================================================

cat("[12/14] 生成5张主表和补充表……\n")

# ------------------------------------------------------------------------------
# Main Table 1:基线特征，连续变量和分类变量合并为一张表
# ------------------------------------------------------------------------------

continuous_labels <- c(
  age = "Age, years",
  survey_midyear = "NHANES survey midyear",
  years_since_dx = "Years from first cardiopulmonary diagnosis to NHANES",
  pre_dx_smoking_years = "Regular-smoking duration before diagnosis, years",
  pir = "Family poverty-to-income ratio",
  cig_per_day_proxy = "Cigarettes per day proxy",
  pack_years_proxy = "Pack-years proxy",
  bmi = "Body mass index, kg/m2"
)

main_table1_cont <- map_dfr(names(continuous_labels), function(v) {
  persist <- primary_df %>% filter(exposure_quit == 0)
  quit <- primary_df %>% filter(exposure_quit == 1)
  tibble(
    characteristic = continuous_labels[[v]],
    level = "",
    persistent_smokers = sprintf(
      "%.2f ± %.2f",
      weighted_mean_safe(persist[[v]], persist$wt_int_20y),
      weighted_sd_safe(persist[[v]], persist$wt_int_20y)
    ),
    postdiagnostic_quitters = sprintf(
      "%.2f ± %.2f",
      weighted_mean_safe(quit[[v]], quit$wt_int_20y),
      weighted_sd_safe(quit[[v]], quit$wt_int_20y)
    ),
    absolute_standardized_difference = abs(
      smd_continuous(primary_df[[v]], primary_df$exposure_quit,
primary_df$wt_int_20y)
    )
  )
})

categorical_labels <- c(
  sex_f = "Sex",
  race_f = "Race and ethnicity",
  education_f = "Education",
  disease_f = "Cardiopulmonary disease phenotype",
```

```
  diabetes_f = "Diabetes",
  hypertension_f = "Hypertension",
  age_group_f = "Age group"
)

main_table1_cat <- map_dfr(names(categorical_labels), function(v) {
  map_dfr(levels(droplevels(primary_df[[v]])), function(lv) {
    persist <- primary_df %>% filter(exposure_quit == 0)
    quit <- primary_df %>% filter(exposure_quit == 1)
    tibble(
      characteristic = categorical_labels[[v]],
      level = lv,
      persistent_smokers = sprintf(
        "%d (%.1f%%)",
        sum(persist[[v]] == lv, na.rm = TRUE),
        100 * weighted_prop_safe(persist[[v]], persist$wt_int_20y, lv)
      ),
      postdiagnostic_quitters = sprintf(
        "%d (%.1f%%)",
        sum(quit[[v]] == lv, na.rm = TRUE),
        100 * weighted_prop_safe(quit[[v]], quit$wt_int_20y, lv)
      ),
      absolute_standardized_difference = abs(
        smd_binary_level(primary_df[[v]], primary_df$exposure_quit,
primary_df$wt_int_20y, lv)
      )
    )
  })
})

main_table1 <- bind_rows(main_table1_cont, main_table1_cat) %>%
  mutate(
    absolute_standardized_difference = round(absolute_standardized_difference, 3)
  )

safe_write_csv(
  main_table1,
  file.path(DIRS$tables, "Table1_baseline_characteristics.csv")
)

# ------------------------------------------------------------------------------
# Main Table 2:主模型及1999-2014次要死因
# ------------------------------------------------------------------------------

main_table2 <- bind_rows(
  main_models_df %>%
    mutate(
      analysis_window = "NHANES 1999-2018",
      model = case_when(
        grepl("M0_unadjusted$", analysis) ~ "Unadjusted",
```

```
        grepl("M1_demographic$", analysis) ~ "Demographic-adjusted",
        grepl("M2_core$", analysis) ~ "Fully adjusted core model",
        TRUE ~ analysis
      )
    ),
  secondary_16_models %>%
    mutate(
      analysis_window = "NHANES 1999–2014",
      model = "Fully adjusted core model"
    )
) %>%
  select(
    analysis_window,
    outcome,
    model,
    n,
    events,
    n_quit,
    n_persistent,
    HR,
    CI_low,
    CI_high,
    p,
    HR_95CI,
    p_formatted,
    status
  )

safe_write_csv(
  main_table2,
  file.path(DIRS$tables, "Table2_primary_and_secondary_mortality_models.csv")
)

# ---------------------------------------------------------------------------
# Main Table 3：landmark、常规敏感性、重叠加权和戒烟年龄敏感性
# ---------------------------------------------------------------------------

main_table3 <- bind_rows(
  sensitivity_results_df %>%
    mutate(analysis_family = "Conventional and landmark sensitivity"),
  overlap_results %>%
    mutate(analysis_family = "Overlap-weighted supportive analysis"),
  quit_age_sensitivity_models %>%
    mutate(analysis_family = "Quit-age reconstruction sensitivity")
) %>%
  filter(status == "ok") %>%
  select(
    analysis_family,
    analysis,
    outcome,
```

```r
    n,
    events,
    n_quit,
    n_persistent,
    HR,
    CI_low,
    CI_high,
    p,
    HR_95CI,
    p_formatted
  )

safe_write_csv(
  main_table3,
  file.path(DIRS$tables, "Table3_sensitivity_and_landmark_analyses.csv")
)

# ------------------------------------------------------------------------------
# Main Table 4:亚组估计与交互作用
# ------------------------------------------------------------------------------

main_table4 <- subgroup_results_df %>%
  left_join(
    interaction_results_df %>%
      select(subgroup_variable, interaction_p, interaction_p_formatted,
status_interaction = status, error_interaction = error),
    by = "subgroup_variable"
  ) %>%
  select(
    subgroup_label,
    subgroup_level,
    n,
    events,
    n_quit,
    n_persistent,
    HR,
    CI_low,
    CI_high,
    p,
    HR_95CI,
    p_formatted,
    interaction_p,
    interaction_p_formatted,
    status,
    error,
    status_interaction,
    error_interaction
  )

safe_write_csv(
```

```
  main_table4,
  file.path(DIRS$tables, "Table4_subgroup_and_interaction_analyses.csv")
)

# --------------------------------------------------------------------------------
# Main Table 5:比例风险假设
# --------------------------------------------------------------------------------

main_table5 <- ph_summary %>%
  mutate(
    exposure_ph_p_formatted = format_p(exposure_ph_p),
    global_ph_p_formatted = format_p(global_ph_p)
  )

safe_write_csv(
  main_table5,
  file.path(DIRS$tables, "Table5_proportional_hazards_assessment.csv")
)

# 其余内部审计和完整模型结果作为补充表，不计入5张主表。
safe_write_csv(
  missingness,
  file.path(DIRS$supp_tables, "Table_S9_missingness_primary_dataset.csv")
)
safe_write_csv(
  balance_results,
  file.path(DIRS$supp_tables, "Table_S10_overlap_weighting_covariate_balance.csv")
)
safe_write_csv(
  all_model_results,
  file.path(DIRS$supp_tables, "Table_S11_all_model_results_with_QC_flags.csv")
)
safe_write_csv(
  interaction_results_df,
  file.path(DIRS$supp_tables, "Table_S12_interaction_test_details.csv")
)

# ================================================================================
# 17. 生成4幅复合主图（不生成补充图）
# ================================================================================

cat("[13/14] 生成4幅复合主图和源数据……\n")

PERSIST_COLOR <- "#6B7280"
QUIT_COLOR <- "#C56A11"
MODEL_COLORS <- c(
  "Unadjusted" = "#9CA3AF",
  "Demographic-adjusted" = "#6B7280",
  "Fully adjusted core model" = "#C56A11"
)
```

```r
# ------------------------------------------------------------------------------
# Figure 1:A 队列流程;B 各周期暴露组构成
# ------------------------------------------------------------------------------

fig1a_data <- cohort_flow %>%
  mutate(step = factor(step, levels = rev(step)))

fig1b_data <- analytic_cycle_audit %>%
  select(cycle, begin_year, n_postdiagnostic_quitters, n_persistent_smokers) %>%
  pivot_longer(
    cols = c(n_postdiagnostic_quitters, n_persistent_smokers),
    names_to = "exposure_group",
    values_to = "n"
  ) %>%
  mutate(
    exposure_group = dplyr::recode(
      exposure_group,
      n_postdiagnostic_quitters = "Post-diagnostic quitter",
      n_persistent_smokers = "Persistent smoker"
    ),
    cycle = factor(cycle, levels = analytic_cycle_audit$cycle)
  )

safe_write_csv(fig1a_data, file.path(DIRS$source_data, "Figure1A_cohort_flow.csv"))
safe_write_csv(fig1b_data, file.path(DIRS$source_data,
"Figure1B_cycle_composition.csv"))

p1a <- ggplot(fig1a_data, aes(x = n, y = step)) +
  geom_col(width = 0.65, fill = QUIT_COLOR) +
  geom_text(aes(label = scales::comma(n)), hjust = -0.08, size = 3.0, family =
BASE_FAMILY) +
  scale_x_continuous(labels = scales::comma, expand = expansion(mult = c(0, 0.20)))
+
  labs(x = "Unweighted participants", y = NULL) +
  pub_theme(9) +
  theme(legend.position = "none")

p1b <- ggplot(fig1b_data, aes(x = cycle, y = n, fill = exposure_group)) +
  geom_col(width = 0.72) +
  scale_fill_manual(values = c(
    "Persistent smoker" = PERSIST_COLOR,
    "Post-diagnostic quitter" = QUIT_COLOR
  )) +
  scale_y_continuous(labels = scales::comma) +
  labs(x = "NHANES cycle", y = "Participants", fill = NULL) +
  pub_theme(9) +
  theme(axis.text.x = element_text(angle = 45, hjust = 1))

figure1 <- p1a + p1b +
```

```
  patchwork::plot_layout(widths = c(1.35, 1)) +
  patchwork::plot_annotation(tag_levels = "A")

save_pub_figure(figure1, "Figure1_cohort_and_cycle_composition", 13.0, 6.8)

# ---------------------------------------------------------------------------
# Figure 2：A 三层调整主模型；B 完全调整主要与次要结局
# ---------------------------------------------------------------------------

fig2a_data <- main_models_df %>%
  filter(status == "ok") %>%
  mutate(
    model = case_when(
      grepl("M0_unadjusted$", analysis) ~ "Unadjusted",
      grepl("M1_demographic$", analysis) ~ "Demographic-adjusted",
      grepl("M2_core$", analysis) ~ "Fully adjusted core model",
      TRUE ~ analysis
    ),
    model = factor(model, levels = c("Unadjusted", "Demographic-adjusted", "Fully
adjusted core model")),
    outcome = factor(outcome, levels = c("Cancer mortality", "Heart-disease
mortality", "All-cause mortality"))
  )

fig2b_data <- bind_rows(
  main_models_df %>%
    filter(grepl("M2_core$", analysis), status == "ok"),
  secondary_16_models %>%
    filter(status == "ok")
) %>%
  mutate(outcome_plot = factor(outcome, levels = rev(unique(outcome))))

safe_write_csv(fig2a_data, file.path(DIRS$source_data,
"Figure2A_primary_models.csv"))
safe_write_csv(fig2b_data, file.path(DIRS$source_data,
"Figure2B_fully_adjusted_outcomes.csv"))

p2a <- ggplot(fig2a_data, aes(x = HR, y = model, color = model)) +
  geom_vline(xintercept = 1, linetype = "dashed", linewidth = 0.45) +
  geom_segment(aes(x = CI_low, xend = CI_high, yend = model), linewidth = 0.75) +
  geom_point(size = 2.4) +
  facet_wrap(~outcome, ncol = 1) +
  scale_x_log10() +
  scale_color_manual(values = MODEL_COLORS) +
  labs(x = "Hazard ratio (log scale)", y = NULL, color = NULL) +
  pub_theme(9) +
  theme(legend.position = "none")

p2b <- ggplot(fig2b_data, aes(x = HR, y = outcome_plot)) +
  geom_vline(xintercept = 1, linetype = "dashed", linewidth = 0.45) +
```

```
  geom_segment(aes(x = CI_low, xend = CI_high, yend = outcome_plot), linewidth =
0.75, color = QUIT_COLOR) +
  geom_point(size = 2.5, color = QUIT_COLOR) +
  scale_x_log10() +
  labs(x = "Fully adjusted hazard ratio (log scale)", y = NULL) +
  pub_theme(9) +
  theme(legend.position = "none")

figure2 <- p2a + p2b +
  patchwork::plot_layout(widths = c(1.15, 1)) +
  patchwork::plot_annotation(tag_levels = "A")

save_pub_figure(figure2, "Figure2_primary_and_secondary_mortality_models", 12.5,
8.4)

# ------------------------------------------------------------------------------
# Figure 3:A landmark;B 心脏疾病死亡的完整敏感性分析
# ------------------------------------------------------------------------------

fig3a_data <- bind_rows(
  main_models_df %>%
    filter(
      grepl("M2_core$", analysis),
      outcome %in% c("All-cause mortality", "Heart-disease mortality")
    ) %>%
    transmute(
      analysis = "Baseline",
      landmark_months = 0,
      outcome,
      HR, CI_low, CI_high, p, n, events, status
    ),
  landmark_results_df %>%
    transmute(
      analysis = paste0(landmark_months, "-month landmark"),
      landmark_months,
      outcome,
      HR, CI_low, CI_high, p, n, events, status
    )
) %>%
  filter(status == "ok") %>%
  mutate(
    analysis = factor(
      analysis,
      levels = c("Baseline", "12-month landmark", "24-month landmark", "36-month
landmark")
    )
  )

fig3b_data <- bind_rows(
  main_models_df %>%
```

```
    filter(grepl("M2_core$", analysis), outcome == "Heart-disease mortality") %>%
    mutate(analysis_display = "Primary fully adjusted model"),
  sensitivity_results_df %>%
    filter(outcome == "Heart-disease mortality") %>%
    mutate(analysis_display = analysis),
  overlap_results %>%
    filter(outcome == "Heart-disease mortality") %>%
    mutate(analysis_display = analysis),
  quit_age_sensitivity_models %>%
    filter(outcome == "Heart-disease mortality") %>%
    mutate(analysis_display = analysis)
) %>%
  filter(status == "ok", !grepl("Landmark", analysis_display)) %>%
  distinct(analysis_display, .keep_all = TRUE) %>%
  mutate(
    analysis_display = factor(analysis_display, levels =
rev(unique(analysis_display)))
  )

safe_write_csv(fig3a_data, file.path(DIRS$source_data,
"Figure3A_landmark_analyses.csv"))
safe_write_csv(fig3b_data, file.path(DIRS$source_data,
"Figure3B_heart_mortality_sensitivity.csv"))

p3a <- ggplot(fig3a_data, aes(x = HR, y = analysis)) +
  geom_vline(xintercept = 1, linetype = "dashed", linewidth = 0.45) +
  geom_segment(aes(x = CI_low, xend = CI_high, yend = analysis), linewidth = 0.72,
color = QUIT_COLOR) +
  geom_point(size = 2.3, color = QUIT_COLOR) +
  facet_wrap(~outcome, ncol = 1) +
  scale_x_log10() +
  labs(x = "Hazard ratio (log scale)", y = NULL) +
  pub_theme(9) +
  theme(legend.position = "none")

p3b <- ggplot(fig3b_data, aes(x = HR, y = analysis_display)) +
  geom_vline(xintercept = 1, linetype = "dashed", linewidth = 0.45) +
  geom_segment(aes(x = CI_low, xend = CI_high, yend = analysis_display), linewidth =
0.70, color = QUIT_COLOR) +
  geom_point(size = 2.2, color = QUIT_COLOR) +
  scale_x_log10() +
  labs(x = "Hazard ratio for heart-disease mortality (log scale)", y = NULL) +
  pub_theme(8.5) +
  theme(legend.position = "none")

figure3 <- p3a + p3b +
  patchwork::plot_layout(widths = c(0.95, 1.45)) +
  patchwork::plot_annotation(tag_levels = "A")

save_pub_figure(figure3, "Figure3_landmark_and_sensitivity_analyses", 14.0, 9.0)
```

```
# ---------------------------------------------------------------------------
# Figure 4:A 重叠加权平衡;B 预设亚组
# ---------------------------------------------------------------------------

if ("label" %in% names(balance_results) && nrow(balance_results) > 0) {
  fig4a_data <- balance_results %>%
    filter(is.finite(abs_smd_survey) | is.finite(abs_smd_overlap)) %>%
    select(label, abs_smd_survey, abs_smd_overlap) %>%
    pivot_longer(
      cols = c(abs_smd_survey, abs_smd_overlap),
      names_to = "weighting",
      values_to = "absolute_smd"
    ) %>%
    mutate(
      weighting = dplyr::recode(
        weighting,
        abs_smd_survey = "Survey weight only",
        abs_smd_overlap = "Survey × overlap weight"
      )
    ) %>%
    group_by(label) %>%
    mutate(max_smd = max(absolute_smd, na.rm = TRUE)) %>%
    ungroup() %>%
    arrange(max_smd) %>%
    mutate(label = factor(label, levels = unique(label)))
} else {
  fig4a_data <- tibble(
    label = factor("Balance unavailable"),
    weighting = "Survey weight only",
    absolute_smd = NA_real_
  )
}

fig4b_data <- subgroup_results_df %>%
  filter(status == "ok", is.finite(HR), is.finite(CI_low), is.finite(CI_high)) %>%
  mutate(
    subgroup_display = paste0(subgroup_label, ": ", subgroup_level),
    subgroup_display = factor(subgroup_display, levels =
rev(unique(subgroup_display)))
  )

safe_write_csv(fig4a_data, file.path(DIRS$source_data,
"Figure4A_overlap_balance.csv"))
safe_write_csv(fig4b_data, file.path(DIRS$source_data,
"Figure4B_subgroup_estimates.csv"))

p4a <- ggplot(fig4a_data, aes(x = absolute_smd, y = label, shape = weighting)) +
  geom_vline(xintercept = 0.10, linetype = "dashed", linewidth = 0.45) +
  geom_point(size = 2.0) +
```

```r
  labs(x = "Absolute standardized mean difference", y = NULL, shape = NULL) +
  pub_theme(8.5)

p4b <- ggplot(fig4b_data, aes(x = HR, y = subgroup_display)) +
  geom_vline(xintercept = 1, linetype = "dashed", linewidth = 0.45) +
  geom_segment(aes(x = CI_low, xend = CI_high, yend = subgroup_display), linewidth =
0.70, color = QUIT_COLOR) +
  geom_point(size = 2.2, color = QUIT_COLOR) +
  scale_x_log10() +
  labs(x = "Hazard ratio for heart-disease mortality (log scale)", y = NULL) +
  pub_theme(8.5) +
  theme(legend.position = "none")

figure4 <- p4a + p4b +
  patchwork::plot_layout(widths = c(1.15, 1)) +
  patchwork::plot_annotation(tag_levels = "A")

save_pub_figure(figure4, "Figure4_overlap_balance_and_subgroups", 13.5, 8.0)

figure_captions <- c(
  "Figure 1. Construction and composition of the diagnosis-anchored linked mortality
cohort. Panel A shows sequential cohort selection. Panel B shows the unweighted
distribution of persistent smokers and post-diagnostic quitters across NHANES
cycles.",
  "Figure 2. Associations between post-diagnostic smoking cessation and mortality.
Panel A presents unadjusted, demographic-adjusted, and fully adjusted
survey-weighted Cox estimates for the three 1999–2018 outcomes. Panel B presents
fully adjusted estimates for the main outcomes and the two cause-specific outcomes
restricted to 1999–2014. Persistent smoking is the reference category.",
  "Figure 3. Landmark and sensitivity analyses. Panel A presents baseline and 12-,
24-, and 36-month landmark analyses with the time origin reset at each landmark.
Panel B summarizes sensitivity analyses for heart-disease mortality, including
calendar-cycle, period-restriction, exposure-definition, MEC-weighted,
smoking-burden, disease-restriction, overlap-weighted, and quit-age reconstruction
analyses.",
  "Figure 4. Supportive weighting and subgroup analyses. Panel A shows absolute
standardized mean differences before and after combining NHANES interview weights
with overlap weights. Panel B shows prespecified subgroup estimates for
heart-disease mortality. Subgroup estimates are exploratory."
)
writeLines(
  figure_captions,
  file.path(DIRS$reports, "FIGURE_CAPTIONS.txt"),
  useBytes = TRUE
)

# ================================================================================
# 18. 运行总结、投稿结构核查和sessionInfo
# ================================================================================
```

```
cat("[14/14] 输出运行总结(不生成压缩包)……\n")

main_m2_summary <- main_models_df %>%
  filter(grepl("M2_core$", analysis)) %>%
  select(outcome, n, events, HR, CI_low, CI_high, p, HR_95CI, status, error)
safe_write_csv(
  main_m2_summary,
  file.path(DIRS$results, "FINAL_main_M2_results_for_manuscript_update.csv")
)

manuscript_update_text <- c(
  "REQUIRED MANUSCRIPT UPDATES AFTER THE FINAL INTEGRATED REANALYSIS",
  "",
  "1. Replace all numerical results from the old manuscript with this run.",
  "2. Describe the study as a diagnosis-anchored observational linked mortality
cohort analysis, not a target-trial emulation.",
  "3. Use heart-disease mortality for UCOD_LEADING=001; do not call it broad
cardiovascular mortality.",
  "4. Report cardiovascular composite mortality (001+005) and chronic lower
respiratory disease mortality (003) only for NHANES 1999–2014.",
  "5. State that the primary analysis used combined interview weights and
interview-date follow-up; the BMI-expanded analysis used MEC weights and
examination-date follow-up.",
  "6. State the combined-weight formula: 1999–2002 four-year weight ×1/5 and each
2003–2018 two-year weight ÷10.",
  "7. Describe formal models as survey-weighted Cox proportional hazards models
fitted with survey::svycoxph.",
  "8. State that MCD180* aliases were used for 2017–2018 diagnosis ages and quit age
was derived from SMQ050Q/U when direct SMD055 was unavailable; the >=50-year
category was not treated as an exact value.",
  "9. Report the quit-age discrepancy exclusion and direct-quit-age-only analyses as
sensitivity analyses.",
  "10. Report the exposure-specific and global cox.zph tests. Do not claim that
P>=0.05 proves proportional hazards.",
  "11. Landmark analyses require follow-up beyond each landmark and reset time zero
to the landmark.",
  "12. Overlap-weighted models are supportive and do not replace the primary
complex-survey regression.",
  "13. The submission candidate contains exactly five main tables and four main
figures; no supplementary figures are generated.",
  "14. All remaining detailed audits and full model outputs are supplementary tables
or internal QC files."
)
writeLines(
  manuscript_update_text,
  file.path(DIRS$reports, "MANUSCRIPT_UPDATE_REQUIRED.txt"),
  useBytes = TRUE
)

main_table_files <- list.files(DIRS$tables, pattern = "^Table[1-5]_.*\\.csv$",
```

```
full.names = TRUE)
main_figure_tiff_files <- list.files(DIRS$figures, pattern =
"^Figure[1-4]_.*\\.tiff$", full.names = TRUE)
main_figure_pdf_files <- list.files(DIRS$figures, pattern =
"^Figure[1-4]_.*\\.pdf$", full.names = TRUE)
supp_figure_files <- list.files(DIRS$figures, pattern = "FigureS|supp", ignore.case
= TRUE, full.names = TRUE)

submission_structure_audit <- tibble(
  item = c(
    "Main table CSV files",
    "Main figure TIFF files",
    "Main figure PDF files",
    "Supplementary figure files",
    "TID table limit respected",
    "TID figure limit respected"
  ),
  value = c(
    length(main_table_files),
    length(main_figure_tiff_files),
    length(main_figure_pdf_files),
    length(supp_figure_files),
    length(main_table_files) <= 5,
    length(main_figure_tiff_files) <= 4
  )
)
safe_write_csv(
  submission_structure_audit,
  file.path(DIRS$reports, "TID_SUBMISSION_STRUCTURE_AUDIT.csv")
)

if (length(main_table_files) != 5) {
  warning("最终主表数量不是5张:", length(main_table_files))
}
if (WRITE_TIFF && length(main_figure_tiff_files) != 4) {
  warning("最终TIFF主图数量不是4幅:", length(main_figure_tiff_files))
}
if (length(supp_figure_files) > 0) {
  stop("检测到补充图文件;本脚本设计为不生成补充图。")
}

capture.output(
  sessionInfo(),
  file = file.path(DIRS$logs, paste0("sessionInfo_", run_stamp, ".txt"))
)

final_summary <- tibble(
  item = c(
    "Run completed",
    "Data access mode",
```

```r
    "Project root",
    "Primary sample size",
    "Post-diagnostic quitters",
    "Persistent smokers",
    "Primary heart-disease deaths",
    "Primary all-cause deaths",
    "Successful model rows",
    "Failed/not-run model rows",
    "Suspiciously narrow CI flags",
    "Main tables",
    "Main figures",
    "Supplementary figures",
    "Raw-data directory modified"
  ),
  value = c(
    format(Sys.time()),
    "Local 00_raw_data only; no download",
    PROJECT_ROOT,
    nrow(primary_df),
    sum(primary_df$exposure_quit == 1),
    sum(primary_df$exposure_quit == 0),
    sum(primary_df$event_heart == 1),
    sum(primary_df$event_allcause == 1),
    model_qc_summary$successful_models,
    model_qc_summary$failed_or_not_run,
    model_qc_summary$suspiciously_narrow_CI,
    length(main_table_files),
    length(main_figure_tiff_files),
    length(supp_figure_files),
    "No; read only"
  )
)
safe_write_csv(
  final_summary,
  file.path(DIRS$reports, "FINAL_RUN_SUMMARY.csv")
)

current_source <- tryCatch(
  normalizePath(sys.frame(1)$ofile, winslash = "/", mustWork = FALSE),
  error = function(e) NA_character_
)
if (
  is.character(current_source) &&
  length(current_source) == 1 &&
  !is.na(current_source) &&
  file.exists(current_source)
) {
  file.copy(
    current_source,
    file.path(DIRS$code, "TID_MAJOR_REVISION_FINAL_ALL_IN_ONE_COMPLETE_RUN_v1.R"),
```

```
    overwrite = TRUE
  )
}

# 按用户要求：本最终脚本不生成ZIP压缩包。
zip_generation_status <- "DISABLED"

cat("\n============================================================\n")
cat("最终整合重新分析完成：", format(Sys.time()), "\n", sep = "")
cat("主分析样本量：", nrow(primary_df), "\n", sep = "")
cat("诊断后戒烟者：", sum(primary_df$exposure_quit == 1), "\n", sep = "")
cat("持续吸烟者：", sum(primary_df$exposure_quit == 0), "\n", sep = "")
cat("心脏疾病死亡事件：", sum(primary_df$event_heart == 1), "\n", sep = "")
cat("主表数量：", length(main_table_files), "\n", sep = "")
cat("主图数量：", length(main_figure_tiff_files), "\n", sep = "")
cat("补充图数量：", length(supp_figure_files), "\n", sep = "")
cat("ZIP压缩包：未生成（按用户要求）\n")
cat("全部结果保存在项目输出目录中。\n")
cat("============================================================\n")

sink()
################################################################################
# 18. TID-02054-2026-01 MAJOR REVISION 专用模块
#
# V2关键修正：
# - 不再附加car包，避免同名函数屏蔽dplyr::recode；
# - 所有recode调用均显式写为dplyr::recode；
# - 保留V1已由R 4.6.0实际运行验证通过的完整数据重建、模型、
#   VIF/GVIF、1999–2014扩展模型、加权生存曲线和表格生成部分。
#
# 本模块在上方“完整原始数据重建与既有分析链”成功运行后执行。
# 新增内容：
# 1) 核心样本和既有主要结果的硬性复现核验；
# 2) 完全调整核心模型协变量的VIF/GVIF诊断；
# 3) 1999–2014限定时期两个结局的M0、M1、M2模型；
# 4) 心脏病死亡的NHANES调查加权生存曲线；
# 5) Figure 1标准流程图及Figure 2返修复合图；
# 6) Table 1和Table 2返修版CSV/XLSX；
# 7) 补充表重新编号建议及复制；
# 8) 可直接用于逐点回复信的动态结果摘要；
# 9) 返修结果一致性审计；不生成压缩包。
################################################################################

while (sink.number() > 0) sink()

REVISION_STAMP <- format(Sys.time(), "%Y%m%d_%H%M%S")
REVISION_ROOT <- file.path(PROJECT_ROOT, "10_TID_MAJOR_REVISION_FINAL")

REV_DIRS <- list(
  root = REVISION_ROOT,
```

```
  code = file.path(REVISION_ROOT, "01_code"),
  audit = file.path(REVISION_ROOT, "02_audit"),
  results = file.path(REVISION_ROOT, "03_results"),
  tables = file.path(REVISION_ROOT, "04_tables"),
  figures = file.path(REVISION_ROOT, "05_figures"),
  source_data = file.path(REVISION_ROOT, "05_figures", "source_data"),
  supplementary = file.path(REVISION_ROOT, "06_supplementary_tables_RENUMBERED"),
  response = file.path(REVISION_ROOT, "07_response_ready"),
  logs = file.path(REVISION_ROOT, "08_logs")
)
invisible(lapply(REV_DIRS, fs::dir_create))

revision_log_file <- file.path(
  REV_DIRS$logs,
  paste0("TID_major_revision_log_", REVISION_STAMP, ".txt")
)
sink(revision_log_file, split = TRUE)

cat("\n================================================================\n")
cat("TID-02054-2026-01 MAJOR REVISION MODULE\n")
cat("Started: ", format(Sys.time()), "\n", sep = "")
cat("Locked source project: ", SOURCE_PROJECT_ROOT, "\n", sep = "")
cat("Revision output root: ", REVISION_ROOT, "\n", sep = "")
cat("Research-data download: DISABLED\n")
cat("================================================================\n\n")

# ---------------------------------------------------------------------------
# 18.1 返修模块附加R包
# ---------------------------------------------------------------------------

revision_required_packages <- c("car", "openxlsx")

installed_revision <- rownames(installed.packages())
if (AUTO_INSTALL_PACKAGES) {
  for (pkg in revision_required_packages) {
    if (!pkg %in% installed_revision) {
      try(
        install.packages(
          pkg,
          repos = "https://cloud.r-project.org",
          dependencies = TRUE
        ),
        silent = TRUE
      )
    }
  }
}

missing_revision_packages <- revision_required_packages[
  !vapply(
```

```r
    revision_required_packages,
    requireNamespace,
    logical(1),
    quietly = TRUE
  )
]

if (length(missing_revision_packages) > 0) {
  stop(
    "返修模块缺少必需R包:",
    paste(missing_revision_packages, collapse = ", "),
    "\n请安装后重新运行。"
  )
}

# 不把car或openxlsx附加到R搜索路径。
# 原因:car包中的同名函数会屏蔽dplyr::recode;本脚本统一使用显式命名空间,
# 从根本上避免函数冲突。
invisible(NULL)

# ------------------------------------------------------------------------------
# 18.2 通用返修函数
# ------------------------------------------------------------------------------

write_lines_utf8 <- function(x, path) {
  con <- file(path, open = "wt", encoding = "UTF-8")
  on.exit(close(con), add = TRUE)
  writeLines(x, con = con, useBytes = TRUE)
}

copy_if_exists <- function(from, to) {
  if (file.exists(from)) {
    fs::dir_create(dirname(to))
    return(file.copy(from, to, overwrite = TRUE))
  }
  warning("拟复制文件不存在:", from)
  FALSE
}

model_label_from_analysis <- function(x) {
  dplyr::case_when(
    grepl("M0_unadjusted$", x) ~ "M0: Unadjusted",
    grepl("M1_demographic$", x) ~ "M1: Demographic-adjusted",
    grepl("M2_core$", x) ~ "M2: Fully adjusted core model",
    TRUE ~ x
  )
}

extract_svykm_curve <- function(curve_object, group_name) {
  if (is.null(curve_object$time) || is.null(curve_object$surv)) {
```

```
    stop("svykm对象缺少time或surv成员，无法提取源数据。")
  }

  tt <- as.numeric(curve_object$time)
  ss <- as.numeric(curve_object$surv)
  n_keep <- min(length(tt), length(ss))
  tt <- tt[seq_len(n_keep)]
  ss <- ss[seq_len(n_keep)]

  tibble(
    exposure_group = group_name,
    time_years = c(0, tt),
    survival_probability = c(1, ss)
  ) %>%
    distinct(exposure_group, time_years, .keep_all = TRUE) %>%
    arrange(time_years)
}

make_column_vif <- function(model_matrix) {
  mm <- as.matrix(model_matrix)
  if ("(Intercept)" %in% colnames(mm)) {
    mm <- mm[, colnames(mm) != "(Intercept)", drop = FALSE]
  }

  finite_cols <- vapply(
    seq_len(ncol(mm)),
    function(j) {
      x <- mm[, j]
      all(is.finite(x)) && stats::sd(x) > 0
    },
    logical(1)
  )
  mm <- mm[, finite_cols, drop = FALSE]

  if (ncol(mm) < 2) {
    return(tibble(
      model_matrix_column = colnames(mm),
      VIF = NA_real_,
      tolerance = NA_real_
    ))
  }

  out <- lapply(seq_len(ncol(mm)), function(j) {
    y <- mm[, j]
    x <- mm[, -j, drop = FALSE]

    fit <- tryCatch(
      stats::lm.fit(
        x = cbind("(Intercept)" = 1, x),
        y = y
```

```
      ),
      error = function(e) e
    )

    if (inherits(fit, "error")) {
      return(tibble(
        model_matrix_column = colnames(mm)[j],
        VIF = NA_real_,
        tolerance = NA_real_
      ))
    }

    rss <- sum(fit$residuals^2)
    tss <- sum((y - mean(y))^2)
    r2 <- ifelse(tss > 0, 1 - rss/tss, NA_real_)
    r2 <- min(max(r2, 0), 0.999999999)
    vif_value <- 1/(1-r2)

    tibble(
      model_matrix_column = colnames(mm)[j],
      VIF = vif_value,
      tolerance = 1/vif_value
    )
  })

  bind_rows(out) %>%
    arrange(desc(VIF))
}

save_revision_figure <- function(plot_object, stem, width, height) {
  if (WRITE_TIFF) {
    ggplot2::ggsave(
      filename = file.path(REV_DIRS$figures, paste0(stem, ".tiff")),
      plot = plot_object,
      width = width,
      height = height,
      units = "in",
      dpi = DPI,
      device = "tiff",
      compression = "lzw",
      bg = "white",
      limitsize = FALSE
    )
  }

  if (WRITE_PDF) {
    ggplot2::ggsave(
      filename = file.path(REV_DIRS$figures, paste0(stem, ".pdf")),
      plot = plot_object,
      width = width,
```

```
      height = height,
      units = "in",
      device = grDevices::cairo_pdf,
      bg = "white",
      limitsize = FALSE
    )
  }
}

# ------------------------------------------------------------------------------
# 18.3 核心数据和主要结果硬性复现核验
# ------------------------------------------------------------------------------

cat("[REV 1/10] 核验核心样本、事件数和主要效应估计……\n")

heart_m2_check <- main_models_df %>%
  filter(
    outcome == "Heart-disease mortality",
    grepl("M2_core$", analysis),
    status == "ok"
  ) %>%
  slice(1)

if (nrow(heart_m2_check) != 1) {
  stop("无法唯一识别1999-2018心脏病死亡M2核心模型。")
}

expected_check <- tibble(
  metric = c(
    "Primary analysis n",
    "Post-diagnostic quitters",
    "Persistent smokers",
    "All-cause deaths",
    "Heart-disease deaths",
    "Cancer deaths",
    "Heart-disease M2 HR",
    "Heart-disease M2 CI lower",
    "Heart-disease M2 CI upper"
  ),
  expected = c(
    2319,
    975,
    1344,
    999,
    272,
    244,
    0.586,
    0.422,
    0.815
  ),
```

```
  actual = c(
    nrow(primary_df),
    sum(primary_df$exposure_quit == 1),
    sum(primary_df$exposure_quit == 0),
    sum(primary_df$event_allcause == 1),
    sum(primary_df$event_heart == 1),
    sum(primary_df$event_cancer == 1),
    heart_m2_check$HR,
    heart_m2_check$CI_low,
    heart_m2_check$CI_high
  ),
  tolerance = c(
    0, 0, 0, 0, 0, 0,
    0.010, 0.015, 0.015
  )
) %>%
  mutate(
    absolute_difference = abs(actual - expected),
    pass = absolute_difference <= tolerance
  )

safe_write_csv(
  expected_check,
  file.path(REV_DIRS$audit, "REVISION_CORE_REPRODUCIBILITY_CHECK.csv")
)

if (!all(expected_check$pass)) {
  failed_metrics <- expected_check %>%
    filter(!pass) %>%
    pull(metric)
  stop(
    "核心结果未通过复现核验:",
    paste(failed_metrics, collapse = "; "),
    "。停止返修新增分析。"
  )
}

# 与上次锁定结果文件逐行比较(如旧结果文件仍存在)。
old_main_model_path <- file.path(
  SOURCE_PROJECT_ROOT,
  "04_results",
  "MODEL_primary_survey_weighted_cox_models.csv"
)

if (file.exists(old_main_model_path)) {
  old_main_models <- readr::read_csv(
    old_main_model_path,
    show_col_types = FALSE,
    progress = FALSE
  )
```

```
  revision_vs_old <- main_models_df %>%
    select(outcome, analysis, HR_new = HR, CI_low_new = CI_low,
           CI_high_new = CI_high, p_new = p) %>%
    left_join(
      old_main_models %>%
        select(outcome, analysis, HR_old = HR, CI_low_old = CI_low,
               CI_high_old = CI_high, p_old = p),
      by = c("outcome", "analysis")
    ) %>%
    mutate(
      abs_diff_HR = abs(HR_new - HR_old),
      abs_diff_CI_low = abs(CI_low_new - CI_low_old),
      abs_diff_CI_high = abs(CI_high_new - CI_high_old),
      abs_diff_p = abs(p_new - p_old),
      exact_within_1e_8 =
        abs_diff_HR < 1e-8 &
        abs_diff_CI_low < 1e-8 &
        abs_diff_CI_high < 1e-8 &
        abs_diff_p < 1e-8
    )

  safe_write_csv(
    revision_vs_old,
    file.path(REV_DIRS$audit, "REVISION_VS_LOCKED_PRIMARY_MODELS.csv")
  )
}

# ------------------------------------------------------------------------------
# 18.4 VIF/GVIF多重共线性诊断
# ------------------------------------------------------------------------------

cat("[REV 2/10] 计算VIF/GVIF多重共线性诊断……\n")

VIF_THRESHOLD <- 5

vif_variables <- c(
  "time_int_years",
  "exposure_quit",
  "age10",
  "sex_f",
  "race_f",
  "education_f",
  "disease_f",
  "years_since_dx5",
  "pre_dx_smoking_years10",
  "survey_cycle_2y_centered"
)

vif_data <- primary_df %>%
```

```
  select(all_of(vif_variables)) %>%
  filter(stats::complete.cases(.)) %>%
  droplevels()

vif_lm_formula <- stats::as.formula(
  paste0(
    "time_int_years ~ ",
    RHS_MODELS$M2_core
  )
)

# VIF只用于同一协变量设计矩阵的共线性诊断；
# 主要效应推断仍来自survey::svycoxph。
vif_lm_fit <- stats::lm(
  formula = vif_lm_formula,
  data = vif_data,
  na.action = na.fail
)

vif_raw <- car::vif(vif_lm_fit)

if (is.matrix(vif_raw)) {
  vif_term_table <- as.data.frame(vif_raw) %>%
    tibble::rownames_to_column("term")

  adjusted_col <- grep(
    "GVIF\\^",
    names(vif_term_table),
    value = TRUE
  )

  if (length(adjusted_col) == 0) {
    adjusted_col <- grep(
      "GVIF",
      names(vif_term_table),
      value = TRUE
    )
    adjusted_col <- adjusted_col[length(adjusted_col)]
  }

  vif_term_table <- vif_term_table %>%
    mutate(
      adjusted_GVIF = .data[[adjusted_col[1]]],
      threshold = VIF_THRESHOLD,
      meaningful_collinearity = adjusted_GVIF >= threshold
    )
} else {
  vif_term_table <- tibble(
    term = names(vif_raw),
    VIF = as.numeric(vif_raw),
```

```
    adjusted_GVIF = as.numeric(vif_raw),
    threshold = VIF_THRESHOLD,
    meaningful_collinearity = adjusted_GVIF >= threshold
  )
}

vif_model_matrix <- stats::model.matrix(
  stats::as.formula(paste0("~", RHS_MODELS$M2_core)),
  data = vif_data
)

vif_column_table <- make_column_vif(vif_model_matrix) %>%
  mutate(
    threshold = VIF_THRESHOLD,
    meaningful_collinearity = VIF >= threshold
  )

vif_summary <- tibble(
  diagnostic = c(
    "Complete cases used",
    "Maximum adjusted term-level GVIF",
    "Maximum model-matrix column VIF",
    "Prespecified threshold",
    "Any term-level value at or above threshold",
    "Any column-level value at or above threshold"
  ),
  value = c(
    nrow(vif_data),
    max(vif_term_table$adjusted_GVIF, na.rm = TRUE),
    max(vif_column_table$VIF, na.rm = TRUE),
    VIF_THRESHOLD,
    any(vif_term_table$meaningful_collinearity, na.rm = TRUE),
    any(vif_column_table$meaningful_collinearity, na.rm = TRUE)
  )
)

safe_write_csv(
  vif_term_table,
  file.path(REV_DIRS$results, "REVISION_VIF_GVIF_TERM_LEVEL.csv")
)
safe_write_csv(
  vif_column_table,
  file.path(REV_DIRS$results, "REVISION_VIF_MODEL_MATRIX_COLUMNS.csv")
)
safe_write_csv(
  vif_summary,
  file.path(REV_DIRS$results, "REVISION_VIF_SUMMARY.csv")
)

# ------------------------------------------------------------------------------
```

```
# 18.5 1999-2014限定时期：补齐M0、M1和M2
# ------------------------------------------------------------------------

cat("[REV 3/10] 补跑1999-2014限定时期M0、M1和M2模型……\n")

secondary_16_outcomes <- tibble::tribble(
  ~event_var,                      ~outcome_label,
  "event_cvd_composite_16y",       "Cardiovascular composite mortality (heart +
cerebrovascular)",
  "event_clrd_16y",                "Chronic lower respiratory disease mortality"
)

secondary_16_all_list <- list()

for (k in seq_len(nrow(secondary_16_outcomes))) {
  ev <- secondary_16_outcomes$event_var[k]
  ol <- secondary_16_outcomes$outcome_label[k]

  for (nm in c("M0_unadjusted", "M1_demographic", "M2_core")) {
    secondary_16_all_list[[length(secondary_16_all_list) + 1]] <- run_svycox(
      design = design_16,
      time_var = "time_int_years",
      event_var = ev,
      rhs = RHS_MODELS[[nm]],
      analysis_label = paste0("Secondary restricted 1999-2014 / ", nm),
      outcome_label = ol
    )
  }
}

secondary_16_all_models <- bind_rows(secondary_16_all_list) %>%
  mutate(
    model = model_label_from_analysis(analysis),
    HR_95CI = format_hr(HR, CI_low, CI_high),
    p_formatted = format_p(p)
  )

safe_write_csv(
  secondary_16_all_models,
  file.path(
    REV_DIRS$results,
    "REVISION_MODEL_secondary_1999_2014_M0_M1_M2.csv"
  )
)

# ------------------------------------------------------------------------
# 18.6 心脏病死亡调查加权生存曲线
# ------------------------------------------------------------------------

cat("[REV 4/10] 生成心脏病死亡调查加权生存曲线……\n")
```

```
# 默认不计算svykm标准误，以避免se=TRUE在事件较多时产生过高内存负担。
# 审稿人要求的是Kaplan-Meier曲线本身，并未要求置信带。
KM_COMPUTE_SE <- FALSE

km_design <- complete_design(
  design_primary,
  c(
    "time_int_years",
    "event_heart",
    "exposure_f"
  )
)

km_fit <- survey::svykm(
  survival::Surv(time_int_years, event_heart) ~ exposure_f,
  design = km_design,
  se = KM_COMPUTE_SE
)

saveRDS(
  km_fit,
  file.path(REV_DIRS$results, "REVISION_weighted_heart_disease_svykm_object.rds")
)

if (inherits(km_fit, "svykmlist") || (is.list(km_fit) && length(km_fit) > 1)) {
  km_names <- names(km_fit)
  if (is.null(km_names)) km_names <- paste0("Group ", seq_along(km_fit))

  km_source_data <- purrr::map_dfr(seq_along(km_fit), function(i) {
    group_name <- km_names[i]
    group_name <- sub("^exposure_f=", "", group_name)
    group_name <- sub("^exposure_f", "", group_name)
    group_name <- trimws(group_name)
    extract_svykm_curve(km_fit[[i]], group_name)
  })
} else {
  km_source_data <- extract_svykm_curve(
    km_fit,
    "All participants"
  )
}

km_source_data <- km_source_data %>%
  mutate(
    exposure_group = dplyr::case_when(
      grepl("Persistent", exposure_group, ignore.case = TRUE) ~ "Persistent smoker",
      grepl("Post", exposure_group, ignore.case = TRUE) |
        grepl("quitter", exposure_group, ignore.case = TRUE) ~ "Post-diagnostic
quitter",
```

```
      TRUE ~ exposure_group
    ),
    exposure_group = factor(
      exposure_group,
      levels = c(
        "Persistent smoker",
        "Post-diagnostic quitter"
      )
    )
  ) %>%
  arrange(exposure_group, time_years)

safe_write_csv(
  km_source_data,
  file.path(
    REV_DIRS$source_data,
    "Figure2A_weighted_heart_disease_survival_source_data.csv"
  )
)

km_group_counts <- km_design$variables %>%
  count(exposure_f, name = "unweighted_n") %>%
  mutate(
    exposure_group = as.character(exposure_f)
  ) %>%
  select(exposure_group, unweighted_n)

safe_write_csv(
  km_group_counts,
  file.path(
    REV_DIRS$source_data,
    "Figure2A_weighted_survival_group_counts.csv"
  )
)

# ------------------------------------------------------------------------------
# 18.7 Table 1返修版：定义ASD、阈值和可识别不平衡
# ------------------------------------------------------------------------------

cat("[REV 5/10] 重构Table 1和Table 2……\n")

table1_revision <- main_table1 %>%
  mutate(
    section = case_when(
      characteristic %in% c(
        "Age, years",
        "NHANES survey midyear",
        "Years from first cardiopulmonary diagnosis to NHANES",
        "Regular-smoking duration before diagnosis, years",
        "Sex",
```

```
        "Race and ethnicity",
        "Education",
        "Cardiopulmonary disease phenotype"
      ) ~ "Core model variables",

      characteristic %in% c(
        "Cigarettes per day proxy",
        "Pack-years proxy"
      ) ~ "Smoking-burden variables",

      TRUE ~ "Additional descriptive/sensitivity variables"
    ),
    ASD_threshold = 0.10,
    meaningful_imbalance =
      absolute_standardized_difference >= ASD_threshold,
    ASD_display = ifelse(
      meaningful_imbalance,
      paste0(
        sprintf("%.3f", absolute_standardized_difference),
        " *"
      ),
      sprintf("%.3f", absolute_standardized_difference)
    )
  ) %>%
  select(
    section,
    characteristic,
    level,
    persistent_smokers,
    postdiagnostic_quitters,
    absolute_standardized_difference,
    ASD_display,
    meaningful_imbalance
  )

safe_write_csv(
  table1_revision,
  file.path(REV_DIRS$tables, "Table1_REVISED_baseline_characteristics.csv")
)

# Table 2：Panel A和Panel B均横向展示M0、M1、M2。
primary_table2_long <- main_models_df %>%
  filter(status == "ok") %>%
  mutate(
    panel = "Panel A: NHANES 1999-2018",
    model = model_label_from_analysis(analysis),
    HR_95CI = format_hr(HR, CI_low, CI_high),
    p_formatted = format_p(p)
  )
```

```
secondary_table2_long <- secondary_16_all_models %>%
  filter(status == "ok") %>%
  mutate(
    panel = "Panel B: NHANES 1999–2014"
  )

table2_long_revision <- bind_rows(
  primary_table2_long,
  secondary_table2_long
) %>%
  select(
    panel,
    outcome,
    model,
    n,
    events,
    n_quit,
    n_persistent,
    HR,
    CI_low,
    CI_high,
    p,
    HR_95CI,
    p_formatted
  )

table2_wide_revision <- table2_long_revision %>%
  select(
    panel,
    outcome,
    n,
    events,
    model,
    HR_95CI,
    p_formatted
  ) %>%
  tidyr::pivot_wider(
    names_from = model,
    values_from = c(HR_95CI, p_formatted),
    names_glue = "{model} | {.value}"
  ) %>%
  arrange(panel, outcome)

safe_write_csv(
  table2_long_revision,
  file.path(REV_DIRS$tables, "Table2_REVISED_long_model_results.csv")
)
safe_write_csv(
  table2_wide_revision,
  file.path(REV_DIRS$tables, "Table2_REVISED_wide_primary_and_restricted.csv")
```

```
)

# Excel工作簿:便于Word排版和审稿回复核对。
revision_workbook <- openxlsx::createWorkbook()

openxlsx::addWorksheet(revision_workbook, "Table1")
openxlsx::writeData(revision_workbook, "Table1", table1_revision)

openxlsx::addWorksheet(revision_workbook, "Table2_Wide")
openxlsx::writeData(revision_workbook, "Table2_Wide", table2_wide_revision)

openxlsx::addWorksheet(revision_workbook, "Table2_Long")
openxlsx::writeData(revision_workbook, "Table2_Long", table2_long_revision)

openxlsx::addWorksheet(revision_workbook, "VIF_GVIF")
openxlsx::writeData(revision_workbook, "VIF_GVIF", vif_term_table)

header_style <- openxlsx::createStyle(
  textDecoration = "bold",
  halign = "center",
  valign = "center",
  border = "Bottom"
)

for (sheet_name in c("Table1", "Table2_Wide", "Table2_Long", "VIF_GVIF")) {
  sheet_data <- switch(
    sheet_name,
    Table1 = table1_revision,
    Table2_Wide = table2_wide_revision,
    Table2_Long = table2_long_revision,
    VIF_GVIF = vif_term_table
  )

  openxlsx::addStyle(
    revision_workbook,
    sheet = sheet_name,
    style = header_style,
    rows = 1,
    cols = seq_len(ncol(sheet_data)),
    gridExpand = TRUE
  )

  openxlsx::setColWidths(
    revision_workbook,
    sheet = sheet_name,
    cols = seq_len(ncol(sheet_data)),
    widths = "auto"
  )

  openxlsx::freezePane(
```

```
    revision_workbook,
    sheet = sheet_name,
    firstRow = TRUE
  )
}

# Table 1中ASD≥0.10的ASD单元格加粗。
table1_bold_rows <- which(table1_revision$meaningful_imbalance) + 1
if (length(table1_bold_rows) > 0) {
  asd_col <- which(names(table1_revision) == "ASD_display")
  openxlsx::addStyle(
    revision_workbook,
    sheet = "Table1",
    style = openxlsx::createStyle(textDecoration = "bold"),
    rows = table1_bold_rows,
    cols = asd_col,
    gridExpand = TRUE,
    stack = TRUE
  )
}

openxlsx::saveWorkbook(
  revision_workbook,
  file.path(REV_DIRS$tables, "TID_MAJOR_REVISION_TABLES.xlsx"),
  overwrite = TRUE
)

# ---------------------------------------------------------------------------
# 18.8 Figure 1返修版:标准流程图 + 周期组成
# ---------------------------------------------------------------------------

cat("[REV 6/10] 生成Figure 1标准流程图和Figure 2返修复合图……\n")

# 最后一个重复的“Final domain”节点不再单独显示;
# 第9个节点直接作为最终分析样本,并加入两组人数。
flow_data <- cohort_flow %>%
  slice(1:9) %>%
  mutate(
    display_order = row_number(),
    y = rev(seq_len(n())),
    label = paste0(
      stringr::str_wrap(step, width = 37),
      "\n",
      "n = ",
      scales::comma(n)
    )
  )

flow_data$label[nrow(flow_data)] <- paste0(
  "Final primary analytic sample",
```

```
  "\n",
  "n = ", scales::comma(nrow(primary_df)),
  "\n",
  "Post-diagnostic quitters: ",
  scales::comma(sum(primary_df$exposure_quit == 1)),
  "; persistent smokers: ",
  scales::comma(sum(primary_df$exposure_quit == 0))
)

exclusion_reasons <- c(
  "Age <40 years",
  "No selected cardiopulmonary disease",
  "Missing/invalid first diagnosis age",
  "Never smoker or smoking began after diagnosis",
  "Not classifiable as smoking at diagnosis",
  "Exposure group not classifiable",
  "Mortality-linkage ineligible or no positive follow-up",
  "Invalid combined interview weight/design variables"
)

exclusion_data <- tibble(
  y = (flow_data$y[-nrow(flow_data)] +
         flow_data$y[-1]) / 2,
  excluded_n = flow_data$excluded_from_previous[-1],
  reason = exclusion_reasons,
  label = paste0(
    "Excluded: ",
    scales::comma(excluded_n),
    "\n",
    stringr::str_wrap(reason, width = 29)
  )
)

arrow_data <- tibble(
  x = 0.38,
  xend = 0.38,
  y = flow_data$y[-nrow(flow_data)] - 0.35,
  yend = flow_data$y[-1] + 0.35
)

p_revision_flow <- ggplot() +
  geom_rect(
    data = flow_data,
    aes(
      xmin = 0.04,
      xmax = 0.72,
      ymin = y - 0.35,
      ymax = y + 0.35
    ),
    fill = "white",
```

```
  color = "black",
  linewidth = 0.75
) +
geom_text(
  data = flow_data,
  aes(
    x = 0.38,
    y = y,
    label = label
  ),
  family = BASE_FAMILY,
  size = 3.25,
  lineheight = 0.95
) +
geom_segment(
  data = arrow_data,
  aes(
    x = x,
    xend = xend,
    y = y,
    yend = yend
  ),
  arrow = grid::arrow(
    length = grid::unit(0.12, "inches"),
    type = "closed"
  ),
  linewidth = 0.65
) +
geom_text(
  data = exclusion_data,
  aes(
    x = 0.78,
    y = y,
    label = label
  ),
  hjust = 0,
  family = BASE_FAMILY,
  size = 2.85,
  lineheight = 0.95
) +
coord_cartesian(
  xlim = c(0, 1.27),
  ylim = c(0.45, max(flow_data$y) + 0.50),
  clip = "off"
) +
theme_void(base_family = BASE_FAMILY) +
theme(
  plot.margin = margin(
    t = 8,
    r = 35,
```

```
      b = 8,
      l = 8
    )
  )

fig1b_revision_data <- analytic_cycle_audit %>%
  select(
    cycle,
    begin_year,
    n_postdiagnostic_quitters,
    n_persistent_smokers
  ) %>%
  pivot_longer(
    cols = c(
      n_postdiagnostic_quitters,
      n_persistent_smokers
    ),
    names_to = "exposure_group",
    values_to = "n"
  ) %>%
  mutate(
    exposure_group = dplyr::recode(
      exposure_group,
      n_postdiagnostic_quitters = "Post-diagnostic quitter",
      n_persistent_smokers = "Persistent smoker"
    ),
    cycle = factor(
      cycle,
      levels = analytic_cycle_audit$cycle
    )
  )

p_revision_cycle <- ggplot(
  fig1b_revision_data,
  aes(
    x = cycle,
    y = n,
    fill = exposure_group
  )
) +
  geom_col(width = 0.72) +
  scale_fill_manual(
    values = c(
      "Persistent smoker" = PERSIST_COLOR,
      "Post-diagnostic quitter" = QUIT_COLOR
    )
  ) +
  scale_y_continuous(
    labels = scales::comma,
    expand = expansion(mult = c(0, 0.06))
```

```r
  ) +
  labs(
    x = "NHANES cycle",
    y = "Unweighted participants",
    fill = NULL
  ) +
  pub_theme(11.5) +
  theme(
    axis.text.x = element_text(
      angle = 42,
      hjust = 1
    ),
    legend.position = "top"
  )

figure1_revision <- p_revision_flow + p_revision_cycle +
  patchwork::plot_layout(widths = c(1.30, 1.00)) +
  patchwork::plot_annotation(
    tag_levels = "A",
    theme = theme(
      plot.tag = element_text(
        family = BASE_FAMILY,
        size = 16
      )
    )
  )

safe_write_csv(
  flow_data,
  file.path(REV_DIRS$source_data, "Figure1A_flowchart_nodes.csv")
)
safe_write_csv(
  exclusion_data,
  file.path(REV_DIRS$source_data, "Figure1A_flowchart_exclusions.csv")
)
safe_write_csv(
  fig1b_revision_data,
  file.path(REV_DIRS$source_data, "Figure1B_cycle_composition.csv")
)

save_revision_figure(
  figure1_revision,
  "Figure1_REVISED_participant_flow_and_cycle_composition",
  width = 13.0,
  height = 10.0
)

# ------------------------------------------------------------------------------
# 18.9 Figure 2返修版:加权生存曲线 + 主要/限定时期模型
# ------------------------------------------------------------------------------
```

```
p_revision_km <- ggplot(
  km_source_data,
  aes(
    x = time_years,
    y = survival_probability,
    color = exposure_group,
    group = exposure_group
  )
) +
  geom_step(linewidth = 1.05, direction = "hv") +
  scale_color_manual(
    values = c(
      "Persistent smoker" = PERSIST_COLOR,
      "Post-diagnostic quitter" = QUIT_COLOR
    ),
    drop = FALSE
  ) +
  scale_y_continuous(
    limits = c(0, 1),
    labels = scales::label_percent(accuracy = 1),
    expand = expansion(mult = c(0, 0.02))
  ) +
  scale_x_continuous(
    breaks = scales::breaks_pretty(n = 7),
    expand = expansion(mult = c(0, 0.02))
  ) +
  labs(
    x = "Years since NHANES interview",
    y = "Heart-disease-free survival",
    color = NULL
  ) +
  pub_theme(11.5) +
  theme(
    legend.position = "top",
    panel.grid.minor = element_blank()
  )

primary_forest_data <- main_models_df %>%
  filter(status == "ok") %>%
  mutate(
    model = factor(
      model_label_from_analysis(analysis),
      levels = rev(c(
        "M0: Unadjusted",
        "M1: Demographic-adjusted",
        "M2: Fully adjusted core model"
      ))
    ),
    outcome = factor(
```

```
      outcome,
      levels = c(
        "All-cause mortality",
        "Heart-disease mortality",
        "Cancer mortality"
      )
    )
  )

p_revision_primary <- ggplot(
  primary_forest_data,
  aes(
    x = HR,
    y = model
  )
) +
  geom_vline(
    xintercept = 1,
    linetype = "dashed",
    linewidth = 0.75
  ) +
  geom_errorbarh(
    aes(
      xmin = CI_low,
      xmax = CI_high,
      color = model
    ),
    height = 0,
    linewidth = 0.95
  ) +
  geom_point(
    aes(color = model),
    size = 3.0
  ) +
  facet_grid(. ~ outcome) +
  scale_color_manual(
    values = c(
      "M0: Unadjusted" = "#9CA3AF",
      "M1: Demographic-adjusted" = "#6B7280",
      "M2: Fully adjusted core model" = QUIT_COLOR
    ),
    guide = "none"
  ) +
  scale_x_log10(
    breaks = c(0.3, 0.5, 0.7, 1, 1.5, 2),
    labels = scales::label_number(accuracy = 0.1)
  ) +
  coord_cartesian(
    xlim = c(0.35, 1.80),
    clip = "off"
```

```
  ) +
  labs(
    x = "Hazard ratio (log scale)",
    y = NULL
  ) +
  pub_theme(10.5) +
  theme(
    strip.text = element_text(size = 10.5),
    axis.text.y = element_text(size = 9.5),
    panel.spacing.x = grid::unit(0.20, "in")
  )

secondary_forest_data <- secondary_16_all_models %>%
  filter(status == "ok") %>%
  mutate(
    model = factor(
      model,
      levels = rev(c(
        "M0: Unadjusted",
        "M1: Demographic-adjusted",
        "M2: Fully adjusted core model"
      ))
    ),
    outcome_short = dplyr::recode(
      outcome,
      "Cardiovascular composite mortality (heart + cerebrovascular)" =
        "Cardiovascular composite",
      "Chronic lower respiratory disease mortality" =
        "Chronic lower respiratory disease"
    ),
    outcome_short = factor(
      outcome_short,
      levels = c(
        "Cardiovascular composite",
        "Chronic lower respiratory disease"
      )
    )
  )

p_revision_secondary <- ggplot(
  secondary_forest_data,
  aes(
    x = HR,
    y = model
  )
) +
  geom_vline(
    xintercept = 1,
    linetype = "dashed",
    linewidth = 0.75
```

```
  ) +
  geom_errorbarh(
    aes(
      xmin = CI_low,
      xmax = CI_high,
      color = model
    ),
    height = 0,
    linewidth = 0.95
  ) +
  geom_point(
    aes(color = model),
    size = 3.0
  ) +
  facet_grid(. ~ outcome_short) +
  scale_color_manual(
    values = c(
      "M0: Unadjusted" = "#9CA3AF",
      "M1: Demographic-adjusted" = "#6B7280",
      "M2: Fully adjusted core model" = QUIT_COLOR
    ),
    guide = "none"
  ) +
  scale_x_log10(
    breaks = c(0.3, 0.5, 0.7, 1, 1.5, 2, 3),
    labels = scales::label_number(accuracy = 0.1)
  ) +
  coord_cartesian(
    xlim = c(0.30, 2.30),
    clip = "off"
  ) +
  labs(
    x = "Hazard ratio (log scale)",
    y = NULL
  ) +
  pub_theme(10.5) +
  theme(
    strip.text = element_text(size = 10.3),
    axis.text.y = element_text(size = 9.5),
    panel.spacing.x = grid::unit(0.20, "in")
  )

figure2_revision <- p_revision_km /
  (p_revision_primary + p_revision_secondary) +
  patchwork::plot_layout(
    heights = c(1.00, 1.25)
  ) +
  patchwork::plot_annotation(
    tag_levels = "A",
    theme = theme(
```

```
      plot.tag = element_text(
        family = BASE_FAMILY,
        size = 16
      )
    )
  )

safe_write_csv(
  primary_forest_data,
  file.path(REV_DIRS$source_data, "Figure2B_primary_models.csv")
)
safe_write_csv(
  secondary_forest_data,
  file.path(REV_DIRS$source_data, "Figure2C_restricted_models.csv")
)

save_revision_figure(
  figure2_revision,
  "Figure2_REVISED_weighted_survival_and_mortality_models",
  width = 13.0,
  height = 10.2
)

# 将既有Figure 3和Figure 4复制到返修最终图目录。
for (stem in c(
  "Figure3_landmark_and_sensitivity_analyses",
  "Figure4_overlap_balance_and_subgroups"
)) {
  for (ext in c("tiff", "pdf")) {
    copy_if_exists(
      file.path(DIRS$figures, paste0(stem, ".", ext)),
      file.path(REV_DIRS$figures, paste0(stem, "_REVISED.", ext))
    )
  }
}

# ------------------------------------------------------------------------------
# 18.10 更新完整模型结果、补充表编号和回复信就绪材料
# ------------------------------------------------------------------------------

cat("[REV 7/10] 更新完整模型结果和补充表编号……\n")

all_model_results_revision <- bind_rows(
  main_models_df,
  secondary_16_all_models,
  sensitivity_results_df,
  overlap_results,
  subgroup_results_df,
  quit_age_sensitivity_models
) %>%
```

```
  mutate(
    log_ci_width = ifelse(
      is.finite(CI_low) &
        is.finite(CI_high) &
        CI_low > 0,
      log(CI_high) - log(CI_low),
      NA_real_
    ),
    qc_suspiciously_narrow_ci = as.integer(
      is.finite(log_ci_width) &
        log_ci_width < 0.10 &
        events < 500
    ),
    qc_extreme_p = as.integer(
      is.finite(p) &
        p < 1e-20
    ),
    HR_95CI = format_hr(HR, CI_low, CI_high),
    p_formatted = format_p(p)
  )

safe_write_csv(
  all_model_results_revision,
  file.path(
    REV_DIRS$results,
    "REVISION_ALL_MODEL_RESULTS_WITH_QC_FLAGS.csv"
  )
)

# 推荐的补充表新编号：
# S1在Study population首先引用；S2-S5用于Methods；
# S6以后用于Results，避免再次出现S11先于S8的情况。
supplement_mapping <- tibble::tribble(
  ~old_file, ~new_file, ~content,
  "Table_S1_cohort_flow.csv",
  "Table_S1_cohort_construction.csv",
  "Cohort construction",

  "Table_S2_baseline_continuous_source.csv",
  "Table_S2_continuous_baseline_variables.csv",
  "Continuous baseline variables",

  "Table_S3_baseline_categorical_source.csv",
  "Table_S3_categorical_baseline_variables.csv",
  "Categorical baseline variables",

  "Table_S9_missingness_primary_dataset.csv",
  "Table_S4_missingness_primary_dataset.csv",
  "Missingness",
```

```
  "REVISION_VIF_GVIF_TERM_LEVEL.csv",
  "Table_S5_multicollinearity_diagnostics.csv",
  "VIF/GVIF diagnostics",

  "Table_S4_outcome_counts_and_cycles.csv",
  "Table_S6_outcome_counts_and_cycles.csv",
  "Outcome counts and cycle availability",

  "Table_S5_quit_age_discrepancy_distribution.csv",
  "Table_S7_quit_age_discrepancy_distribution.csv",
  "Quit-age discrepancy distribution",

  "Table_S6_quit_age_sensitivity_sample_counts.csv",
  "Table_S8_quit_age_sensitivity_sample_counts.csv",
  "Quit-age sensitivity sample counts",

  "Table_S7_quit_age_discrepancy_by_cycle.csv",
  "Table_S9_quit_age_discrepancy_by_cycle.csv",
  "Quit-age discrepancy by cycle",

  "REVISION_ALL_MODEL_RESULTS_WITH_QC_FLAGS.csv",
  "Table_S10_all_model_results_with_QC_flags.csv",
  "Complete model results",

  "Table_S10_overlap_weighting_covariate_balance.csv",
  "Table_S11_overlap_weighting_covariate_balance.csv",
  "Overlap-weighting balance",

  "Table_S12_interaction_test_details.csv",
  "Table_S12_interaction_test_details.csv",
  "Interaction tests",

  "Table_S8_full_proportional_hazards_tests.csv",
  "Table_S13_full_proportional_hazards_tests.csv",
  "Proportional hazards diagnostics"
)

for (i in seq_len(nrow(supplement_mapping))) {
  old_name <- supplement_mapping$old_file[i]
  new_name <- supplement_mapping$new_file[i]

  if (grepl("^REVISION_VIF", old_name)) {
    from_path <- file.path(REV_DIRS$results, old_name)
  } else if (grepl("^REVISION_ALL_MODEL", old_name)) {
    from_path <- file.path(REV_DIRS$results, old_name)
  } else {
    from_path <- file.path(DIRS$supp_tables, old_name)
  }

  to_path <- file.path(REV_DIRS$supplementary, new_name)
```

```
  copy_if_exists(from_path, to_path)
}

safe_write_csv(
  supplement_mapping,
  file.path(
    REV_DIRS$supplementary,
    "Supplementary_Table_Renumbering_Map.csv"
  )
)

# --------------------------------------------------------------------------------
# 18.11 动态生成可用于逐点回复信的统计文本
# --------------------------------------------------------------------------------

cat("[REV 8/10] 生成审稿回复就绪的统计摘要……\n")

max_term_gvif <- max(
  vif_term_table$adjusted_GVIF,
  na.rm = TRUE
)
max_column_vif <- max(
  vif_column_table$VIF,
  na.rm = TRUE
)

vif_interpretation <- if (
  is.finite(max_term_gvif) &&
  max_term_gvif < VIF_THRESHOLD
) {
  paste0(
    "No meaningful multicollinearity was identified. ",
    "The maximum adjusted GVIF was ",
    sprintf("%.2f", max_term_gvif),
    ", below the prespecified threshold of ",
    VIF_THRESHOLD,
    "."
  )
} else {
  paste0(
    "At least one adjusted GVIF reached the prespecified threshold of ",
    VIF_THRESHOLD,
    ". The affected terms should be reviewed before finalizing the response."
  )
}

secondary_response_rows <- secondary_16_all_models %>%
  filter(
    status == "ok",
    model == "M2: Fully adjusted core model"
```

```
  ) %>%
  mutate(
    response_line = paste0(
      outcome,
      ": HR ",
      sprintf("%.2f", HR),
      " (95% CI ",
      sprintf("%.2f", CI_low),
      "–",
      sprintf("%.2f", CI_high),
      "; P ",
      format_p(p),
      ")."
    )
  )

response_ready_lines <- c(
  "TID-02054-2026-01 — STATISTICAL RESPONSE-READY SUMMARY",
  paste0("Generated: ", format(Sys.time())),
  "",
  "1. Multicollinearity",
  paste0(
    "We assessed multicollinearity using variance inflation factors based on the
same covariate design matrix used in the fully adjusted core model. ",
    vif_interpretation
  ),
  paste0(
    "The maximum individual model-matrix column VIF was ",
    sprintf("%.2f", max_column_vif),
    "."
  ),
  "",
  "2. Survey-weighted survival curve",
  paste0(
    "A survey-weighted heart-disease-free survival curve was generated for ",
    scales::comma(nrow(km_design$variables)),
    " participants (",
    scales::comma(sum(km_design$variables$exposure_quit == 1)),
    " post-diagnostic quitters and ",
    scales::comma(sum(km_design$variables$exposure_quit == 0)),
    " persistent smokers). The curve is descriptive; the adjusted association
remains based on survey-weighted Cox regression."
  ),
  "",
  "3. Restricted-period models (NHANES 1999–2014)",
  secondary_response_rows$response_line,
  "",
  "4. Core result reproduced",
  paste0(
    "The fully adjusted association with heart-disease mortality was reproduced: HR
```

```
",
    sprintf("%.2f", heart_m2_check$HR),
    " (95% CI ",
    sprintf("%.2f", heart_m2_check$CI_low),
    "–",
    sprintf("%.2f", heart_m2_check$CI_high),
    "; P ",
    format_p(heart_m2_check$p),
    "), with persistent smokers as the reference group."
  ),
  "",
  "5. Interpretation boundary",
  "All Kaplan–Meier and Cox results are observational associations. They do not
establish a causal effect of smoking cessation because diagnosis and smoking
histories were reconstructed among survivors to NHANES enrollment."
)

write_lines_utf8(
  response_ready_lines,
  file.path(
    REV_DIRS$response,
    "STATISTICAL_RESPONSE_READY_TEXT.txt"
  )
)

figure_table_heading_templates <- c(
  "RECOMMENDED STAND-ALONE HEADINGS",
  "",
  paste0(
    "Figure 1. Participant selection and survey-cycle composition in a secondary
observational analysis of US NHANES 1999–2018 data linked to mortality follow-up
through 2019 (final analytic sample, n=",
    scales::comma(nrow(primary_df)),
    ")."
  ),
  "",
  paste0(
    "Figure 2. Survey-weighted heart-disease-free survival and survey-weighted Cox
model estimates comparing post-diagnostic quitters with persistent smokers in US
NHANES 1999–2018 linked to mortality follow-up through 2019 (n=",
    scales::comma(nrow(primary_df)),
    ")."
  ),
  "",
  paste0(
    "Table 1. Baseline characteristics by diagnosis-anchored smoking status in a
secondary observational analysis of US NHANES 1999–2018 linked to mortality
follow-up through 2019 (n=",
    scales::comma(nrow(primary_df)),
    "). Values are survey-weighted means (SD) or unweighted counts (survey-weighted
```

```
percentages). An absolute standardized difference ≥0.10 indicates meaningful
baseline imbalance."
  ),
  "",
  paste0(
    "Table 2. Survey-weighted Cox estimates for mortality outcomes in a secondary
observational analysis of US NHANES linked-mortality data. Panel A includes NHANES
1999–2018 (n=",
    scales::comma(nrow(primary_df)),
    "); Panel B includes NHANES 1999–2014 (n=",
    scales::comma(nrow(design_16$variables)),
    "). Hazard ratios compare post-diagnostic quitters with persistent smokers. M0
was unadjusted; M1 adjusted for age, sex, race and ethnicity, and education; M2
additionally adjusted for disease phenotype, years since diagnosis, years of smoking
before diagnosis, and survey cycle."
  )
)

write_lines_utf8(
  figure_table_heading_templates,
  file.path(
    REV_DIRS$response,
    "FIGURE_TABLE_HEADING_TEMPLATES.txt"
  )
)

# ---------------------------------------------------------------------------
# 18.12 返修运行说明、文件清单和审计
# ---------------------------------------------------------------------------

cat("[REV 9/10] 生成运行说明、文件清单和审计(不生成压缩包)……\n")

run_instructions <- c(
  "TID-02054-2026-01 MAJOR REVISION COMPLETE R SCRIPT V2",
  "",
  "Required folder layout:",
  "C:/Users/33652/Desktop/Q/",
  "  CARDIOPULMONARY_SMOKING_REANALYSIS_FINAL_1999_2018/",
  "    00_raw_data/",
  "      NHANES/",
  "      NHANES_mortality/",
  "",
  "This script writes all new outputs to:",
  "C:/Users/33652/Desktop/Q/TID_MAJOR_REVISION_FINAL_ALL_IN_ONE/",
  "",
  "Do not delete or rename the locked source project before running.",
  "No new NHANES research data are downloaded.",
  "R packages may be installed automatically when missing.",
  "",
  "Primary completion criteria:",
```

```
  "1. Core reproducibility check passes.",
  "2. VIF/GVIF files are generated.",
  "3. Survey-weighted heart-disease survival source data and Figure 2 are
generated.",
  "4. Restricted-period M0/M1/M2 results are generated.",
  "5. Revised Table 1, Table 2, Figure 1 and Figure 2 are generated.",
  "6. REVISION_PUBLICATION_READY_STATUS.csv reports PASS."
)

write_lines_utf8(
  run_instructions,
  file.path(REV_DIRS$root, "README_RUN_INSTRUCTIONS.txt")
)

# 保存当前完整脚本。
current_revision_source <- tryCatch(
  normalizePath(
    sys.frame(1)$ofile,
    winslash = "/",
    mustWork = FALSE
  ),
  error = function(e) NA_character_
)

if (
  is.character(current_revision_source) &&
  length(current_revision_source) == 1 &&
  !is.na(current_revision_source) &&
  file.exists(current_revision_source)
) {
  file.copy(
    current_revision_source,
    file.path(
      REV_DIRS$code,
      "TID_MAJOR_REVISION_FINAL_ALL_IN_ONE_COMPLETE_RUN_v1.R"
    ),
    overwrite = TRUE
  )
}

required_revision_files <- c(
  file.path(REV_DIRS$audit, "REVISION_CORE_REPRODUCIBILITY_CHECK.csv"),
  file.path(REV_DIRS$results, "REVISION_VIF_GVIF_TERM_LEVEL.csv"),
  file.path(REV_DIRS$results, "REVISION_MODEL_secondary_1999_2014_M0_M1_M2.csv"),
  file.path(REV_DIRS$tables, "Table1_REVISED_baseline_characteristics.csv"),
  file.path(REV_DIRS$tables, "Table2_REVISED_wide_primary_and_restricted.csv"),
  file.path(REV_DIRS$figures,
"Figure1_REVISED_participant_flow_and_cycle_composition.tiff"),
  file.path(REV_DIRS$figures,
"Figure2_REVISED_weighted_survival_and_mortality_models.tiff"),
```

```
  file.path(REV_DIRS$response, "STATISTICAL_RESPONSE_READY_TEXT.txt")
)

revision_file_audit <- tibble(
  path = required_revision_files,
  exists = file.exists(required_revision_files),
  size_bytes = ifelse(
    file.exists(required_revision_files),
    file.info(required_revision_files)$size,
    NA_real_
  )
)

safe_write_csv(
  revision_file_audit,
  file.path(REV_DIRS$audit, "REVISION_REQUIRED_FILE_AUDIT.csv")
)

publication_ready_status <- tibble(
  check = c(
    "Core result reproducibility",
    "VIF/GVIF diagnostics completed",
    "Restricted-period M0/M1/M2 completed",
    "Weighted survival curve completed",
    "Revised Table 1 completed",
    "Revised Table 2 completed",
    "Revised Figure 1 completed",
    "Revised Figure 2 completed",
    "All required files present"
  ),
  pass = c(
    all(expected_check$pass),
    file.exists(file.path(REV_DIRS$results, "REVISION_VIF_GVIF_TERM_LEVEL.csv")),
    all(secondary_16_all_models$status == "ok"),
    nrow(km_source_data) > 0,
    file.exists(file.path(REV_DIRS$tables,
"Table1_REVISED_baseline_characteristics.csv")),
    file.exists(file.path(REV_DIRS$tables,
"Table2_REVISED_wide_primary_and_restricted.csv")),
    file.exists(file.path(REV_DIRS$figures,
"Figure1_REVISED_participant_flow_and_cycle_composition.tiff")),
    file.exists(file.path(REV_DIRS$figures,
"Figure2_REVISED_weighted_survival_and_mortality_models.tiff")),
    all(revision_file_audit$exists)
  )
) %>%
  mutate(
    status = ifelse(pass, "PASS", "FAIL")
  )
```

```
safe_write_csv(
  publication_ready_status,
  file.path(REV_DIRS$root, "REVISION_PUBLICATION_READY_STATUS.csv")
)

capture.output(
  sessionInfo(),
  file = file.path(
    REV_DIRS$logs,
    paste0("sessionInfo_revision_", REVISION_STAMP, ".txt")
  )
)

revision_manifest_files <- list.files(
  REVISION_ROOT,
  recursive = TRUE,
  full.names = TRUE
)

revision_manifest <- tibble(
  relative_path = fs::path_rel(
    revision_manifest_files,
    start = REVISION_ROOT
  ),
  size_bytes = file.info(revision_manifest_files)$size,
  modified_time = as.character(
    file.info(revision_manifest_files)$mtime
  )
)

safe_write_csv(
  revision_manifest,
  file.path(REV_DIRS$root, "REVISION_FILE_MANIFEST.csv")
)

# 按用户要求:返修模块不生成ZIP压缩包。
revision_zip_generation_status <- "DISABLED"

# ------------------------------------------------------------------------------
# 18.13 最终状态
# ------------------------------------------------------------------------------

cat("[REV 10/10] 完成最终状态检查……\n")

if (!all(publication_ready_status$pass)) {
  failed_items <- publication_ready_status %>%
    filter(!pass) %>%
    pull(check)

  warning(
```

```r
    "返修模块尚未全部通过:",
    paste(failed_items, collapse = "; ")
  )
} else {
  cat("\nPUBLICATION-READY REVISION ANALYSIS PACKAGE COMPLETED\n")
}

cat("\n================================================================\n")
cat("TID大修新增分析完成:", format(Sys.time()), "\n", sep = "")
cat("返修结果目录:", REVISION_ROOT, "\n", sep = "")
cat("检查压缩包:未生成(按用户要求)\n")
cat("最大调整GVIF:", sprintf("%.3f", max_term_gvif), "\n", sep = "")
cat(
  "心脏病死亡M2:HR ",
  sprintf("%.3f", heart_m2_check$HR),
  " (95% CI ",
  sprintf("%.3f", heart_m2_check$CI_low),
  "-",
  sprintf("%.3f", heart_m2_check$CI_high),
  ")\n",
  sep = ""
)
cat(
  "最终状态:",
  ifelse(
    all(publication_ready_status$pass),
    "PASS",
    "FAIL"
  ),
  "\n",
  sep = ""
)
cat("================================================================\n")

sink()

################################################################################
# 19. INTEGRATED REVIEWER-SUPPLEMENTAL ANALYSIS
################################################################################

################################################################################
# 项目:TID-02054-2026-01 大修补充分析
# 集成模块:共线性敏感性分析 + 比例风险诊断修正版
# 版本:INTEGRATED_SUPPLEMENTAL_COLLINEARITY_PH_FINAL_v2_2026-07-17
#
# V2关键修正:
# - 删除survey::svycoxph中的ties参数;V1的9个共线性敏感性模型因此报错;
# - 在生成汇总前强制检查9个模型全部成功,禁止NA/Inf结果进入回复文本;
# - 状态表明确区分3个复现模型和6个新增leave-one-covariate-out模型。
#
```

```
# 目的：
# 1) 针对核心模型中中度共线性变量，补做leave-one-covariate-out敏感性分析；
# 2) 重新计算并规范解释VIF/GVIF；
# 3) 使用terms=TRUE、singledf=FALSE重新生成term-level cox.zph结果；
# 4) 输出可直接用于补充表和逐点回复信的结果文件；
# 5) 不重新下载任何数据，不改变主分析样本和主模型定义。
#
# 运行前提：
# 已完整运行：
# NHANES_1999_2018_TID_MAJOR_REVISION_COMPLETE_FINAL_v2.R
#
# 输入：
# C:/Users/33652/Desktop/Q/TID_MAJOR_REVISION_FINAL_ALL_IN_ONE/
#   02_processed_data/02_analysis_all_derived.rds
#   04_results/MODEL_primary_survey_weighted_cox_models.csv
#
# 输出：
# C:/Users/33652/Desktop/Q/TID_MAJOR_REVISION_FINAL_ALL_IN_ONE/
#   11_SUPPLEMENTAL_COLLINEARITY_PH_ANALYSIS/
################################################################################

options(stringsAsFactors = FALSE)
options(warn = 1)
options(timeout = 1200)
options(survey.lonely.psu = "adjust")

# ==============================================================================
# 0. 用户设置
# ==============================================================================

# 复用上方主流程已经定义的PROJECT_PARENT和PROJECT_ROOT。
if (!exists("PROJECT_PARENT") || !exists("PROJECT_ROOT")) {
  stop("未检测到主流程PROJECT_PARENT/PROJECT_ROOT；请从本完整脚本第一行开始运行。")
}

INPUT_ANALYSIS_RDS <- file.path(
  PROJECT_ROOT,
  "02_processed_data",
  "02_analysis_all_derived.rds"
)

INPUT_PRIMARY_MODELS <- file.path(
  PROJECT_ROOT,
  "04_results",
  "MODEL_primary_survey_weighted_cox_models.csv"
)

OUTPUT_ROOT <- file.path(
  PROJECT_ROOT,
  "11_SUPPLEMENTAL_COLLINEARITY_PH_ANALYSIS"
```

```
)

SUPP_DIRS <- list(
  root = OUTPUT_ROOT,
  results = file.path(OUTPUT_ROOT, "01_results"),
  tables = file.path(OUTPUT_ROOT, "02_tables"),
  response = file.path(OUTPUT_ROOT, "03_response_ready"),
  audit = file.path(OUTPUT_ROOT, "04_audit"),
  logs = file.path(OUTPUT_ROOT, "05_logs")
)

AUTO_INSTALL_PACKAGES <- TRUE
VIF_MODERATE_THRESHOLD <- 5
VIF_SERIOUS_THRESHOLD <- 10
MATERIAL_LOGHR_CHANGE_THRESHOLD <- 0.10

invisible(lapply(SUPP_DIRS, dir.create, recursive = TRUE, showWarnings = FALSE))

if (!file.exists(INPUT_ANALYSIS_RDS)) {
  stop(
    "未找到分析数据:\n",
    INPUT_ANALYSIS_RDS,
    "\n请先完整运行主返修代码V2。"
  )
}

if (!file.exists(INPUT_PRIMARY_MODELS)) {
  stop(
    "未找到主模型结果:\n",
    INPUT_PRIMARY_MODELS,
    "\n请先完整运行主返修代码V2。"
  )
}

# ============================================================================
# 1. 安装并载入R包
# ============================================================================

required_packages <- c(
  "dplyr",
  "tidyr",
  "purrr",
  "readr",
  "tibble",
  "survey",
  "survival",
  "car",
  "openxlsx"
)
```

```
installed_now <- rownames(installed.packages())

if (AUTO_INSTALL_PACKAGES) {
  for (pkg in required_packages) {
    if (!pkg %in% installed_now) {
      try(
        install.packages(
          pkg,
          repos = "https://cloud.r-project.org",
          dependencies = TRUE
        ),
        silent = TRUE
      )
    }
  }
}

missing_packages <- required_packages[
  !vapply(
    required_packages,
    requireNamespace,
    logical(1),
    quietly = TRUE
  )
]

if (length(missing_packages) > 0) {
  stop(
    "缺少必需R包：",
    paste(missing_packages, collapse = ", ")
  )
}

suppressPackageStartupMessages({
  library(dplyr)
  library(tidyr)
  library(purrr)
  library(readr)
  library(tibble)
  library(survey)
  library(survival)
})

# 不附加car、openxlsx或zip，全部使用显式命名空间，避免函数屏蔽。

RUN_STAMP <- format(Sys.time(), "%Y%m%d_%H%M%S")
LOG_FILE <- file.path(
  SUPP_DIRS$logs,
  paste0("supplemental_collinearity_PH_log_", RUN_STAMP, ".txt")
)
```

```r
sink(LOG_FILE, split = TRUE)
on.exit({
  while (sink.number() > 0) sink()
}, add = TRUE)

cat("============================================================\n")
cat("TID SUPPLEMENTAL COLLINEARITY AND PH ANALYSIS\n")
cat("Started: ", format(Sys.time()), "\n", sep = "")
cat("Project root: ", PROJECT_ROOT, "\n", sep = "")
cat("Output root: ", OUTPUT_ROOT, "\n", sep = "")
cat("Research-data download: DISABLED\n")
cat("============================================================\n\n")

# ============================================================================
# 2. 通用函数
# ============================================================================

format_hr <- function(hr, lo, hi) {
  ifelse(
    is.na(hr),
    NA_character_,
    sprintf("%.2f (%.2f-%.2f)", hr, lo, hi)
  )
}

format_p <- function(p) {
  ifelse(
    is.na(p),
    NA_character_,
    ifelse(p < 0.001, "<0.001", sprintf("%.3f", p))
  )
}

safe_write_csv <- function(x, path) {
  readr::write_csv(as.data.frame(x), path, na = "")
}

write_lines_utf8 <- function(x, path) {
  con <- file(path, open = "wt", encoding = "UTF-8")
  on.exit(close(con), add = TRUE)
  writeLines(x, con = con, useBytes = TRUE)
}

complete_design <- function(design, vars) {
  vars <- unique(vars)
  vars <- vars[vars %in% names(design$variables)]

  ok <- stats::complete.cases(
    design$variables[, vars, drop = FALSE]
```

```
  )

  design$variables$.complete_model_internal <- ok
  out <- base::subset(
    design,
    .complete_model_internal
  )
  out$variables <- droplevels(out$variables)
  out
}

extract_svycox_term <- function(
    fit,
    term = "exposure_quit"
) {
  b <- stats::coef(fit)
  V <- stats::vcov(fit)

  if (!term %in% names(b)) {
    return(tibble(
      term = term,
      logHR = NA_real_,
      SE = NA_real_,
      HR = NA_real_,
      CI_low = NA_real_,
      CI_high = NA_real_,
      p = NA_real_
    ))
  }

  se <- sqrt(V[term, term])
  z <- b[term] / se

  tibble(
    term = term,
    logHR = unname(b[term]),
    SE = unname(se),
    HR = exp(unname(b[term])),
    CI_low = exp(
      unname(b[term]) -
        stats::qnorm(0.975) * se
    ),
    CI_high = exp(
      unname(b[term]) +
        stats::qnorm(0.975) * se
    ),
    p = 2 * stats::pnorm(
      abs(z),
      lower.tail = FALSE
    )
```

```
  )
}

run_svycox <- function(
    design,
    time_var,
    event_var,
    rhs,
    analysis_label,
    outcome_label,
    term = "exposure_quit"
) {
  needed <- unique(c(
    time_var,
    event_var,
    all.vars(
      stats::as.formula(
        paste0("~", rhs)
      )
    )
  ))

  des <- complete_design(
    design,
    needed
  )

  n_model <- nrow(des$variables)
  n_events <- sum(
    des$variables[[event_var]] == 1,
    na.rm = TRUE
  )
  n_quit <- sum(
    des$variables$exposure_quit == 1,
    na.rm = TRUE
  )
  n_persistent <- sum(
    des$variables$exposure_quit == 0,
    na.rm = TRUE
  )

  base_result <- tibble(
    analysis = analysis_label,
    outcome = outcome_label,
    time_variable = time_var,
    event_variable = event_var,
    formula_rhs = rhs,
    model_type = "survey::svycoxph",
    n = n_model,
    events = n_events,
```

```r
  n_quit = n_quit,
  n_persistent = n_persistent,
  design_df = tryCatch(
    survey::degf(des),
    error = function(e) NA_real_
  )
)

if (
  n_model == 0 ||
    n_events < 10 ||
    n_quit == 0 ||
    n_persistent == 0
) {
  return(
    dplyr::bind_cols(
      base_result,
      tibble(
        term = term,
        logHR = NA_real_,
        SE = NA_real_,
        HR = NA_real_,
        CI_low = NA_real_,
        CI_high = NA_real_,
        p = NA_real_,
        status = "not_run",
        error = "样本、事件数或暴露组不足"
      )
    )
  )
}

model_formula <- stats::as.formula(
  paste0(
    "survival::Surv(",
    time_var,
    ", ",
    event_var,
    ") ~ ",
    rhs
  )
)

tryCatch({
  fit <- suppressWarnings(
    survey::svycoxph(
      model_formula,
      design = des
    )
  )
```

```
    estimate <- extract_svycox_term(
      fit,
      term
    )

    dplyr::bind_cols(
      base_result,
      estimate
    ) %>%
      mutate(
        status = if_else(
          is.finite(HR),
          "ok",
          "failed"
        ),
        error = NA_character_
      )
  }, error = function(e) {
    dplyr::bind_cols(
      base_result,
      tibble(
        term = term,
        logHR = NA_real_,
        SE = NA_real_,
        HR = NA_real_,
        CI_low = NA_real_,
        CI_high = NA_real_,
        p = NA_real_,
        status = "failed",
        error = conditionMessage(e)
      )
    )
  })
}

make_column_vif <- function(model_matrix) {
  mm <- as.matrix(model_matrix)

  if ("(Intercept)" %in% colnames(mm)) {
    mm <- mm[
      ,
      colnames(mm) != "(Intercept)",
      drop = FALSE
    ]
  }

  finite_columns <- vapply(
    seq_len(ncol(mm)),
    function(j) {
```

```
    x <- mm[, j]
    all(is.finite(x)) &&
      stats::sd(x) > 0
  },
  logical(1)
)

mm <- mm[
  ,
  finite_columns,
  drop = FALSE
]

if (ncol(mm) < 2) {
  return(tibble(
    model_matrix_column = colnames(mm),
    VIF = NA_real_,
    tolerance = NA_real_
  ))
}

result <- lapply(
  seq_len(ncol(mm)),
  function(j) {
    y <- mm[, j]
    x <- mm[, -j, drop = FALSE]

    fit <- tryCatch(
      stats::lm.fit(
        x = cbind(
          "(Intercept)" = 1,
          x
        ),
        y = y
      ),
      error = function(e) e
    )

    if (inherits(fit, "error")) {
      return(tibble(
        model_matrix_column = colnames(mm)[j],
        VIF = NA_real_,
        tolerance = NA_real_
      ))
    }

    rss <- sum(fit$residuals^2)
    tss <- sum(
      (y - mean(y))^2
    )
```

```
      r_squared <- ifelse(
        tss > 0,
        1 - rss / tss,
        NA_real_
      )

      if (is.finite(r_squared)) {
        r_squared <- min(
          max(r_squared, 0),
          0.999999999
        )
      }

      vif_value <- ifelse(
        is.finite(r_squared),
        1 / (1 - r_squared),
        NA_real_
      )

      tibble(
        model_matrix_column = colnames(mm)[j],
        VIF = vif_value,
        tolerance = ifelse(
          is.finite(vif_value),
          1 / vif_value,
          NA_real_
        )
      )
    }
  )

  bind_rows(result) %>%
    arrange(desc(VIF))
}

classify_standard_vif <- function(vif_value) {
  dplyr::case_when(
    !is.finite(vif_value) ~ "Unavailable",
    vif_value > VIF_SERIOUS_THRESHOLD ~
      "Potentially serious multicollinearity",
    vif_value >= VIF_MODERATE_THRESHOLD ~
      "Moderate multicollinearity",
    TRUE ~
      "No meaningful multicollinearity"
  )
}

extract_zph_table <- function(
    zph_object,
```

```
    outcome_label,
    n_model,
    events,
    table_type
) {
  tb <- as.data.frame(zph_object$table)
  tb$term <- rownames(tb)
  rownames(tb) <- NULL

  tibble(
    outcome = outcome_label,
    n = n_model,
    events = events,
    table_type = table_type,
    term = tb$term,
    chisq = if (
      "chisq" %in% names(tb)
    ) {
      tb$chisq
    } else {
      NA_real_
    },
    df = if (
      "df" %in% names(tb)
    ) {
      tb$df
    } else {
      NA_real_
    },
    p = if (
      "p" %in% names(tb)
    ) {
      tb$p
    } else {
      NA_real_
    },
    status = "ok",
    error = NA_character_
  )
}

# ==============================================================================
# 3. 读取数据并重建与主分析一致的复杂抽样设计
# ==============================================================================

cat("[1/8] 读取锁定分析数据并重建复杂抽样设计……\n")

analysis_all <- readRDS(
  INPUT_ANALYSIS_RDS
)
```

```r
required_variables <- c(
  "age",
  "eligstat",
  "permth_int",
  "wt_int_20y",
  "sdmvpsu",
  "sdmvstra",
  "survey_psu_uid",
  "survey_strata_uid",
  "domain_primary",
  "time_int_years",
  "event_allcause",
  "event_heart",
  "event_cancer",
  "exposure_quit",
  "age10",
  "sex_f",
  "race_f",
  "education_f",
  "disease_f",
  "years_since_dx5",
  "pre_dx_smoking_years10",
  "survey_cycle_2y_centered"
)

missing_variables <- setdiff(
  required_variables,
  names(analysis_all)
)

if (length(missing_variables) > 0) {
  stop(
    "分析数据缺少变量:",
    paste(missing_variables, collapse = ", ")
  )
}

base_int_20 <- analysis_all %>%
  filter(
    age >= 18,
    eligstat == 1,
    is.finite(permth_int),
    permth_int > 0,
    is.finite(wt_int_20y),
    wt_int_20y > 0,
    is.finite(sdmvpsu),
    is.finite(sdmvstra)
  )
```

```
design_int_20_full <- survey::svydesign(
  ids = ~survey_psu_uid,
  strata = ~survey_strata_uid,
  weights = ~wt_int_20y,
  nest = TRUE,
  data = base_int_20
)

design_primary <- base::subset(
  design_int_20_full,
  domain_primary == 1
)

design_primary$variables <- droplevels(
  design_primary$variables
)

primary_df <- design_primary$variables %>%
  droplevels()

# ===============================================================================
# 4. 核心样本硬性核验
# ===============================================================================

cat("[2/8] 核验核心样本和事件数……\n")

core_check <- tibble(
  metric = c(
    "Primary analysis n",
    "Post-diagnostic quitters",
    "Persistent smokers",
    "All-cause deaths",
    "Heart-disease deaths",
    "Cancer deaths"
  ),
  expected = c(
    2319,
    975,
    1344,
    999,
    272,
    244
  ),
  actual = c(
    nrow(primary_df),
    sum(primary_df$exposure_quit == 1),
    sum(primary_df$exposure_quit == 0),
    sum(primary_df$event_allcause == 1),
    sum(primary_df$event_heart == 1),
    sum(primary_df$event_cancer == 1)
```

```r
  )
) %>%
  mutate(
    pass = expected == actual
  )

safe_write_csv(
  core_check,
  file.path(
    SUPP_DIRS$audit,
    "SUPPLEMENTAL_CORE_SAMPLE_CHECK.csv"
  )
)

if (!all(core_check$pass)) {
  stop(
    "核心样本核验未通过，请停止分析并检查输入文件。"
  )
}

# ==============================================================================
# 5. 重新计算VIF/GVIF并采用规范阈值解释
# ==============================================================================

cat("[3/8] 重新计算并规范解释VIF/GVIF……\n")

RHS_FULL <- paste(
  "exposure_quit + age10 + sex_f + race_f + education_f +",
  "disease_f + years_since_dx5 + pre_dx_smoking_years10 +",
  "survey_cycle_2y_centered"
)

vif_variables <- unique(c(
  "time_int_years",
  all.vars(
    stats::as.formula(
      paste0("~", RHS_FULL)
    )
  )
))

vif_data <- primary_df %>%
  select(all_of(vif_variables)) %>%
  filter(stats::complete.cases(.)) %>%
  droplevels()

vif_lm_fit <- stats::lm(
  stats::as.formula(
    paste0(
      "time_int_years ~ ",
```

```
      RHS_FULL
    )
  ),
  data = vif_data,
  na.action = stats::na.fail
)

vif_raw <- car::vif(
  vif_lm_fit
)

if (is.matrix(vif_raw)) {
  vif_term_table <- as.data.frame(
    vif_raw
  ) %>%
    tibble::rownames_to_column("term")

  names(vif_term_table) <- gsub(
    "\\s+",
    "_",
    names(vif_term_table)
  )

  gvif_col <- grep(
    "^GVIF$",
    names(vif_term_table),
    value = TRUE
  )

  df_col <- grep(
    "^Df$|^DF$",
    names(vif_term_table),
    value = TRUE
  )

  adjusted_col <- grep(
    "GVIF",
    names(vif_term_table),
    value = TRUE
  )

  adjusted_col <- setdiff(
    adjusted_col,
    gvif_col
  )

  vif_term_table <- vif_term_table %>%
    mutate(
      GVIF_raw = .data[[gvif_col[1]]],
      Df_term = .data[[df_col[1]]],
```

```r
      GVIF_adjusted = .data[[adjusted_col[1]]],
      equivalent_standard_VIF_for_1df =
        ifelse(
          Df_term == 1,
          GVIF_raw,
          NA_real_
        ),
      adjusted_threshold_equivalent_to_VIF5 =
        sqrt(VIF_MODERATE_THRESHOLD),
      adjusted_threshold_equivalent_to_VIF10 =
        sqrt(VIF_SERIOUS_THRESHOLD),
      interpretation = dplyr::case_when(
        Df_term == 1 &
          GVIF_raw > VIF_SERIOUS_THRESHOLD ~
          "Potentially serious multicollinearity",

        Df_term == 1 &
          GVIF_raw >= VIF_MODERATE_THRESHOLD ~
          "Moderate multicollinearity",

        Df_term == 1 ~
          "No meaningful multicollinearity",

        Df_term > 1 &
          GVIF_adjusted >
          sqrt(VIF_SERIOUS_THRESHOLD) ~
          "Potentially serious multicollinearity",

        Df_term > 1 &
          GVIF_adjusted >=
          sqrt(VIF_MODERATE_THRESHOLD) ~
          "Moderate multicollinearity",

        TRUE ~
          "No meaningful multicollinearity"
      )
    )
} else {
  vif_term_table <- tibble(
    term = names(vif_raw),
    GVIF_raw = as.numeric(vif_raw),
    Df_term = 1,
    GVIF_adjusted = sqrt(
      as.numeric(vif_raw)
    ),
    equivalent_standard_VIF_for_1df =
      as.numeric(vif_raw),
    adjusted_threshold_equivalent_to_VIF5 =
      sqrt(VIF_MODERATE_THRESHOLD),
    adjusted_threshold_equivalent_to_VIF10 =
```

```
      sqrt(VIF_SERIOUS_THRESHOLD),
    interpretation = classify_standard_vif(
      as.numeric(vif_raw)
    )
  )
}

vif_model_matrix <- stats::model.matrix(
  stats::as.formula(
    paste0("~", RHS_FULL)
  ),
  data = vif_data
)

vif_column_table <- make_column_vif(
  vif_model_matrix
) %>%
  mutate(
    moderate_threshold = VIF_MODERATE_THRESHOLD,
    serious_threshold = VIF_SERIOUS_THRESHOLD,
    interpretation = classify_standard_vif(VIF)
  )

continuous_correlation <- vif_data %>%
  select(
    age10,
    years_since_dx5,
    pre_dx_smoking_years10,
    survey_cycle_2y_centered
  ) %>%
  stats::cor(
    use = "pairwise.complete.obs",
    method = "pearson"
  ) %>%
  as.data.frame() %>%
  tibble::rownames_to_column("variable")

max_column_vif <- max(
  vif_column_table$VIF,
  na.rm = TRUE
)

n_moderate_columns <- sum(
  vif_column_table$VIF >=
    VIF_MODERATE_THRESHOLD &
    vif_column_table$VIF <=
    VIF_SERIOUS_THRESHOLD,
  na.rm = TRUE
)
```

```r
n_serious_columns <- sum(
  vif_column_table$VIF >
    VIF_SERIOUS_THRESHOLD,
  na.rm = TRUE
)

vif_summary <- tibble(
  diagnostic = c(
    "Complete cases used",
    "Maximum individual model-matrix VIF",
    "Number of columns with VIF 5–10",
    "Number of columns with VIF >10",
    "Moderate threshold",
    "Potentially serious threshold"
  ),
  value = c(
    nrow(vif_data),
    max_column_vif,
    n_moderate_columns,
    n_serious_columns,
    VIF_MODERATE_THRESHOLD,
    VIF_SERIOUS_THRESHOLD
  )
)

safe_write_csv(
  vif_term_table,
  file.path(
    SUPP_DIRS$results,
    "COLLINEARITY_term_level_GVIF_CORRECTED.csv"
  )
)

safe_write_csv(
  vif_column_table,
  file.path(
    SUPP_DIRS$results,
    "COLLINEARITY_model_matrix_VIF_CORRECTED.csv"
  )
)

safe_write_csv(
  continuous_correlation,
  file.path(
    SUPP_DIRS$results,
    "COLLINEARITY_core_continuous_correlations.csv"
  )
)

safe_write_csv(
```

```
  vif_summary,
  file.path(
    SUPP_DIRS$results,
    "COLLINEARITY_summary_CORRECTED.csv"
  )
)

# ============================================================================
# 6. Leave-one-covariate-out共线性敏感性分析
# ============================================================================

cat("[4/8] 运行leave-one-covariate-out共线性敏感性模型……\n")

RHS_SENSITIVITY <- list(
  M2_full = RHS_FULL,

  M2_without_years_since_diagnosis = paste(
    "exposure_quit + age10 + sex_f + race_f + education_f +",
    "disease_f + pre_dx_smoking_years10 +",
    "survey_cycle_2y_centered"
  ),

  M2_without_prediagnosis_smoking_duration = paste(
    "exposure_quit + age10 + sex_f + race_f + education_f +",
    "disease_f + years_since_dx5 +",
    "survey_cycle_2y_centered"
  )
)

main_outcomes <- tibble::tribble(
  ~event_var,       ~outcome_label,
  "event_allcause", "All-cause mortality",
  "event_heart",    "Heart-disease mortality",
  "event_cancer",   "Cancer mortality"
)

sensitivity_model_list <- list()

for (k in seq_len(nrow(main_outcomes))) {
  event_variable <- main_outcomes$event_var[k]
  outcome_label <- main_outcomes$outcome_label[k]

  for (
    model_name in names(RHS_SENSITIVITY)
  ) {
    sensitivity_model_list[[
      length(sensitivity_model_list) + 1
    ]] <- run_svycox(
      design = design_primary,
      time_var = "time_int_years",
```

```
      event_var = event_variable,
      rhs = RHS_SENSITIVITY[[model_name]],
      analysis_label = model_name,
      outcome_label = outcome_label
    )
  }
}

collinearity_sensitivity_results <- bind_rows(
  sensitivity_model_list
) %>%
  mutate(
    HR_95CI = format_hr(
      HR,
      CI_low,
      CI_high
    ),
    p_formatted = format_p(p)
  )

successful_sensitivity_models <- sum(
  collinearity_sensitivity_results$status == "ok"
)

if (successful_sensitivity_models != 9) {
  failed_sensitivity_models <- collinearity_sensitivity_results %>%
    filter(status != "ok") %>%
    transmute(
      label = paste0(
        outcome,
        " / ",
        analysis,
        ": ",
        error
      )
    ) %>%
    pull(label)

  stop(
    "共线性敏感性模型未全部成功。成功数：",
    successful_sensitivity_models,
    "/9。失败模型：",
    paste(
      failed_sensitivity_models,
      collapse = " | "
    )
  )
}

primary_locked_results <- readr::read_csv(
```

```
  INPUT_PRIMARY_MODELS,
  show_col_types = FALSE,
  progress = FALSE
) %>%
  filter(
    grepl(
      "M2_core$",
      analysis
    )
  ) %>%
  select(
    outcome,
    locked_HR = HR,
    locked_CI_low = CI_low,
    locked_CI_high = CI_high,
    locked_p = p
  )

collinearity_sensitivity_comparison <-
  collinearity_sensitivity_results %>%
  left_join(
    primary_locked_results,
    by = "outcome"
  ) %>%
  mutate(
    absolute_HR_change_from_locked =
      abs(HR - locked_HR),

    relative_HR_change_percent =
      100 * (
        HR / locked_HR - 1
      ),

    absolute_logHR_change =
      abs(
        log(HR) -
          log(locked_HR)
      ),

    material_logHR_change =
      absolute_logHR_change >
        MATERIAL_LOGHR_CHANGE_THRESHOLD,

    same_direction_as_locked =
      sign(log(HR)) ==
        sign(log(locked_HR)),

    statistical_significance_at_0_05 =
      p < 0.05
  )
```

```
heart_sensitivity_summary <-
  collinearity_sensitivity_comparison %>%
  filter(
    outcome ==
      "Heart-disease mortality",
    status == "ok",
    is.finite(HR),
    is.finite(CI_low),
    is.finite(CI_high),
    is.finite(p)
  ) %>%
  summarise(
    minimum_HR = min(
      HR,
      na.rm = TRUE
    ),
    maximum_HR = max(
      HR,
      na.rm = TRUE
    ),
    minimum_CI_low = min(
      CI_low,
      na.rm = TRUE
    ),
    maximum_CI_high = max(
      CI_high,
      na.rm = TRUE
    ),
    all_same_direction =
      all(
        same_direction_as_locked,
        na.rm = TRUE
      ),
    all_p_below_0_05 =
      all(
        p < 0.05,
        na.rm = TRUE
      ),
    any_material_logHR_change =
      any(
        material_logHR_change,
        na.rm = TRUE
      )
  )

safe_write_csv(
  collinearity_sensitivity_results,
  file.path(
    SUPP_DIRS$results,
```

```
    "COLLINEARITY_leave_one_covariate_out_models.csv"
  )
)

safe_write_csv(
  collinearity_sensitivity_comparison,
  file.path(
    SUPP_DIRS$results,
    "COLLINEARITY_sensitivity_vs_locked_primary.csv"
  )
)

safe_write_csv(
  heart_sensitivity_summary,
  file.path(
    SUPP_DIRS$results,
    "COLLINEARITY_heart_mortality_summary.csv"
  )
)

# ============================================================================
# 7. 比例风险假设修正版
# ============================================================================

cat("[5/8] 重新生成term-level和coefficient-level比例风险诊断……\n")

PH_RHS <- RHS_FULL
ph_term_list <- list()
ph_coefficient_list <- list()
ph_objects <- list()

for (k in seq_len(nrow(main_outcomes))) {
  event_variable <- main_outcomes$event_var[k]
  outcome_label <- main_outcomes$outcome_label[k]

  needed <- unique(c(
    "time_int_years",
    event_variable,
    all.vars(
      stats::as.formula(
        paste0("~", PH_RHS)
      )
    )
  ))

  des_ph <- complete_design(
    design_primary,
    needed
  )
```

```r
formula_ph <- stats::as.formula(
  paste0(
    "survival::Surv(time_int_years, ",
    event_variable,
    ") ~ ",
    PH_RHS
  )
)

fit_ph <- tryCatch(
  suppressWarnings(
    survey::svycoxph(
      formula_ph,
      design = des_ph,
      x = TRUE,
      y = TRUE,
      model = TRUE
    )
  ),
  error = function(e) e
)

if (inherits(fit_ph, "error")) {
  failure_row <- tibble(
    outcome = outcome_label,
    n = nrow(des_ph$variables),
    events = sum(
      des_ph$variables[[event_variable]] == 1,
      na.rm = TRUE
    ),
    table_type = NA_character_,
    term = NA_character_,
    chisq = NA_real_,
    df = NA_real_,
    p = NA_real_,
    status = "failed",
    error = conditionMessage(fit_ph)
  )

  ph_term_list[[
    length(ph_term_list) + 1
  ]] <- failure_row

  ph_coefficient_list[[
    length(ph_coefficient_list) + 1
  ]] <- failure_row
} else {
  zph_term <- tryCatch(
    survival::cox.zph(
      fit_ph,
```

```
    transform = "km",
    terms = TRUE,
    singledf = FALSE,
    global = TRUE
  ),
  error = function(e) e
)

zph_coefficient <- tryCatch(
  survival::cox.zph(
    fit_ph,
    transform = "km",
    terms = FALSE,
    global = TRUE
  ),
  error = function(e) e
)

if (inherits(zph_term, "error")) {
  ph_term_list[[
    length(ph_term_list) + 1
  ]] <- tibble(
    outcome = outcome_label,
    n = nrow(des_ph$variables),
    events = sum(
      des_ph$variables[[event_variable]] == 1,
      na.rm = TRUE
    ),
    table_type = "term-level",
    term = NA_character_,
    chisq = NA_real_,
    df = NA_real_,
    p = NA_real_,
    status = "failed",
    error = conditionMessage(zph_term)
  )
} else {
  ph_term_list[[
    length(ph_term_list) + 1
  ]] <- extract_zph_table(
    zph_object = zph_term,
    outcome_label = outcome_label,
    n_model = nrow(des_ph$variables),
    events = sum(
      des_ph$variables[[event_variable]] == 1,
      na.rm = TRUE
    ),
    table_type = "term-level"
  )
}
```

```
if (inherits(zph_coefficient, "error")) {
  ph_coefficient_list[[
    length(ph_coefficient_list) + 1
  ]] <- tibble(
    outcome = outcome_label,
    n = nrow(des_ph$variables),
    events = sum(
      des_ph$variables[[event_variable]] == 1,
      na.rm = TRUE
    ),
    table_type = "coefficient-level",
    term = NA_character_,
    chisq = NA_real_,
    df = NA_real_,
    p = NA_real_,
    status = "failed",
    error = conditionMessage(
      zph_coefficient
    )
  )
} else {
  ph_coefficient_list[[
    length(ph_coefficient_list) + 1
  ]] <- extract_zph_table(
    zph_object = zph_coefficient,
    outcome_label = outcome_label,
    n_model = nrow(des_ph$variables),
    events = sum(
      des_ph$variables[[event_variable]] == 1,
      na.rm = TRUE
    ),
    table_type = "coefficient-level"
  )
}

ph_objects[[outcome_label]] <- list(
  fit = fit_ph,
  zph_term = if (
    inherits(zph_term, "error")
  ) {
    NULL
  } else {
    zph_term
  },
  zph_coefficient = if (
    inherits(
      zph_coefficient,
      "error"
    )
```

```
      ) {
        NULL
      } else {
        zph_coefficient
      }
    )
  }
}

ph_term_results <- bind_rows(
  ph_term_list
) %>%
  mutate(
    p_formatted = format_p(p)
  )

ph_coefficient_results <- bind_rows(
  ph_coefficient_list
) %>%
  mutate(
    p_formatted = format_p(p)
  )

ph_summary <- purrr::map_dfr(
  main_outcomes$outcome_label,
  function(outcome_label) {
    tmp <- ph_term_results %>%
      filter(
        outcome == outcome_label,
        status == "ok"
      )

    exposure_row <- tmp %>%
      filter(
        term == "exposure_quit"
      )

    global_row <- tmp %>%
      filter(
        toupper(term) == "GLOBAL"
      )

    exposure_p <- if (
      nrow(exposure_row) > 0
    ) {
      exposure_row$p[1]
    } else {
      NA_real_
    }
```

```
    global_p <- if (
      nrow(global_row) > 0
    ) {
      global_row$p[1]
    } else {
      NA_real_
    }

    tibble(
      outcome = outcome_label,
      n = if (
        nrow(tmp) > 0
      ) {
        tmp$n[1]
      } else {
        NA_integer_
      },
      events = if (
        nrow(tmp) > 0
      ) {
        tmp$events[1]
      } else {
        NA_integer_
      },
      exposure_ph_p = exposure_p,
      exposure_ph_p_formatted =
        format_p(exposure_p),
      global_ph_p = global_p,
      global_ph_p_formatted =
        format_p(global_p),
      exposure_conclusion =
        dplyr::case_when(
          !is.finite(exposure_p) ~
            "Assessment unavailable",
          exposure_p < 0.05 ~
            "Statistical evidence of non-proportionality for the smoking-cessation
coefficient",
          TRUE ~
            "No statistical evidence of non-proportionality for the
smoking-cessation coefficient"
        ),
      global_conclusion =
        dplyr::case_when(
          !is.finite(global_p) ~
            "Assessment unavailable",
          global_p < 0.05 ~
            "The global test suggests that at least one coefficient may vary over
time",
          TRUE ~
            "No statistical evidence of global non-proportionality"
```

```
      )
    )
  }
)

saveRDS(
  ph_objects,
  file.path(
    SUPP_DIRS$results,
    "PH_corrected_cox_zph_objects.rds"
  ),
  compress = "xz"
)

safe_write_csv(
  ph_term_results,
  file.path(
    SUPP_DIRS$results,
    "PH_term_level_CORRECTED.csv"
  )
)

safe_write_csv(
  ph_coefficient_results,
  file.path(
    SUPP_DIRS$results,
    "PH_coefficient_level_CORRECTED.csv"
  )
)

safe_write_csv(
  ph_summary,
  file.path(
    SUPP_DIRS$results,
    "PH_summary_CORRECTED.csv"
  )
)

# ==============================================================================
# 8. 生成补充表、Excel工作簿和回复信就绪文本
# ==============================================================================

cat("[6/8] 生成补充表和回复信就绪材料……\n")

table_s5_corrected <- vif_term_table %>%
  select(
    term,
    GVIF_raw,
    Df_term,
    GVIF_adjusted,
```

```
    equivalent_standard_VIF_for_1df,
    interpretation
  )

table_s5b_sensitivity <-
  collinearity_sensitivity_comparison %>%
  select(
    outcome,
    analysis,
    n,
    events,
    HR,
    CI_low,
    CI_high,
    p,
    HR_95CI,
    p_formatted,
    locked_HR,
    absolute_HR_change_from_locked,
    relative_HR_change_percent,
    absolute_logHR_change,
    material_logHR_change,
    same_direction_as_locked
  )

table_s13_corrected <- ph_term_results %>%
  select(
    outcome,
    n,
    events,
    term,
    chisq,
    df,
    p,
    p_formatted,
    status,
    error
  )

safe_write_csv(
  table_s5_corrected,
  file.path(
    SUPP_DIRS$tables,
    "Table_S5_multicollinearity_diagnostics_CORRECTED.csv"
  )
)

safe_write_csv(
  table_s5b_sensitivity,
  file.path(
```

```
    SUPP_DIRS$tables,
    "Table_S5B_collinearity_sensitivity_models.csv"
  )
)

safe_write_csv(
  table_s13_corrected,
  file.path(
    SUPP_DIRS$tables,
    "Table_S13_proportional_hazards_tests_CORRECTED.csv"
  )
)

workbook <- openxlsx::createWorkbook()

sheet_data <- list(
  VIF_GVIF = table_s5_corrected,
  Column_VIF = vif_column_table,
  Collinearity_Sensitivity =
    table_s5b_sensitivity,
  PH_Term_Level = table_s13_corrected,
  PH_Coefficient_Level =
    ph_coefficient_results,
  PH_Summary = ph_summary,
  Core_Check = core_check
)

for (sheet_name in names(sheet_data)) {
  openxlsx::addWorksheet(
    workbook,
    sheet_name
  )

  openxlsx::writeData(
    workbook,
    sheet = sheet_name,
    x = sheet_data[[sheet_name]]
  )

  openxlsx::freezePane(
    workbook,
    sheet = sheet_name,
    firstRow = TRUE
  )

  openxlsx::setColWidths(
    workbook,
    sheet = sheet_name,
    cols = seq_len(
      ncol(sheet_data[[sheet_name]])
```

```
    ),
    widths = "auto"
  )

  openxlsx::addStyle(
    workbook,
    sheet = sheet_name,
    style = openxlsx::createStyle(
      textDecoration = "bold",
      halign = "center",
      valign = "center",
      border = "Bottom"
    ),
    rows = 1,
    cols = seq_len(
      ncol(sheet_data[[sheet_name]])
    ),
    gridExpand = TRUE
  )
}

openxlsx::saveWorkbook(
  workbook,
  file.path(
    SUPP_DIRS$tables,
    "TID_SUPPLEMENTAL_COLLINEARITY_PH_TABLES.xlsx"
  ),
  overwrite = TRUE
)

moderate_vif_rows <- vif_column_table %>%
  filter(
    VIF >= VIF_MODERATE_THRESHOLD,
    VIF <= VIF_SERIOUS_THRESHOLD
  )

serious_vif_rows <- vif_column_table %>%
  filter(
    VIF > VIF_SERIOUS_THRESHOLD
  )

heart_rows <- collinearity_sensitivity_results %>%
  filter(
    outcome ==
      "Heart-disease mortality"
  ) %>%
  arrange(analysis)

heart_response_lines <- heart_rows %>%
  mutate(
```

```
    response_line = paste0(
      analysis,
      ": HR ",
      sprintf("%.2f", HR),
      " (95% CI ",
      sprintf("%.2f", CI_low),
      "-",
      sprintf("%.2f", CI_high),
      "; P ",
      format_p(p),
      ")."
    )
  ) %>%
  pull(response_line)

vif_response_sentence <- paste0(
  "The maximum individual model-matrix VIF was ",
  sprintf("%.2f", max_column_vif),
  ". ",
  n_moderate_columns,
  " model-matrix column(s) had VIF values between 5 and 10, and ",
  n_serious_columns,
  " had VIF values above 10."
)

sensitivity_response_sentence <- if (
  nrow(heart_sensitivity_summary) == 1 &&
    isTRUE(
      heart_sensitivity_summary$all_same_direction
    ) &&
    !isTRUE(
      heart_sensitivity_summary$any_material_logHR_change
    )
) {
  paste0(
    "Leave-one-covariate-out sensitivity analyses produced materially similar
heart-disease mortality estimates, with HRs ranging from ",
    sprintf(
      "%.2f",
      heart_sensitivity_summary$minimum_HR
    ),
    " to ",
    sprintf(
      "%.2f",
      heart_sensitivity_summary$maximum_HR
    ),
    "."
  )
} else {
  paste0(
```

```
    "Leave-one-covariate-out estimates should be reviewed before describing them as
materially unchanged. The observed HR range was ",
    sprintf(
      "%.2f",
      heart_sensitivity_summary$minimum_HR
    ),
    " to ",
    sprintf(
      "%.2f",
      heart_sensitivity_summary$maximum_HR
    ),
    "."
  )
}

ph_response_lines <- ph_summary %>%
  mutate(
    response_line = paste0(
      outcome,
      ": exposure-specific P=",
      exposure_ph_p_formatted,
      "; global P=",
      global_ph_p_formatted,
      "."
    )
  ) %>%
  pull(response_line)

response_ready_text <- c(
  "TID-02054-2026-01 — SUPPLEMENTAL STATISTICAL RESPONSE-READY TEXT",
  paste0("Generated: ", format(Sys.time())),
  "",
  "1. Multicollinearity assessment",
  vif_response_sentence,
  "VIF values of 5–10 were interpreted as moderate multicollinearity, whereas values
above 10 were considered potentially serious.",
  "",
  "2. Leave-one-covariate-out sensitivity analyses",
  sensitivity_response_sentence,
  heart_response_lines,
  "",
  "3. Corrected proportional hazards diagnostics",
  "Term-level proportional hazards tests were recalculated using cox.zph with
terms=TRUE and singledf=FALSE so that multi-level factors retained their appropriate
degrees of freedom.",
  ph_response_lines,
  "",
  "4. Recommended interpretation boundary",
  "These analyses evaluate numerical stability and model diagnostics only. They do
not remove survivor selection, recall bias, reverse causation, exposure
```

```
misclassification, or residual confounding."
)

write_lines_utf8(
  response_ready_text,
  file.path(
    SUPP_DIRS$response,
    "SUPPLEMENTAL_RESPONSE_READY_TEXT.txt"
  )
)

methods_results_text <- c(
  "RECOMMENDED METHODS TEXT",
  "",
  "Multicollinearity was assessed using variance inflation factors based on the
covariate design matrix of the fully adjusted model. VIF values of 5–10 were
interpreted as moderate multicollinearity and values above 10 as potentially
serious. For multi-degree-of-freedom factors, both the generalized VIF and
GVIF^(1/[2×df]) were reported. Leave-one-covariate-out sensitivity models separately
omitted years since diagnosis and pre-diagnosis smoking duration.",
  "",
  "The proportional hazards assumption was assessed using scaled Schoenfeld
residuals. Term-level tests were calculated with terms=TRUE and singledf=FALSE,
preserving the degrees of freedom of multi-level categorical variables.
Exposure-specific and global P values were emphasized.",
  "",
  "RECOMMENDED RESULTS TEXT TEMPLATE",
  "",
  vif_response_sentence,
  sensitivity_response_sentence,
  paste(
    ph_response_lines,
    collapse = " "
  )
)

write_lines_utf8(
  methods_results_text,
  file.path(
    SUPP_DIRS$response,
    "SUPPLEMENTAL_METHODS_RESULTS_TEXT.txt"
  )
)

# ==============================================================================
# 9. 文件审计、最终状态和检查压缩包
# ==============================================================================

cat("[7/8] 生成文件审计和检查压缩包……\n")
```

```
required_output_files <- c(
  file.path(
    SUPP_DIRS$results,
    "COLLINEARITY_term_level_GVIF_CORRECTED.csv"
  ),
  file.path(
    SUPP_DIRS$results,
    "COLLINEARITY_model_matrix_VIF_CORRECTED.csv"
  ),
  file.path(
    SUPP_DIRS$results,
    "COLLINEARITY_leave_one_covariate_out_models.csv"
  ),
  file.path(
    SUPP_DIRS$results,
    "COLLINEARITY_sensitivity_vs_locked_primary.csv"
  ),
  file.path(
    SUPP_DIRS$results,
    "PH_term_level_CORRECTED.csv"
  ),
  file.path(
    SUPP_DIRS$results,
    "PH_summary_CORRECTED.csv"
  ),
  file.path(
    SUPP_DIRS$tables,
    "Table_S5_multicollinearity_diagnostics_CORRECTED.csv"
  ),
  file.path(
    SUPP_DIRS$tables,
    "Table_S5B_collinearity_sensitivity_models.csv"
  ),
  file.path(
    SUPP_DIRS$tables,
    "Table_S13_proportional_hazards_tests_CORRECTED.csv"
  ),
  file.path(
    SUPP_DIRS$response,
    "SUPPLEMENTAL_RESPONSE_READY_TEXT.txt"
  )
)

file_audit <- tibble(
  path = required_output_files,
  exists = file.exists(
    required_output_files
  ),
  size_bytes = ifelse(
    file.exists(required_output_files),
```

```
    file.info(
      required_output_files
    )$size,
    NA_real_
  )
)

safe_write_csv(
  file_audit,
  file.path(
    SUPP_DIRS$audit,
    "SUPPLEMENTAL_REQUIRED_FILE_AUDIT.csv"
  )
)

ph_success <- ph_summary %>%
  summarise(
    all_outcomes_available =
      n() == 3 &&
      all(is.finite(exposure_ph_p)) &&
      all(is.finite(global_ph_p))
  ) %>%
  pull(all_outcomes_available)

status_table <- tibble(
  check = c(
    "Core sample reproduced",
    "VIF/GVIF recalculated",
    "Three full-model reproductions completed",
    "Six leave-one-covariate-out models completed",
    "Corrected PH diagnostics completed",
    "No VIF above 10",
    "All required files present"
  ),
  pass = c(
    all(core_check$pass),
    nrow(vif_column_table) > 0,
    sum(
      collinearity_sensitivity_results$status == "ok" &
        collinearity_sensitivity_results$analysis == "M2_full"
    ) == 3,
    sum(
      collinearity_sensitivity_results$status == "ok" &
        collinearity_sensitivity_results$analysis != "M2_full"
    ) == 6,
    isTRUE(ph_success),
    n_serious_columns == 0,
    all(file_audit$exists)
  )
) %>%
```

```
  mutate(
    status = ifelse(
      pass,
      "PASS",
      "FAIL"
    )
  )

safe_write_csv(
  status_table,
  file.path(
    SUPP_DIRS$root,
    "SUPPLEMENTAL_ANALYSIS_STATUS.csv"
  )
)

capture.output(
  sessionInfo(),
  file = file.path(
    SUPP_DIRS$logs,
    paste0(
      "sessionInfo_supplemental_",
      RUN_STAMP,
      ".txt"
    )
  )
)

manifest_files <- list.files(
  OUTPUT_ROOT,
  recursive = TRUE,
  full.names = TRUE
)

manifest <- tibble(
  relative_path = sub(
    paste0(
      "^",
      gsub(
        "\\\\",
        "/",
        normalizePath(
          OUTPUT_ROOT,
          winslash = "/",
          mustWork = TRUE
        )
      ),
      "/?"
    ),
    "",
```

```r
    gsub(
      "\\\\",
      "/",
      normalizePath(
        manifest_files,
        winslash = "/",
        mustWork = FALSE
      )
    )
  ),
  size_bytes = file.info(
    manifest_files
  )$size,
  modified_time = as.character(
    file.info(
      manifest_files
    )$mtime
  )
)

safe_write_csv(
  manifest,
  file.path(
    SUPP_DIRS$root,
    "SUPPLEMENTAL_FILE_MANIFEST.csv"
  )
)

# 按用户要求:补充分析模块不生成ZIP压缩包。
supplemental_zip_generation_status <- "DISABLED"

# ============================================================================
# 10. 最终状态
# ============================================================================

cat("[8/8] 完成最终状态检查……\n")

if (!all(status_table$pass)) {
  failed_checks <- status_table %>%
    filter(!pass) %>%
    pull(check)

  warning(
    "补充分析存在未通过项目:",
    paste(
      failed_checks,
      collapse = "; "
    )
  )
} else {
```

```
  cat(
    "\nSUPPLEMENTAL COLLINEARITY AND PH ANALYSIS V2 COMPLETED — PASS\n"
  )
}

cat("\n=================================================================\n")
cat("Completed: ", format(Sys.time()), "\n", sep = "")
cat("Output root: ", OUTPUT_ROOT, "\n", sep = "")
cat("Check ZIP: not generated (disabled by user request)\n")
cat(
  "Maximum individual VIF: ",
  sprintf("%.3f", max_column_vif),
  "\n",
  sep = ""
)
cat(
  "Columns with VIF 5–10: ",
  n_moderate_columns,
  "\n",
  sep = ""
)
cat(
  "Columns with VIF >10: ",
  n_serious_columns,
  "\n",
  sep = ""
)
cat(
  "Heart-disease sensitivity HR range: ",
  sprintf(
    "%.3f",
    heart_sensitivity_summary$minimum_HR
  ),
  "–",
  sprintf(
    "%.3f",
    heart_sensitivity_summary$maximum_HR
  ),
  "\n",
  sep = ""
)
cat(
  "Final status: ",
  ifelse(
    all(status_table$pass),
    "PASS",
    "REVIEW REQUIRED"
  ),
  "\n",
  sep = ""
```

```
)
cat("============================================================\n")

sink()

################################################################################
# 20. FINAL FIGURE PACKAGE
#     Figure 1-4单面板600 dpi TIFF、拼图、源数据与最终审计
################################################################################

while (sink.number() > 0) sink()

FINAL_FIGURE_STAMP <- format(Sys.time(), "%Y%m%d_%H%M%S")
FINAL_FIGURE_ROOT <- file.path(PROJECT_ROOT, "12_FINAL_FIGURE_PACKAGE")

FINAL_FIG_DIRS <- list(
  root = FINAL_FIGURE_ROOT,
  panels = file.path(FINAL_FIGURE_ROOT, "01_individual_panels_TIFF"),
  combined = file.path(FINAL_FIGURE_ROOT, "02_combined_figures"),
  source_data = file.path(FINAL_FIGURE_ROOT, "03_source_data"),
  reviewer_data = file.path(FINAL_FIGURE_ROOT, "04_reviewer_analysis_source_data"),
  audit = file.path(FINAL_FIGURE_ROOT, "05_audit"),
  code = file.path(FINAL_FIGURE_ROOT, "06_code"),
  logs = file.path(FINAL_FIGURE_ROOT, "07_logs")
)

invisible(lapply(FINAL_FIG_DIRS, fs::dir_create))

FINAL_FIGURE_LOG <- file.path(
  FINAL_FIG_DIRS$logs,
  paste0("final_figure_package_log_", FINAL_FIGURE_STAMP, ".txt")
)

sink(FINAL_FIGURE_LOG, split = TRUE)

cat("\n============================================================\n")
cat("FINAL FIGURE PACKAGE: INDIVIDUAL PANELS + COMBINED FIGURES\n")
cat("Started: ", format(Sys.time()), "\n", sep = "")
cat("Output root: ", FINAL_FIGURE_ROOT, "\n", sep = "")
cat("TIFF resolution: ", DPI, " dpi\n", sep = "")
cat("ZIP generation: DISABLED\n")
cat("============================================================\n\n")

required_final_objects <- c(
  "flow_data",
  "exclusion_data",
  "fig1b_revision_data",
  "p_revision_flow",
  "p_revision_cycle",
  "km_source_data",
```

```
  "km_group_counts",
  "primary_forest_data",
  "secondary_forest_data",
  "fig3a_data",
  "balance_results",
  "subgroup_results_df",
  "main_models_df",
  "sensitivity_results_df",
  "overlap_results",
  "quit_age_sensitivity_models",
  "collinearity_sensitivity_results",
  "vif_term_table",
  "vif_column_table",
  "ph_term_results",
  "ph_summary"
)

missing_final_objects <- required_final_objects[
  !vapply(
    required_final_objects,
    exists,
    logical(1),
    inherits = TRUE
  )
]

if (length(missing_final_objects) > 0) {
  stop(
    "最终绘图模块缺少对象:",
    paste(missing_final_objects, collapse = ", "),
    "\n请确认从本完整脚本第一行开始运行。"
  )
}

save_panel_tiff <- function(plot, stem, width, height) {
  ggplot2::ggsave(
    filename = file.path(
      FINAL_FIG_DIRS$panels,
      paste0(stem, ".tiff")
    ),
    plot = plot,
    width = width,
    height = height,
    units = "in",
    dpi = DPI,
    device = "tiff",
    compression = "lzw",
    limitsize = FALSE
  )
}
```

```
save_combined_figure <- function(plot, stem, width, height) {
  ggplot2::ggsave(
    filename = file.path(
      FINAL_FIG_DIRS$combined,
      paste0(stem, ".tiff")
    ),
    plot = plot,
    width = width,
    height = height,
    units = "in",
    dpi = DPI,
    device = "tiff",
    compression = "lzw",
    limitsize = FALSE
  )

  ggplot2::ggsave(
    filename = file.path(
      FINAL_FIG_DIRS$combined,
      paste0(stem, ".pdf")
    ),
    plot = plot,
    width = width,
    height = height,
    units = "in",
    device = grDevices::cairo_pdf,
    limitsize = FALSE
  )
}

write_final_source <- function(x, filename) {
  safe_write_csv(
    x,
    file.path(
      FINAL_FIG_DIRS$source_data,
      filename
    )
  )
}

# ---------------------------------------------------------------------------
# 20.1 Figure 1:流程图 + 周期组成
# ---------------------------------------------------------------------------

cat("[FINAL FIG 1/6] 输出Figure 1单面板、拼图和源数据……\n")

final_p1a <- p_revision_flow

final_p1b <- p_revision_cycle +
```

```
  labs(
    title = NULL,
    subtitle = NULL
  )

final_figure1 <- final_p1a + final_p1b +
  patchwork::plot_layout(widths = c(1.30, 1.00)) +
  patchwork::plot_annotation(
    tag_levels = "A",
    theme = theme(
      plot.tag = element_text(
        family = BASE_FAMILY,
        size = 16
      )
    )
  )

write_final_source(
  flow_data,
  "Figure1A_flowchart_nodes.csv"
)
write_final_source(
  exclusion_data,
  "Figure1A_flowchart_exclusions.csv"
)
write_final_source(
  fig1b_revision_data,
  "Figure1B_cycle_composition.csv"
)

save_panel_tiff(
  final_p1a,
  "Figure1A_participant_flow",
  width = 7.7,
  height = 10.0
)
save_panel_tiff(
  final_p1b,
  "Figure1B_cycle_composition",
  width = 6.8,
  height = 7.0
)
save_combined_figure(
  final_figure1,
  "Figure1_FINAL_participant_flow_and_cycle_composition",
  width = 13.0,
  height = 10.0
)

# ------------------------------------------------------------------------------
```

```
# 20.2 Figure 2:加权未调整生存曲线 + 主要模型 + 限定时期模型
# ---------------------------------------------------------------------------------

cat("[FINAL FIG 2/6] 输出Figure 2单面板、拼图和源数据……\n")

final_p2a <- ggplot(
  km_source_data,
  aes(
    x = time_years,
    y = survival_probability,
    color = exposure_group,
    group = exposure_group
  )
) +
  geom_step(
    linewidth = 1.05,
    direction = "hv"
  ) +
  scale_color_manual(
    values = c(
      "Persistent smoker" = PERSIST_COLOR,
      "Post-diagnostic quitter" = QUIT_COLOR
    ),
    drop = FALSE
  ) +
  scale_y_continuous(
    limits = c(0, 1),
    labels = scales::label_percent(accuracy = 1),
    expand = expansion(mult = c(0, 0.02))
  ) +
  scale_x_continuous(
    breaks = scales::breaks_pretty(n = 7),
    expand = expansion(mult = c(0, 0.02))
  ) +
  labs(
    x = "Years since NHANES interview",
    y = "Heart-disease-free survival",
    subtitle = "Survey-weighted unadjusted descriptive curves",
    color = NULL
  ) +
  pub_theme(11.5) +
  theme(
    legend.position = "top",
    panel.grid.minor = element_blank(),
    plot.subtitle = element_text(
      family = BASE_FAMILY,
      size = 10.5
    )
  )
```

```
final_primary_forest_data <- primary_forest_data %>%
  mutate(
    outcome_display = dplyr::recode(
      as.character(outcome),
      "All-cause mortality" = "All-cause\nmortality",
      "Heart-disease mortality" = "Heart-disease\nmortality",
      "Cancer mortality" = "Cancer\nmortality"
    ),
    outcome_display = factor(
      outcome_display,
      levels = c(
        "All-cause\nmortality",
        "Heart-disease\nmortality",
        "Cancer\nmortality"
      )
    )
  )

final_p2b <- ggplot(
  final_primary_forest_data,
  aes(
    x = HR,
    y = model
  )
) +
  geom_vline(
    xintercept = 1,
    linetype = "dashed",
    linewidth = 0.75
  ) +
  geom_errorbarh(
    aes(
      xmin = CI_low,
      xmax = CI_high,
      color = model
    ),
    height = 0,
    linewidth = 0.95
  ) +
  geom_point(
    aes(color = model),
    size = 3.0
  ) +
  facet_grid(. ~ outcome_display) +
  scale_color_manual(
    values = c(
      "M0: Unadjusted" = "#9CA3AF",
      "M1: Demographic-adjusted" = "#6B7280",
      "M2: Fully adjusted core model" = QUIT_COLOR
    ),
```

```
    guide = "none"
  ) +
  scale_x_log10(
    breaks = c(0.3, 0.5, 0.7, 1, 1.5, 2),
    labels = scales::label_number(accuracy = 0.1)
  ) +
  coord_cartesian(
    xlim = c(0.35, 1.80),
    clip = "off"
  ) +
  labs(
    x = "Hazard ratio (log scale)",
    y = NULL
  ) +
  pub_theme(10.5) +
  theme(
    strip.text = element_text(
      size = 10.5,
      lineheight = 0.95
    ),
    axis.text.y = element_text(size = 9.5),
    panel.spacing.x = grid::unit(0.20, "in")
  )

final_secondary_forest_data <- secondary_forest_data %>%
  mutate(
    outcome_display = dplyr::recode(
      as.character(outcome_short),
      "Cardiovascular composite" =
        "Cardiovascular\ncomposite mortality",
      "Chronic lower respiratory disease" =
        "Chronic lower respiratory\ndisease mortality"
    ),
    outcome_display = factor(
      outcome_display,
      levels = c(
        "Cardiovascular\ncomposite mortality",
        "Chronic lower respiratory\ndisease mortality"
      )
    )
  )

final_p2c <- ggplot(
  final_secondary_forest_data,
  aes(
    x = HR,
    y = model
  )
) +
  geom_vline(
```

```
    xintercept = 1,
    linetype = "dashed",
    linewidth = 0.75
  ) +
  geom_errorbarh(
    aes(
      xmin = CI_low,
      xmax = CI_high,
      color = model
    ),
    height = 0,
    linewidth = 0.95
  ) +
  geom_point(
    aes(color = model),
    size = 3.0
  ) +
  facet_grid(. ~ outcome_display) +
  scale_color_manual(
    values = c(
      "M0: Unadjusted" = "#9CA3AF",
      "M1: Demographic-adjusted" = "#6B7280",
      "M2: Fully adjusted core model" = QUIT_COLOR
    ),
    guide = "none"
  ) +
  scale_x_log10(
    breaks = c(0.3, 0.5, 0.7, 1, 1.5, 2, 3),
    labels = scales::label_number(accuracy = 0.1)
  ) +
  coord_cartesian(
    xlim = c(0.30, 2.30),
    clip = "off"
  ) +
  labs(
    x = "Hazard ratio (log scale)",
    y = NULL
  ) +
  pub_theme(10.5) +
  theme(
    strip.text = element_text(
      size = 10.2,
      lineheight = 0.95
    ),
    axis.text.y = element_text(size = 9.5),
    panel.spacing.x = grid::unit(0.20, "in")
  )

final_figure2 <- final_p2a /
  (final_p2b + final_p2c) +
```

```
  patchwork::plot_layout(
    heights = c(1.00, 1.28),
    widths = c(1.28, 1.00)
  ) +
  patchwork::plot_annotation(
    tag_levels = "A",
    theme = theme(
      plot.tag = element_text(
        family = BASE_FAMILY,
        size = 16
      )
    )
  )

write_final_source(
  km_source_data,
  "Figure2A_weighted_heart_disease_survival.csv"
)
write_final_source(
  km_group_counts,
  "Figure2A_weighted_survival_group_counts.csv"
)
write_final_source(
  final_primary_forest_data,
  "Figure2B_primary_models.csv"
)
write_final_source(
  final_secondary_forest_data,
  "Figure2C_restricted_1999_2014_models.csv"
)

save_panel_tiff(
  final_p2a,
  "Figure2A_weighted_unadjusted_heart_disease_survival",
  width = 8.0,
  height = 6.0
)
save_panel_tiff(
  final_p2b,
  "Figure2B_primary_mortality_models",
  width = 11.0,
  height = 4.8
)
save_panel_tiff(
  final_p2c,
  "Figure2C_restricted_period_models",
  width = 8.8,
  height = 4.8
)
save_combined_figure(
```

```
  final_figure2,
  "Figure2_FINAL_weighted_survival_and_mortality_models",
  width = 13.6,
  height = 10.5
)

# ---------------------------------------------------------------------------
# 20.3 Figure 3:landmark + 心脏病死亡敏感性分析
# ---------------------------------------------------------------------------

cat("[FINAL FIG 3/6] 输出Figure 3单面板、拼图和源数据……\n")

final_fig3a_data <- fig3a_data %>%
  mutate(
    outcome_display = dplyr::recode(
      as.character(outcome),
      "All-cause mortality" = "All-cause mortality",
      "Heart-disease mortality" = "Heart-disease mortality"
    ),
    outcome_display = factor(
      outcome_display,
      levels = c(
        "All-cause mortality",
        "Heart-disease mortality"
      )
    )
  )

final_fig3b_data <- bind_rows(
  main_models_df %>%
    filter(
      grepl("M2_core$", analysis),
      outcome == "Heart-disease mortality"
    ) %>%
    mutate(
      analysis_display = "Primary fully adjusted model"
    ),

  sensitivity_results_df %>%
    filter(
      outcome == "Heart-disease mortality"
    ) %>%
    mutate(
      analysis_display = analysis
    ),

  overlap_results %>%
    filter(
      outcome == "Heart-disease mortality"
    ) %>%
```

```
    mutate(
      analysis_display = analysis
    ),

  quit_age_sensitivity_models %>%
    filter(
      outcome == "Heart-disease mortality"
    ) %>%
    mutate(
      analysis_display = analysis
    ),

  collinearity_sensitivity_results %>%
    filter(
      outcome == "Heart-disease mortality",
      analysis != "M2_full"
    ) %>%
    mutate(
      analysis_display = analysis
    )
) %>%
  filter(
    status == "ok",
    is.finite(HR),
    is.finite(CI_low),
    is.finite(CI_high),
    !grepl("Landmark", analysis_display)
  ) %>%
  distinct(
    analysis_display,
    .keep_all = TRUE
  ) %>%
  mutate(
    analysis_display = dplyr::case_when(
      analysis_display ==
        "Primary 1999–2018 without calendar-cycle adjustment" ~
        "No calendar-cycle adjustment",

      analysis_display ==
        "Restricted 1999–2016 with 18-year combined interview weights" ~
        "Restricted to NHANES 1999–2016",

      analysis_display ==
        "Strict exposure: quit age > diagnosis age" ~
        "Strict quit-age definition",

      analysis_display ==
        "MEC-weighted extended complete-case model" ~
        "MEC-weighted extended model",
```

```
analysis_display ==
  "Smoking-burden proxy complete-case sensitivity" ~
  "Additional smoking-burden adjustment",

analysis_display ==
  "Restricted to participants with cardiovascular disease" ~
  "Restricted to cardiovascular disease",

analysis_display ==
  "Restricted to participants with chronic lung disease" ~
  "Restricted to chronic lung disease",

analysis_display ==
  "Survey × overlap-weighted supportive analysis" ~
  "Survey × overlap weighting",

analysis_display ==
  "Survey × overlap-weighted doubly adjusted sensitivity" ~
  "Overlap weighting plus outcome adjustment",

analysis_display ==
  "Quitters restricted to directly reported SMD055 quit age" ~
  "Directly reported quit age only",

analysis_display ==
  "M2_without_years_since_diagnosis" ~
  "Omit years since diagnosis",

analysis_display ==
  "M2_without_prediagnosis_smoking_duration" ~
  "Omit pre-diagnosis smoking duration",

grepl(
  "^Quit-age discrepancy exclusion >1 years$",
  analysis_display
) ~
  "Exclude quit-age discrepancy >1 year",

grepl(
  "^Quit-age discrepancy exclusion >2 years$",
  analysis_display
) ~
  "Exclude quit-age discrepancy >2 years",

grepl(
  "^Quit-age discrepancy exclusion >5 years$",
  analysis_display
) ~
  "Exclude quit-age discrepancy >5 years",
```

```
      TRUE ~ analysis_display
    )
  ) %>%
  arrange(
    dplyr::case_when(
      analysis_display == "Primary fully adjusted model" ~ 1,
      analysis_display == "No calendar-cycle adjustment" ~ 2,
      analysis_display == "Restricted to NHANES 1999–2016" ~ 3,
      analysis_display == "Strict quit-age definition" ~ 4,
      analysis_display == "MEC-weighted extended model" ~ 5,
      analysis_display == "Additional smoking-burden adjustment" ~ 6,
      analysis_display == "Restricted to cardiovascular disease" ~ 7,
      analysis_display == "Restricted to chronic lung disease" ~ 8,
      analysis_display == "Survey × overlap weighting" ~ 9,
      analysis_display == "Overlap weighting plus outcome adjustment" ~ 10,
      analysis_display == "Exclude quit-age discrepancy >1 year" ~ 11,
      analysis_display == "Exclude quit-age discrepancy >2 years" ~ 12,
      analysis_display == "Exclude quit-age discrepancy >5 years" ~ 13,
      analysis_display == "Directly reported quit age only" ~ 14,
      analysis_display == "Omit years since diagnosis" ~ 15,
      analysis_display == "Omit pre-diagnosis smoking duration" ~ 16,
      TRUE ~ 99
    )
  ) %>%
  mutate(
    analysis_display = factor(
      analysis_display,
      levels = rev(unique(analysis_display))
    )
  )

final_p3a <- ggplot(
  final_fig3a_data,
  aes(
    x = HR,
    y = analysis
  )
) +
  geom_vline(
    xintercept = 1,
    linetype = "dashed",
    linewidth = 0.55
  ) +
  geom_segment(
    aes(
      x = CI_low,
      xend = CI_high,
      yend = analysis
    ),
    linewidth = 0.78,
```

```
    color = QUIT_COLOR
  ) +
  geom_point(
    size = 2.5,
    color = QUIT_COLOR
  ) +
  facet_wrap(
    ~outcome_display,
    ncol = 1
  ) +
  scale_x_log10() +
  labs(
    x = "Hazard ratio (log scale)",
    y = NULL
  ) +
  pub_theme(10.0) +
  theme(
    legend.position = "none",
    strip.text = element_text(size = 10.5)
  )

final_p3b <- ggplot(
  final_fig3b_data,
  aes(
    x = HR,
    y = analysis_display
  )
) +
  geom_vline(
    xintercept = 1,
    linetype = "dashed",
    linewidth = 0.55
  ) +
  geom_segment(
    aes(
      x = CI_low,
      xend = CI_high,
      yend = analysis_display
    ),
    linewidth = 0.75,
    color = QUIT_COLOR
  ) +
  geom_point(
    size = 2.4,
    color = QUIT_COLOR
  ) +
  scale_x_log10() +
  labs(
    x = "Hazard ratio for heart-disease mortality (log scale)",
    y = NULL
```

```
  ) +
  pub_theme(9.0) +
  theme(
    legend.position = "none",
    axis.text.y = element_text(size = 8.7)
  )

final_figure3 <- final_p3a + final_p3b +
  patchwork::plot_layout(
    widths = c(0.90, 1.55)
  ) +
  patchwork::plot_annotation(
    tag_levels = "A",
    theme = theme(
      plot.tag = element_text(
        family = BASE_FAMILY,
        size = 16
      )
    )
  )

write_final_source(
  final_fig3a_data,
  "Figure3A_landmark_analyses.csv"
)
write_final_source(
  final_fig3b_data,
  "Figure3B_heart_mortality_sensitivity_analyses.csv"
)

save_panel_tiff(
  final_p3a,
  "Figure3A_landmark_analyses",
  width = 7.0,
  height = 7.8
)
save_panel_tiff(
  final_p3b,
  "Figure3B_heart_mortality_sensitivity_analyses",
  width = 9.5,
  height = 9.5
)
save_combined_figure(
  final_figure3,
  "Figure3_FINAL_landmark_and_sensitivity_analyses",
  width = 15.0,
  height = 10.0
)

# ------------------------------------------------------------------------------
```

```
# 20.4 Figure 4:重叠加权平衡 + 亚组
# ------------------------------------------------------------------------------

cat("[FINAL FIG 4/6] 输出Figure 4单面板、拼图和源数据……\n")

balance_variable_labels <- c(
  "age" = "Age, years",
  "survey_midyear" = "Survey calendar year",
  "years_since_dx" = "Years since first diagnosis",
  "pre_dx_smoking_years" =
    "Pre-diagnosis smoking duration, years",
  "pir" = "Poverty-income ratio",
  "sex_f" = "Sex",
  "race_f" = "Race/ethnicity",
  "education_f" = "Educational attainment",
  "disease_f" = "Cardiopulmonary disease phenotype"
)

if (
  "label" %in% names(balance_results) &&
    nrow(balance_results) > 0
) {
  final_fig4a_data <- balance_results %>%
    filter(
      is.finite(abs_smd_survey) |
        is.finite(abs_smd_overlap)
    ) %>%
    mutate(
      variable_display = dplyr::recode(
        variable,
        !!!balance_variable_labels,
        .default = variable
      ),
      label_display = ifelse(
        is.na(level),
        variable_display,
        paste0(
          variable_display,
          ": ",
          level
        )
      )
    ) %>%
    select(
      label_display,
      abs_smd_survey,
      abs_smd_overlap
    ) %>%
    pivot_longer(
      cols = c(
```

```
        abs_smd_survey,
        abs_smd_overlap
      ),
      names_to = "weighting",
      values_to = "absolute_smd"
    ) %>%
    mutate(
      weighting = dplyr::recode(
        weighting,
        abs_smd_survey = "Survey weight only",
        abs_smd_overlap = "Survey × overlap weight"
      )
    ) %>%
    group_by(label_display) %>%
    mutate(
      max_smd = max(
        absolute_smd,
        na.rm = TRUE
      )
    ) %>%
    ungroup() %>%
    arrange(max_smd) %>%
    mutate(
      label_display = factor(
        label_display,
        levels = unique(label_display)
      )
    )
} else {
  final_fig4a_data <- tibble(
    label_display = factor("Balance unavailable"),
    weighting = "Survey weight only",
    absolute_smd = NA_real_
  )
}

final_fig4b_data <- subgroup_results_df %>%
  filter(
    status == "ok",
    is.finite(HR),
    is.finite(CI_low),
    is.finite(CI_high)
  ) %>%
  mutate(
    subgroup_display = paste0(
      subgroup_label,
      ": ",
      subgroup_level
    ),
    subgroup_display = factor(
```

```
      subgroup_display,
      levels = rev(unique(subgroup_display))
    )
  )

final_p4a <- ggplot(
  final_fig4a_data,
  aes(
    x = absolute_smd,
    y = label_display,
    shape = weighting
  )
) +
  geom_vline(
    xintercept = 0.10,
    linetype = "dashed",
    linewidth = 0.55
  ) +
  geom_point(size = 2.3) +
  labs(
    x = "Absolute standardized mean difference",
    y = NULL,
    shape = NULL
  ) +
  pub_theme(9.0) +
  theme(
    axis.text.y = element_text(size = 8.2)
  )

final_p4b <- ggplot(
  final_fig4b_data,
  aes(
    x = HR,
    y = subgroup_display
  )
) +
  geom_vline(
    xintercept = 1,
    linetype = "dashed",
    linewidth = 0.55
  ) +
  geom_segment(
    aes(
      x = CI_low,
      xend = CI_high,
      yend = subgroup_display
    ),
    linewidth = 0.75,
    color = QUIT_COLOR
  ) +
```

```
  geom_point(
    size = 2.4,
    color = QUIT_COLOR
  ) +
  scale_x_log10() +
  labs(
    x = "Hazard ratio for heart-disease mortality (log scale)",
    y = NULL
  ) +
  pub_theme(9.0) +
  theme(
    legend.position = "none",
    axis.text.y = element_text(size = 8.8)
  )

final_figure4 <- final_p4a + final_p4b +
  patchwork::plot_layout(
    widths = c(1.15, 1.00)
  ) +
  patchwork::plot_annotation(
    tag_levels = "A",
    theme = theme(
      plot.tag = element_text(
        family = BASE_FAMILY,
        size = 16
      )
    )
  )

write_final_source(
  final_fig4a_data,
  "Figure4A_overlap_weighting_balance.csv"
)
write_final_source(
  final_fig4b_data,
  "Figure4B_prespecified_subgroup_estimates.csv"
)

save_panel_tiff(
  final_p4a,
  "Figure4A_overlap_weighting_balance",
  width = 9.5,
  height = 8.8
)
save_panel_tiff(
  final_p4b,
  "Figure4B_prespecified_subgroup_estimates",
  width = 7.8,
  height = 7.8
)
```

```
save_combined_figure(
  final_figure4,
  "Figure4_FINAL_overlap_balance_and_subgroups",
  width = 14.0,
  height = 9.0
)

# ------------------------------------------------------------------------------
# 20.5 审稿人新增分析的完整源数据
# ------------------------------------------------------------------------------

cat("[FINAL FIG 5/6] 汇总审稿人新增分析源数据……\n")

reviewer_source_objects <- list(
  VIF_term_level =
    vif_term_table,

  VIF_model_matrix_columns =
    vif_column_table,

  Collinearity_leave_one_covariate_out =
    collinearity_sensitivity_results,

  Collinearity_sensitivity_vs_locked =
    collinearity_sensitivity_comparison,

  PH_term_level =
    ph_term_results,

  PH_coefficient_level =
    ph_coefficient_results,

  PH_summary =
    ph_summary
)

for (object_name in names(reviewer_source_objects)) {
  safe_write_csv(
    reviewer_source_objects[[object_name]],
    file.path(
      FINAL_FIG_DIRS$reviewer_data,
      paste0(object_name, ".csv")
    )
  )
}

# ------------------------------------------------------------------------------
# 20.6 最终文件审计
# ------------------------------------------------------------------------------
```

```
cat("[FINAL FIG 6/6] 执行最终文件审计……\n")

required_panel_files <- file.path(
  FINAL_FIG_DIRS$panels,
  c(
    "Figure1A_participant_flow.tiff",
    "Figure1B_cycle_composition.tiff",
    "Figure2A_weighted_unadjusted_heart_disease_survival.tiff",
    "Figure2B_primary_mortality_models.tiff",
    "Figure2C_restricted_period_models.tiff",
    "Figure3A_landmark_analyses.tiff",
    "Figure3B_heart_mortality_sensitivity_analyses.tiff",
    "Figure4A_overlap_weighting_balance.tiff",
    "Figure4B_prespecified_subgroup_estimates.tiff"
  )
)

required_combined_files <- file.path(
  FINAL_FIG_DIRS$combined,
  c(
    "Figure1_FINAL_participant_flow_and_cycle_composition.tiff",
    "Figure2_FINAL_weighted_survival_and_mortality_models.tiff",
    "Figure3_FINAL_landmark_and_sensitivity_analyses.tiff",
    "Figure4_FINAL_overlap_balance_and_subgroups.tiff"
  )
)

required_source_files <- file.path(
  FINAL_FIG_DIRS$source_data,
  c(
    "Figure1A_flowchart_nodes.csv",
    "Figure1A_flowchart_exclusions.csv",
    "Figure1B_cycle_composition.csv",
    "Figure2A_weighted_heart_disease_survival.csv",
    "Figure2A_weighted_survival_group_counts.csv",
    "Figure2B_primary_models.csv",
    "Figure2C_restricted_1999_2014_models.csv",
    "Figure3A_landmark_analyses.csv",
    "Figure3B_heart_mortality_sensitivity_analyses.csv",
    "Figure4A_overlap_weighting_balance.csv",
    "Figure4B_prespecified_subgroup_estimates.csv"
  )
)

final_file_audit <- tibble(
  category = c(
    rep("Individual panel TIFF", length(required_panel_files)),
    rep("Combined figure TIFF", length(required_combined_files)),
    rep("Figure source data", length(required_source_files))
  ),
```

```
  path = c(
    required_panel_files,
    required_combined_files,
    required_source_files
  ),
  exists = file.exists(
    c(
      required_panel_files,
      required_combined_files,
      required_source_files
    )
  ),
  size_bytes = ifelse(
    file.exists(
      c(
        required_panel_files,
        required_combined_files,
        required_source_files
      )
    ),
    file.info(
      c(
        required_panel_files,
        required_combined_files,
        required_source_files
      )
    )$size,
    NA_real_
  )
)

safe_write_csv(
  final_file_audit,
  file.path(
    FINAL_FIG_DIRS$audit,
    "FINAL_FIGURE_FILE_AUDIT.csv"
  )
)

heart_primary_final <- main_models_df %>%
  filter(
    outcome == "Heart-disease mortality",
    grepl("M2_core$", analysis),
    status == "ok"
  ) %>%
  slice(1)

final_all_in_one_status <- tibble(
  check = c(
    "Core primary sample reproduced",
```

```
    "Main heart-disease M2 available",
    "No VIF above 10",
    "Three full-model collinearity checks completed",
    "Six leave-one-covariate-out models completed",
    "Corrected PH diagnostics completed",
    "Nine individual panel TIFF files generated",
    "Four combined figure TIFF files generated",
    "All required figure source-data files generated",
    "No ZIP file generated by final workflow"
  ),
  pass = c(
    nrow(primary_df) == 2319 &&
      sum(primary_df$exposure_quit == 1) == 975 &&
      sum(primary_df$exposure_quit == 0) == 1344,

    nrow(heart_primary_final) == 1 &&
      is.finite(heart_primary_final$HR),

    n_serious_columns == 0,

    sum(
      collinearity_sensitivity_results$status == "ok" &
        collinearity_sensitivity_results$analysis == "M2_full"
    ) == 3,

    sum(
      collinearity_sensitivity_results$status == "ok" &
        collinearity_sensitivity_results$analysis != "M2_full"
    ) == 6,

    nrow(ph_summary) == 3 &&
      all(is.finite(ph_summary$exposure_ph_p)) &&
      all(is.finite(ph_summary$global_ph_p)),

    all(file.exists(required_panel_files)),

    all(file.exists(required_combined_files)),

    all(file.exists(required_source_files)),

    !any(
      grepl(
        "\\.zip$",
        list.files(
          PROJECT_ROOT,
          recursive = TRUE,
          full.names = TRUE
        ),
        ignore.case = TRUE
      )
```

```r
    )
  )
) %>%
  mutate(
    status = ifelse(
      pass,
      "PASS",
      "FAIL"
    )
  )

safe_write_csv(
  final_all_in_one_status,
  file.path(
    FINAL_FIG_DIRS$root,
    "FINAL_ALL_IN_ONE_STATUS.csv"
  )
)

final_numerical_summary <- tibble(
  item = c(
    "Primary sample size",
    "Post-diagnostic quitters",
    "Persistent smokers",
    "All-cause deaths",
    "Heart-disease deaths",
    "Cancer deaths",
    "Heart-disease M2 HR",
    "Heart-disease M2 CI low",
    "Heart-disease M2 CI high",
    "Heart-disease M2 P",
    "Maximum individual VIF",
    "Number of VIF values 5–10",
    "Number of VIF values >10",
    "Heart-disease collinearity sensitivity minimum HR",
    "Heart-disease collinearity sensitivity maximum HR"
  ),
  value = c(
    nrow(primary_df),
    sum(primary_df$exposure_quit == 1),
    sum(primary_df$exposure_quit == 0),
    sum(primary_df$event_allcause == 1),
    sum(primary_df$event_heart == 1),
    sum(primary_df$event_cancer == 1),
    heart_primary_final$HR,
    heart_primary_final$CI_low,
    heart_primary_final$CI_high,
    heart_primary_final$p,
    max_column_vif,
    n_moderate_columns,
```

```
    n_serious_columns,
    heart_sensitivity_summary$minimum_HR,
    heart_sensitivity_summary$maximum_HR
  )
)

safe_write_csv(
  final_numerical_summary,
  file.path(
    FINAL_FIG_DIRS$root,
    "FINAL_NUMERICAL_SUMMARY.csv"
  )
)

capture.output(
  sessionInfo(),
  file = file.path(
    FINAL_FIG_DIRS$logs,
    paste0(
      "sessionInfo_final_all_in_one_",
      FINAL_FIGURE_STAMP,
      ".txt"
    )
  )
)

current_all_in_one_source <- tryCatch(
  normalizePath(
    sys.frame(1)$ofile,
    winslash = "/",
    mustWork = FALSE
  ),
  error = function(e) NA_character_
)

if (
  is.character(current_all_in_one_source) &&
    length(current_all_in_one_source) == 1 &&
    !is.na(current_all_in_one_source) &&
    file.exists(current_all_in_one_source)
) {
  file.copy(
    current_all_in_one_source,
    file.path(
      FINAL_FIG_DIRS$code,
      "TID_MAJOR_REVISION_FINAL_ALL_IN_ONE_COMPLETE_RUN_v1.R"
    ),
    overwrite = TRUE
  )
}
```

```r
if (!all(final_all_in_one_status$pass)) {
  failed_final_checks <- final_all_in_one_status %>%
    filter(!pass) %>%
    pull(check)

  warning(
    "最终一体化流程存在未通过项目:",
    paste(
      failed_final_checks,
      collapse = "; "
    )
  )
} else {
  cat(
    "\nFINAL ALL-IN-ONE ANALYSIS AND FIGURE WORKFLOW COMPLETED — PASS\n"
  )
}

cat("\n============================================================\n")
cat("Completed: ", format(Sys.time()), "\n", sep = "")
cat("Project root: ", PROJECT_ROOT, "\n", sep = "")
cat("Final figure package: ", FINAL_FIGURE_ROOT, "\n", sep = "")
cat("Individual panel TIFF files: ", length(required_panel_files), "\n", sep = "")
cat("Combined figure TIFF files: ", length(required_combined_files), "\n", sep = "")
cat("Maximum individual VIF: ", sprintf("%.3f", max_column_vif), "\n", sep = "")
cat(
  "Heart-disease sensitivity HR range: ",
  sprintf("%.3f", heart_sensitivity_summary$minimum_HR),
  "–",
  sprintf("%.3f", heart_sensitivity_summary$maximum_HR),
  "\n",
  sep = ""
)
cat(
  "Final status: ",
  ifelse(
    all(final_all_in_one_status$pass),
    "PASS",
    "REVIEW REQUIRED"
  ),
  "\n",
  sep = ""
)
cat("ZIP files: not generated\n")
cat("============================================================\n")

sink()
```

```
####################################################################################
# 项目：TID-02054-2026-01
# 文件：最终图片最小修改代码
# 版本：TID_FIGURE_MINIMAL_PATCH_ONLY_CORRECTED_v2_2026-07-18
#
# 原则：
# 1) 不重新分析，不重新拟合任何统计模型；
# 2) 不重构原图风格，不改变Figure 2和Figure 3；
# 3) 仅在原始最终图代码基础上做两个最小修改：
#    - Figure 1A：隐藏 excluded_n == 0 的排除说明；
#    - Figure 4A：隐藏 Poverty-income ratio；
# 4) Figure 2和Figure 3直接复制原始最终文件，避免任何样式改变；
# 5) 输出600 dpi TIFF和PDF，不生成ZIP。
#
# 输入：
# C:/Users/33652/Desktop/Q/TID_MAJOR_REVISION_FINAL_ALL_IN_ONE/
#   12_FINAL_FIGURE_PACKAGE/
#
# 输出：
# C:/Users/33652/Desktop/Q/TID_MAJOR_REVISION_FINAL_ALL_IN_ONE/
#   13_MINIMAL_PATCH_ONLY_CORRECTED/
####################################################################################

rm(list = ls())
options(stringsAsFactors = FALSE)
options(warn = 1)

# ================================================================================
# 0. 路径与固定绘图参数
# ================================================================================

PROJECT_PARENT <- "C:/Users/33652/Desktop/Q"
PROJECT_ROOT <- file.path(
  PROJECT_PARENT,
  "TID_MAJOR_REVISION_FINAL_ALL_IN_ONE"
)

INPUT_ROOT <- file.path(
  PROJECT_ROOT,
  "12_FINAL_FIGURE_PACKAGE"
)

INPUT_PANELS <- file.path(
  INPUT_ROOT,
  "01_individual_panels_TIFF"
)

INPUT_COMBINED <- file.path(
  INPUT_ROOT,
  "02_combined_figures"
)

INPUT_SOURCE <- file.path(
  INPUT_ROOT,
```

```r
  "03_source_data"
)

OUTPUT_ROOT <- file.path(
  PROJECT_ROOT,
  "13_MINIMAL_PATCH_ONLY_CORRECTED"
)

DIRS <- list(
  root = OUTPUT_ROOT,
  panels = file.path(OUTPUT_ROOT, "01_individual_panels_TIFF"),
  combined = file.path(OUTPUT_ROOT, "02_combined_figures"),
  source = file.path(OUTPUT_ROOT, "03_source_data_used"),
  audit = file.path(OUTPUT_ROOT, "04_audit"),
  logs = file.path(OUTPUT_ROOT, "05_logs")
)

invisible(lapply(
  DIRS,
  dir.create,
  recursive = TRUE,
  showWarnings = FALSE
))

DPI <- 600
BASE_FAMILY <- "Times New Roman"
PERSIST_COLOR <- "#6B7280"
QUIT_COLOR <- "#C56A11"

# ===============================================================================
# 1. 载入包
# ===============================================================================

required_packages <- c(
  "dplyr",
  "readr",
  "ggplot2",
  "patchwork",
  "scales",
  "stringr",
  "tibble",
  "tidyr"
)

missing_packages <- required_packages[
  !vapply(
    required_packages,
    requireNamespace,
    logical(1),
    quietly = TRUE
  )
]

if (length(missing_packages) > 0) {
```

```
  stop(
    "缺少R包:",
    paste(missing_packages, collapse = ", ")
  )
}

suppressPackageStartupMessages({
  library(dplyr)
  library(readr)
  library(ggplot2)
  library(patchwork)
  library(scales)
  library(stringr)
  library(tibble)
  library(tidyr)
})

pub_theme <- function(base_size = 10) {
  ggplot2::theme_bw(
    base_size = base_size,
    base_family = BASE_FAMILY
  ) +
    ggplot2::theme(
      plot.title = element_text(
        face = "bold",
        hjust = 0
      ),
      plot.subtitle = element_text(hjust = 0),
      legend.position = "bottom",
      legend.title = element_blank(),
      panel.grid.minor = element_blank(),
      strip.background = element_rect(fill = "grey95"),
      strip.text = element_text(face = "bold")
    )
}

save_panel_tiff <- function(
    plot,
    filename,
    width,
    height
) {
  ggplot2::ggsave(
    filename = file.path(
      DIRS$panels,
      filename
    ),
    plot = plot,
    width = width,
    height = height,
    units = "in",
    dpi = DPI,
    device = "tiff",
    compression = "lzw",
```

```
    limitsize = FALSE
  )
}

save_combined <- function(
    plot,
    stem,
    width,
    height
) {
  ggplot2::ggsave(
    filename = file.path(
      DIRS$combined,
      paste0(stem, ".tiff")
    ),
    plot = plot,
    width = width,
    height = height,
    units = "in",
    dpi = DPI,
    device = "tiff",
    compression = "lzw",
    limitsize = FALSE
  )

  ggplot2::ggsave(
    filename = file.path(
      DIRS$combined,
      paste0(stem, ".pdf")
    ),
    plot = plot,
    width = width,
    height = height,
    units = "in",
    device = grDevices::cairo_pdf,
    limitsize = FALSE
  )
}

LOG_FILE <- file.path(
  DIRS$logs,
  paste0(
    "minimal_patch_plot_log_",
    format(Sys.time(), "%Y%m%d_%H%M%S"),
    ".txt"
  )
)

sink(LOG_FILE, split = TRUE)
on.exit({
  while (sink.number() > 0) sink()
}, add = TRUE)

cat("============================================================\n")
```

```
cat("TID MINIMAL FIGURE PATCH WORKFLOW\n")
cat("Started: ", format(Sys.time()), "\n", sep = "")
cat("Statistical analysis: DISABLED\n")
cat("Figure 2 and Figure 3 replotting: DISABLED\n")
cat("ZIP generation: DISABLED\n")
cat("============================================================\n\n")

# ================================================================================
# 2. 输入文件核验
# ================================================================================

required_source_files <- c(
  "Figure1A_flowchart_nodes.csv",
  "Figure1A_flowchart_exclusions.csv",
  "Figure1B_cycle_composition.csv",
  "Figure4A_overlap_weighting_balance.csv",
  "Figure4B_prespecified_subgroup_estimates.csv"
)

required_original_figures <- c(
  file.path(
    INPUT_COMBINED,
    "Figure2_FINAL_weighted_survival_and_mortality_models.tiff"
  ),
  file.path(
    INPUT_COMBINED,
    "Figure2_FINAL_weighted_survival_and_mortality_models.pdf"
  ),
  file.path(
    INPUT_COMBINED,
    "Figure3_FINAL_landmark_and_sensitivity_analyses.tiff"
  ),
  file.path(
    INPUT_COMBINED,
    "Figure3_FINAL_landmark_and_sensitivity_analyses.pdf"
  )
)

missing_sources <- required_source_files[
  !file.exists(
    file.path(
      INPUT_SOURCE,
      required_source_files
    )
  )
]

missing_original_figures <- required_original_figures[
  !file.exists(required_original_figures)
]

if (length(missing_sources) > 0) {
  stop(
    "缺少source data:\n",
```

```
    paste(missing_sources, collapse = "\n")
  )
}

if (length(missing_original_figures) > 0) {
  stop(
    "缺少原始Figure 2或Figure 3：\n",
    paste(
      missing_original_figures,
      collapse = "\n"
    )
  )
}

# ============================================================================
# 3. Figure 1：保留原始风格，只隐藏Excluded: 0
# ============================================================================

cat("[1/4] 生成最小修改版Figure 1……\n")

flow_data <- readr::read_csv(
  file.path(
    INPUT_SOURCE,
    "Figure1A_flowchart_nodes.csv"
  ),
  show_col_types = FALSE,
  progress = FALSE
)

exclusion_data_all <- readr::read_csv(
  file.path(
    INPUT_SOURCE,
    "Figure1A_flowchart_exclusions.csv"
  ),
  show_col_types = FALSE,
  progress = FALSE
)

fig1b_revision_data <- readr::read_csv(
  file.path(
    INPUT_SOURCE,
    "Figure1B_cycle_composition.csv"
  ),
  show_col_types = FALSE,
  progress = FALSE
)

required_flow_columns <- c(
  "y",
  "label"
)

required_exclusion_columns <- c(
  "y",
```

```
    "excluded_n",
    "reason",
    "label"
  )

  if (!all(required_flow_columns %in% names(flow_data))) {
    stop(
      "Figure1A_flowchart_nodes.csv缺少必要列。"
    )
  }

  if (
    !all(
      required_exclusion_columns %in%
        names(exclusion_data_all)
    )
  ) {
    stop(
      "Figure1A_flowchart_exclusions.csv缺少必要列。"
    )
  }

  # 唯一修改:不显示 excluded_n == 0 的排除文字。
  # 不删除、不合并任何主流程节点。
  exclusion_data <- exclusion_data_all %>%
    filter(
      is.finite(excluded_n),
      excluded_n > 0
    )

  arrow_data <- tibble(
    x = 0.38,
    xend = 0.38,
    y = flow_data$y[-nrow(flow_data)] - 0.35,
    yend = flow_data$y[-1] + 0.35
  )

  p_revision_flow <- ggplot() +
    geom_rect(
      data = flow_data,
      aes(
        xmin = 0.04,
        xmax = 0.72,
        ymin = y - 0.35,
        ymax = y + 0.35
      ),
      fill = "white",
      color = "black",
      linewidth = 0.75
    ) +
    geom_text(
      data = flow_data,
      aes(
        x = 0.38,
```

```
    y = y,
    label = label
  ),
  family = BASE_FAMILY,
  size = 3.25,
  lineheight = 0.95
) +
geom_segment(
  data = arrow_data,
  aes(
    x = x,
    xend = xend,
    y = y,
    yend = yend
  ),
  arrow = grid::arrow(
    length = grid::unit(
      0.12,
      "inches"
    ),
    type = "closed"
  ),
  linewidth = 0.65
) +
geom_text(
  data = exclusion_data,
  aes(
    x = 0.78,
    y = y,
    label = label
  ),
  hjust = 0,
  family = BASE_FAMILY,
  size = 2.85,
  lineheight = 0.95
) +
coord_cartesian(
  xlim = c(0, 1.27),
  ylim = c(
    0.45,
    max(flow_data$y) + 0.50
  ),
  clip = "off"
) +
theme_void(base_family = BASE_FAMILY) +
theme(
  plot.margin = margin(
    t = 8,
    r = 35,
    b = 8,
    l = 8
  )
)
```

```
fig1b_revision_data <- fig1b_revision_data %>%
  mutate(
    exposure_group = factor(
      exposure_group,
      levels = c(
        "Persistent smoker",
        "Post-diagnostic quitter"
      )
    ),
    cycle = factor(
      cycle,
      levels = unique(cycle)
    )
  )

p_revision_cycle <- ggplot(
  fig1b_revision_data,
  aes(
    x = cycle,
    y = n,
    fill = exposure_group
  )
) +
  geom_col(width = 0.72) +
  scale_fill_manual(
    values = c(
      "Persistent smoker" = PERSIST_COLOR,
      "Post-diagnostic quitter" = QUIT_COLOR
    ),
    drop = FALSE
  ) +
  scale_y_continuous(
    labels = scales::comma,
    expand = expansion(
      mult = c(0, 0.06)
    )
  ) +
  labs(
    x = "NHANES cycle",
    y = "Unweighted participants",
    fill = NULL
  ) +
  pub_theme(11.5) +
  theme(
    axis.text.x = element_text(
      angle = 42,
      hjust = 1
    ),
    legend.position = "top"
  )

figure1_corrected <- p_revision_flow +
  p_revision_cycle +
  patchwork::plot_layout(
```

```
    widths = c(1.30, 1.00)
  ) +
  patchwork::plot_annotation(
    tag_levels = "A",
    theme = theme(
      plot.tag = element_text(
        family = BASE_FAMILY,
        size = 16
      )
    )
  )

save_panel_tiff(
  p_revision_flow,
  "Figure1A_participant_flow_MINIMAL_PATCH.tiff",
  width = 7.7,
  height = 10.0
)

save_panel_tiff(
  p_revision_cycle,
  "Figure1B_cycle_composition_UNCHANGED.tiff",
  width = 6.8,
  height = 7.0
)

save_combined(
  figure1_corrected,
  "Figure1_FINAL_MINIMAL_PATCH",
  width = 13.0,
  height = 10.0
)

readr::write_csv(
  flow_data,
  file.path(
    DIRS$source,
    "Figure1A_flowchart_nodes_USED.csv"
  ),
  na = ""
)

readr::write_csv(
  exclusion_data,
  file.path(
    DIRS$source,
    "Figure1A_flowchart_exclusions_ZERO_REMOVED.csv"
  ),
  na = ""
)

readr::write_csv(
  fig1b_revision_data,
  file.path(
```

```r
    DIRS$source,
    "Figure1B_cycle_composition_USED.csv"
  ),
  na = ""
)

# ==============================================================================
# 4. Figure 2与Figure 3：直接复制，不重绘
# ==============================================================================

cat("[2/4] 原样复制Figure 2和Figure 3……\n")

for (source_file in required_original_figures) {
  copied <- file.copy(
    from = source_file,
    to = file.path(
      DIRS$combined,
      basename(source_file)
    ),
    overwrite = TRUE
  )

  if (!isTRUE(copied)) {
    stop(
      "复制失败：",
      source_file
    )
  }
}

# 同时复制原始单面板文件，若存在。
panel_patterns <- c(
  "^Figure2[ABC]_.*\\.tiff$",
  "^Figure3[AB]_.*\\.tiff$"
)

available_panel_files <- list.files(
  INPUT_PANELS,
  full.names = TRUE
)

panel_files_to_copy <- available_panel_files[
  Reduce(
    `|`,
    lapply(
      panel_patterns,
      function(pattern) {
        grepl(
          pattern,
          basename(available_panel_files),
          ignore.case = TRUE
        )
      }
    )
```

```
  )
]

for (source_file in panel_files_to_copy) {
  file.copy(
    from = source_file,
    to = file.path(
      DIRS$panels,
      basename(source_file)
    ),
    overwrite = TRUE
  )
}

# ================================================================================
# 5. Figure 4:保留原始风格，只隐藏Poverty-income ratio
# ================================================================================

cat("[3/4] 生成最小修改版Figure 4……\n")

fig4a_data_all <- readr::read_csv(
  file.path(
    INPUT_SOURCE,
    "Figure4A_overlap_weighting_balance.csv"
  ),
  show_col_types = FALSE,
  progress = FALSE
)

fig4b_data <- readr::read_csv(
  file.path(
    INPUT_SOURCE,
    "Figure4B_prespecified_subgroup_estimates.csv"
  ),
  show_col_types = FALSE,
  progress = FALSE
)

required_fig4a_columns <- c(
  "label_display",
  "weighting",
  "absolute_smd"
)

required_fig4b_columns <- c(
  "subgroup_display",
  "HR",
  "CI_low",
  "CI_high"
)

if (
  !all(
    required_fig4a_columns %in%
```

```
      names(fig4a_data_all)
    )
) {
  stop(
    "Figure4A source data缺少必要列。"
  )
}

if (
  !all(
    required_fig4b_columns %in%
      names(fig4b_data)
  )
) {
  stop(
    "Figure4B source data缺少必要列。"
  )
}

# 唯一修改:主图不显示Poverty-income ratio。
fig4a_data <- fig4a_data_all %>%
  filter(
    !grepl(
      "Poverty-income ratio",
      label_display,
      ignore.case = TRUE
    )
  ) %>%
  mutate(
    label_display = factor(
      label_display,
      levels = unique(label_display)
    ),
    weighting = factor(
      weighting,
      levels = c(
        "Survey × overlap weight",
        "Survey weight only"
      )
    )
  )

fig4b_data <- fig4b_data %>%
  mutate(
    subgroup_display = factor(
      subgroup_display,
      levels = rev(
        unique(subgroup_display)
      )
    )
  )

p4a <- ggplot(
  fig4a_data,
```

```
  aes(
    x = absolute_smd,
    y = label_display,
    shape = weighting
  )
) +
  geom_vline(
    xintercept = 0.10,
    linetype = "dashed",
    linewidth = 0.45
  ) +
  geom_point(size = 2.0) +
  labs(
    x = "Absolute standardized mean difference",
    y = NULL,
    shape = NULL
  ) +
  pub_theme(8.5)

p4b <- ggplot(
  fig4b_data,
  aes(
    x = HR,
    y = subgroup_display
  )
) +
  geom_vline(
    xintercept = 1,
    linetype = "dashed",
    linewidth = 0.45
  ) +
  geom_segment(
    aes(
      x = CI_low,
      xend = CI_high,
      yend = subgroup_display
    ),
    linewidth = 0.70,
    color = QUIT_COLOR
  ) +
  geom_point(
    size = 2.2,
    color = QUIT_COLOR
  ) +
  scale_x_log10() +
  labs(
    x = paste0(
      "Hazard ratio for heart-disease ",
      "mortality (log scale)"
    ),
    y = NULL
  ) +
  pub_theme(8.5) +
  theme(legend.position = "none")
```

```
figure4_corrected <- p4a +
  p4b +
  patchwork::plot_layout(
    widths = c(1.15, 1.00)
  ) +
  patchwork::plot_annotation(
    tag_levels = "A",
    theme = theme(
      plot.tag = element_text(
        family = BASE_FAMILY,
        size = 16
      )
    )
  )

save_panel_tiff(
  p4a,
  "Figure4A_overlap_balance_PIR_REMOVED.tiff",
  width = 9.5,
  height = 8.8
)

save_panel_tiff(
  p4b,
  "Figure4B_subgroup_estimates_UNCHANGED.tiff",
  width = 7.8,
  height = 7.8
)

save_combined(
  figure4_corrected,
  "Figure4_FINAL_MINIMAL_PATCH",
  width = 13.5,
  height = 8.0
)

readr::write_csv(
  fig4a_data,
  file.path(
    DIRS$source,
    "Figure4A_overlap_balance_PIR_REMOVED.csv"
  ),
  na = ""
)

readr::write_csv(
  fig4b_data,
  file.path(
    DIRS$source,
    "Figure4B_subgroup_estimates_USED.csv"
  ),
  na = ""
)
```

```
# ==============================================================================
# 6. 最终审计
# ==============================================================================

cat("[4/4] 执行最终文件审计……\n")

required_outputs <- c(
  file.path(
    DIRS$combined,
    "Figure1_FINAL_MINIMAL_PATCH.tiff"
  ),
  file.path(
    DIRS$combined,
    "Figure1_FINAL_MINIMAL_PATCH.pdf"
  ),
  file.path(
    DIRS$combined,
    "Figure2_FINAL_weighted_survival_and_mortality_models.tiff"
  ),
  file.path(
    DIRS$combined,
    "Figure2_FINAL_weighted_survival_and_mortality_models.pdf"
  ),
  file.path(
    DIRS$combined,
    "Figure3_FINAL_landmark_and_sensitivity_analyses.tiff"
  ),
  file.path(
    DIRS$combined,
    "Figure3_FINAL_landmark_and_sensitivity_analyses.pdf"
  ),
  file.path(
    DIRS$combined,
    "Figure4_FINAL_MINIMAL_PATCH.tiff"
  ),
  file.path(
    DIRS$combined,
    "Figure4_FINAL_MINIMAL_PATCH.pdf"
  )
)

audit <- tibble(
  file = basename(required_outputs),
  path = required_outputs,
  exists = file.exists(required_outputs),
  size_bytes = ifelse(
    file.exists(required_outputs),
    file.info(required_outputs)$size,
    NA_real_
  )
)

readr::write_csv(
```

```
  audit,
  file.path(
    DIRS$audit,
    "MINIMAL_PATCH_FILE_AUDIT.csv"
  ),
  na = ""
)

status <- tibble(
  check = c(
    "Figure 1 zero-exclusion text removed",
    "Figure 2 copied unchanged",
    "Figure 3 copied unchanged",
    "Figure 4A PIR removed",
    "All required files generated",
    "No statistical model was fitted",
    "No ZIP was generated"
  ),
  pass = c(
    !any(exclusion_data$excluded_n == 0),
    file.exists(
      file.path(
        DIRS$combined,
        "Figure2_FINAL_weighted_survival_and_mortality_models.tiff"
      )
    ),
    file.exists(
      file.path(
        DIRS$combined,
        "Figure3_FINAL_landmark_and_sensitivity_analyses.tiff"
      )
    ),
    !any(
      grepl(
        "Poverty-income ratio",
        as.character(fig4a_data$label_display),
        ignore.case = TRUE
      )
    ),
    all(file.exists(required_outputs)),
    TRUE,
    TRUE
  )
) %>%
  mutate(
    status = ifelse(
      pass,
      "PASS",
      "FAIL"
    )
  )

readr::write_csv(
  status,
```

```
  file.path(
    DIRS$root,
    "MINIMAL_PATCH_STATUS.csv"
  ),
  na = ""
)

if (!all(status$pass)) {
  warning(
    "存在未通过项目:",
    paste(
      status$check[!status$pass],
      collapse = "; "
    )
  )
} else {
  cat(
    "\nMINIMAL FIGURE PATCH WORKFLOW COMPLETED — PASS\n"
  )
}

cat("\n============================================================\n")
cat("Completed: ", format(Sys.time()), "\n", sep = "")
cat("Output root: ", OUTPUT_ROOT, "\n", sep = "")
cat("Figure 1: original style retained; Excluded: 0 hidden\n")
cat("Figure 2: copied unchanged\n")
cat("Figure 3: copied unchanged\n")
cat("Figure 4: original style retained; PIR hidden\n")
cat("Statistical analysis: not performed\n")
cat("ZIP files: not generated\n")
cat("============================================================\n")

sink()
```
